# *Ex situ* generation of stoichiometric HCN and its application in the Pd-catalysed cyanation of aryl bromides: evidence for a transmetallation step between two oxidative addition Pd-complexes†
†Electronic supplementary information (ESI) available. CCDC 1504904–1504908. For ESI and crystallographic data in CIF or other electronic format see DOI: 10.1039/c7sc03912c


**DOI:** 10.1039/c7sc03912c

**Published:** 2017-10-06

**Authors:** Steffan K. Kristensen, Espen Z. Eikeland, Esben Taarning, Anders T. Lindhardt, Troels Skrydstrup

**Affiliations:** a Carbon Dioxide Activation Center (CADIAC) , The Interdisciplinary Center (iNANO) , Department of Chemistry , Aarhus University , Gustav Wieds Vej 14 , 8000 Aarhus , Denmark . Email: ts@chem.au.dk; b Center for Materials Crystallography , The Interdisciplinary Center (iNANO) , Department of Chemistry , Aarhus University , Langelandsgade 140 , 8000 Aarhus , Denmark; c Haldor Topsøe A/S , New Business R&D , Nymøllevej 55, 2800 Kgs , Lyngby , Denmark; d Carbon Dioxide Activation Center (CADIAC) , The Interdisciplinary Center (iNANO) , Biological and Chemical Engineering , Department of Engineering , Aarhus University , Finlandsgade 22 , 8200 Aarhus N , Denmark

## Abstract

A protocol for the Pd-catalysed cyanation of aryl bromides using near stoichiometric and gaseous hydrogen cyanide is reported for the first time. A two-chamber reactor was adopted for the safe liberation of *ex situ* generated HCN in a closed environment, which proved highly efficient in the Ni-catalysed hydrocyanation as the test reaction. Subsequently, this setup was exploited for converting a range of aryl and heteroaryl bromides (28 examples) directly into the corresponding benzonitriles in high yields, without the need for cyanide salts. Cyanation was achieved employing the Pd(0) precatalyst, P(*t*Bu)_3_-Pd-G3 and a weak base, potassium acetate, in a dioxane-water solvent mixture. The methodology was also suitable for the synthesis of ^13^C-labelled benzonitriles with *ex situ* generated ^13^C-hydrogen cyanide. Stoichiometric studies with the metal complexes were undertaken to delineate the mechanism for this catalytic transformation. Treatment of Pd(P(*t*Bu)_3_)_2_ with H^13^CN in THF provided two Pd-hydride complexes, (P(*t*Bu)_3_)_2_Pd(H)(^13^CN), and [(P(*t*Bu)_3_)Pd(H)]_2_Pd(^13^CN)_4_, both of which were isolated and characterised by NMR spectroscopy and X-ray crystal structure analysis. When the same reaction was performed in a THF : water mixture in the presence of KOAc, only (P(*t*Bu)_3_)_2_Pd(H)(^13^CN) was formed. Subjection of this cyano hydride metal complex with the oxidative addition complex (P(*t*Bu)_3_)Pd(Ph)(Br) in a 1 : 1 ratio in THF led to a transmetallation step with the formation of (P(*t*Bu)_3_)_2_Pd(H)(Br) and ^13^C-benzonitrile from a reductive elimination step. These experiments suggest the possibility of a catalytic cycle involving initially the formation of two Pd(ii)-species from the oxidative addition of L_*n*_Pd(0) into HCN and an aryl bromide followed by a transmetallation step to L_*n*_Pd(Ar)(CN) and L_*n*_Pd(H)(Br), which both reductively eliminate, the latter in the presence of KOAc, to generate the benzonitrile and L_*n*_Pd(0).

## Abstract 2

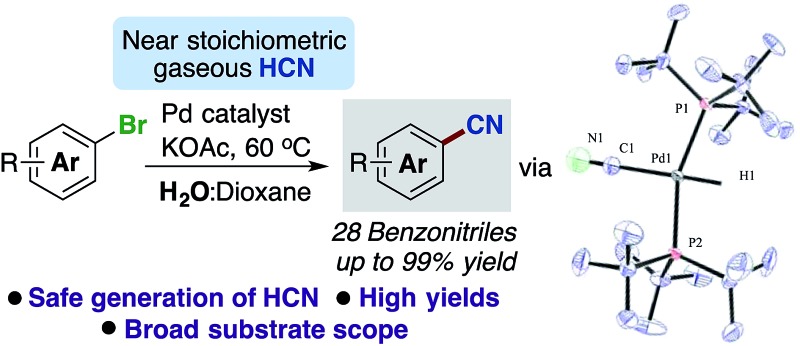
We demonstrate how hydrogen cyanide can be exploited for the cyanation of aryl bromides with Pd-catalysis.

## Introduction

**Scheme 1:**
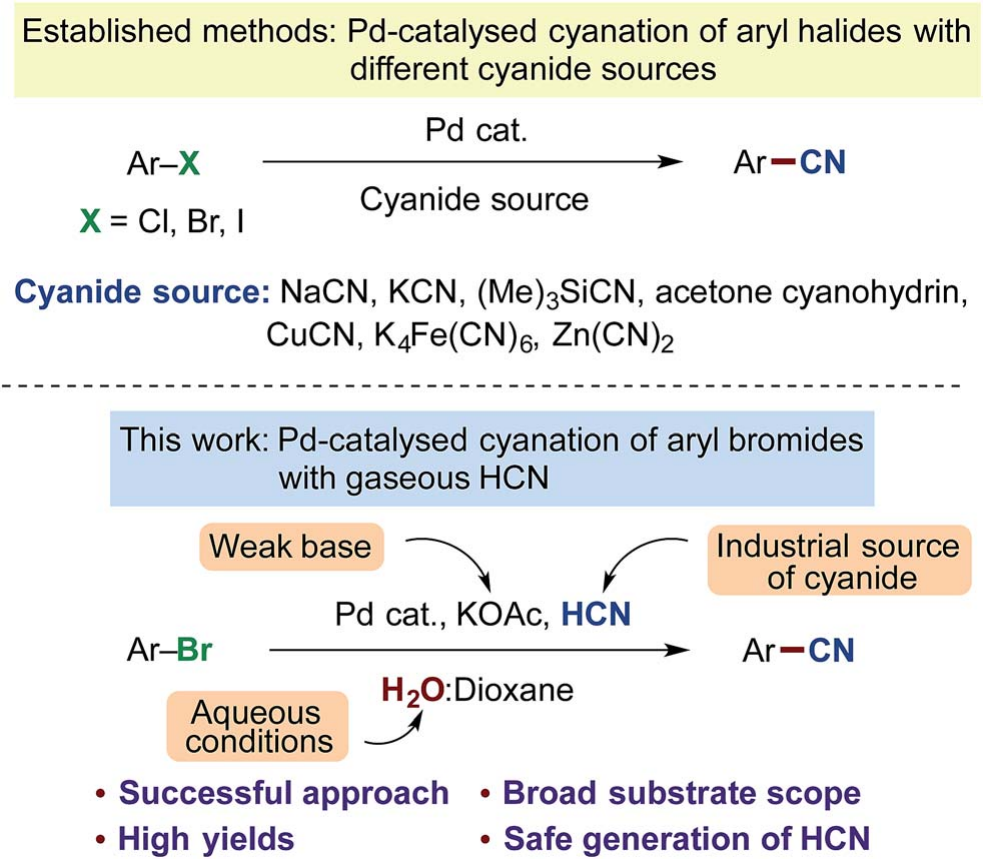
Previous developments in Pd-catalysed cyanation protocols and our new approach applying gaseous HCN.

Benzonitriles are a valuable class of compounds with a broad range of applications serving as important substructures in many agrochemicals, pharmaceuticals, dyes, polymers and other organic materials.^1^ The nitrile group is also a precursor to a variety of important functionalities such as aldehydes, ketones, carboxamides and carboxylates.^2^ Conventional means for the instalment of a cyanide group onto an aromatic ring include the Rosenmund-von Braun or Sandmeyer reactions applying stoichiometric CuCN with aryl halides or diazonium salts.^3^ Yet, in the last decade, transition metal catalysed cyanations of aromatic halides with metal complexes based on copper,^4^ nickel^5^ and palladium^6^,^7^ have held a key position. In particular, the protocols based on Pd-catalysis through the extensive work by Beller,^7^ Grushin^8^ and Buchwald^9^ are characterised by relatively mild reaction conditions and good functional group tolerance. A variety of different cyanide sources have been applied including salts or derivatives such as NaCN,^8^,^10^ KCN,6a,c,d,^11^ (Me)_3_SiCN,^12^ acetone cyanohydrin,^13^ K_4_Fe(CN)_6_,9a,^14^ Zn(CN)_2_,9b,^15^ CuCN,^16^*etc.* (Scheme 1). However, many of these sources also have their disadvantages. For example, metal contaminants can become an issue with the use of potassium ferrocyanide, whereas with the cyanohydrin of acetone, this chemical must be stored carefully and is approx. 20 times more costly than potassium cyanide.‡
‡According to the Sigma-Aldrich catalogue, 5 g of acetone cyanohydrin costs the same as approximately 100 g of potassium cyanide. Furthermore, considering the fact that all of these cyanide precursors originate from hydrogen cyanide (HCN), which is produced on a million-ton scale size *via* the Andrussow^17^ and BMA-Degussa processes^18^ from natural gas and ammonia or as a byproduct in acrylonitrile production,^18^,^19^ there could be an interest in the development of a cyanation process of aryl halides exploiting this original source of cyanide, namely gaseous HCN.^20^

Only three reports are found in the literature applying HCN directly or by its *in situ* formation. In 2015, Buchwald and Hooker reported a ^11^C-cyanation study for the synthesis of aromatic cyanides with applications for positron emission tomography (PET).^21^ Here, submicromolar amounts of gaseous H^11^CN dissolved in THF were introduced to a 1000-fold excess of preformed oxidative addition palladium(ii) complex, affording the desired ^11^C-benzonitriles nearly instantaneously. A follow-up paper by the same two groups demonstrated the possibilities of this approach for the ^11^C-labelling of unprotected peptides.^22^ Alternatively, Beller and co-workers reported the *in situ* release of HCN from acetone cyanohydrin although this was carried out in the presence of sodium carbonate, which undoubtedly leads to the direct formation of the reactive cyanide anion species.^13^

Nevertheless, there is a reluctance to apply HCN (b.p. 27 °C) as a cyanation reagent in an academic setting or an R&D laboratory, which is undoubtedly linked to the high toxicity and explosive nature of this gaseous one-carbon reagent, thereby complicating its handling, storage and transportation.^23^ Another challenge in the use of hydrogen cyanide as a cyanation reagent is how to control its dosage in stoichiometric amounts as Pd-catalysed cyanations of aryl halides are particularly sensitive to the cyanide concentrations. The strong binding of cyanide anion to transition metals can lead to catalyst deactivation if present in elevated concentrations.^7^,^24^ With other cyanide reagents, this complication is normally addressed by their slow addition to the reaction mixture, slow mass transport of the cyanide anion by specific water/organic solvents mixtures or by use of cyanide complexes with low solubilities.^8^,9b,^13^,^14^ Grushin and co-workers have published impressive and detailed studies on the mechanism of cyanide induced catalyst deactivation in the Pd-catalysed cyanation of aromatic halides. In their work, they report that addition of an excess of ^13^C-labelled potassium cyanide (K^13^CN) to different intermediates of the catalytic cycle in the Pd-catalysed cyanation of iodobenzene led to coordinatively saturated and catalytically inactive palladium complexes such as [Pd(CN)_4_]^2–^, [HPd(CN)_3_]^2–^ or [ArPd(CN)_3_]^2–^.^25^ More interestingly, the same group revealed that traces of water in the reaction mixture combined with K^13^CN forms H^13^CN that immediately undergoes oxidative addition with Pd(0) leading to the same coordinatively saturated and off-cycle palladium complexes with concurrent formation of hydrogen gas.^26^

In this paper, we report on the direct use of industrially important hydrogen cyanide in the palladium-catalysed cyanation of (hetero)aromatic bromides (Scheme 1). These results deviate from conventional wisdom, since Grushin's earlier work demonstrated the propensity of palladium(0) to undergo fast oxidative addition into the H–CN bond shutting down the catalytic cycle. And yet, our results demonstrate that conditions can be identified whereby the presence of HCN does not terminate the cyanation reaction, but enhances the reactivity vis-à-vis cyanide salts. Secondly and most important, we describe a simple and safe setup whereby HCN is delivered in stoichiometric amounts by *ex situ* generation in a two-chamber reactor, thereby providing a simple and safe setting for handling gaseous HCN in small-scale reactions. We demonstrate the usefulness of this setup not only for the Pd-catalysed cyanation of aryl bromides, but also for the Ni-mediated hydrocyanation of styrenes as a test reaction. With respect to the cyanation reactions good functional group tolerance was obtained, and the method proved amenable to scale-up, but also to carbon-13 isotope labelling applying H^13^CN. Surprisingly, the developed conditions proved to be dependent on water as the co-solvent and the presence of a weak base, KOAc. In their absence, catalytic shutdown was observed, thereby indicating the operation of an alternative mechanism to Pd-catalysed cyanations applying cyanide salts. Studies and isolation of the oxidative addition complexes suggest a mechanism involving the transmetallation between two palladium(ii) species, both formed by the oxidative addition of palladium(0) into either HCN or the aryl bromide electrophile.

## Results and discussion

### Hydrogen cyanide releasing studies

**Scheme 2:**
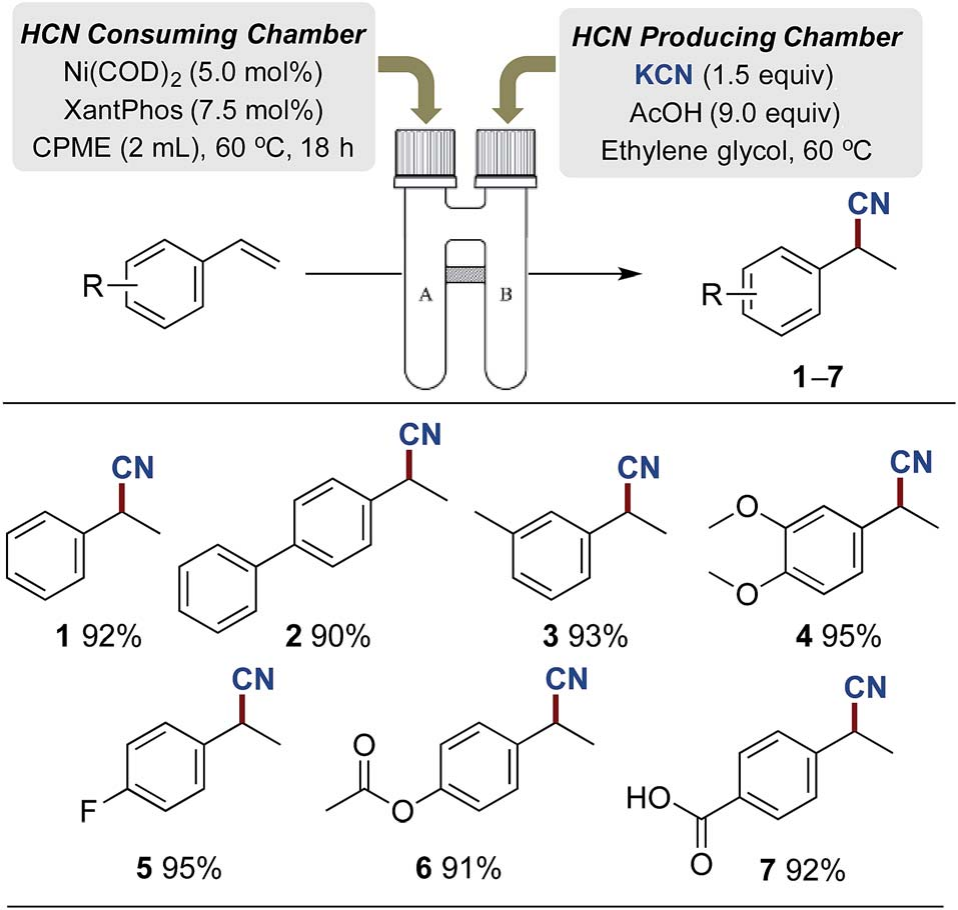
Ni-catalysed hydrocyanation of styrenes employing a two-chamber system with *ex situ* generated HCN. ^a^Chamber A: styrene (1.0 mmol), Ni(COD)_2_ (5.0 mol%), XantPhos (7.5 mol%) and CPME (2 mL). Chamber B: KCN (1.5 mmol), ethylene glycol (1 mL), AcOH (9.0 mmol). Isolated yields are given as an average of 2 runs.

In order to provide a system for the controlled dosage of hydrogen cyanide in stoichiometric quantities, we envisioned that the two-chamber system previously utilised for the *ex situ* liberation of carbon monoxide,^27^ hydrogen^28^ and ethylene^29^ from specific nongaseous precursors could be exploited. Such an approach would provide a simple and safe setup without the direct handling of HCN gas. Hence, our focus was first directed to identifying a suitable system for the generation of HCN gas under a closed environment, and the well-established nickel-catalysed hydrocyanation of olefins was exploited as a test system for the optimisation.^30^–^32^ After considerable experimentation on the hydrocyanation of styrene (see ESI†), we finally adopted the following conditions as illustrated in Scheme 2. A combination of KCN, ethylene glycol and AcOH provided the desired release of gaseous HCN. Ethylene glycol serves a dual purpose acting both as solvent while simultaneously ensuring separation of the AcOH from KCN until stirring is initiated. By utilising this setup for the HCN producing chamber in combination with the Ni-catalysed hydrocyanation conditions in the second chamber (Ni(COD)_2_, XantPhos), a range of different styrenes bearing both electron donating and withdrawing groups could successfully be hydrocyanated to products **1–7** in near quantitative yields. Full conversion of all styrene derivatives was attained applying only 1.5 equivalents of KCN for the production of HCN, indicating the effectiveness of this gas generator.

### Studies on the cyanation of (hetero)aryl bromides

With a simple and convenient system for the release of gaseous HCN in hand, we next turned our attention towards the Pd-catalysed cyanation of (hetero)aryl bromides. As depicted in the scheme of Table 1, initial optimisation results were carried out with 4-bromoanisole. This study revealed that reaction conditions consisting of a catalyst formed from Pd(dba)_2_ with P(*t*Bu)_3_, in the presence of hydrogen cyanide (1.5 equiv.), a weak base such as KOAc, and a solvent mixture of water and dioxane could generate 4-methoxybenzonitrile (**8**) with a conversion of 78% according to the ^1^H NMR analysis of the crude reaction mixture (Table 1, entry 1). P(*t*Bu)_3_ as a ligand proved to be superior to other monodentate and bidentate ligands (entries 2–6; see ESI† section for full optimisation studies), which is in accord with earlier results reported by Grushin and co-workers.^8^,^26^ In general, weak bases such as KOAc and NaOAc provided better conversions to the desired 4-methoxybenzonitrile, whereas stronger bases including Cy_2_NMe, DBU and Et_3_N all proved inferior (entries 7–9). Increasing to three equivalents of KOAc provided a slight increase in the conversion to 82% (entry 10). The efficiency of the reaction could also be increased further exploiting the Buchwald pre-catalyst, P(*t*Bu)_3_-Pd-G3, providing a 90% isolated yield of benzonitrile **8** after column chromatography (entry 11). Control experiments with omission of either base or palladium precursors resulted in low or no conversion, resp.

**Table 1:** Optimisation of the Pd-catalysed cyanation of aryl bromides using gaseous HCN^*a*^

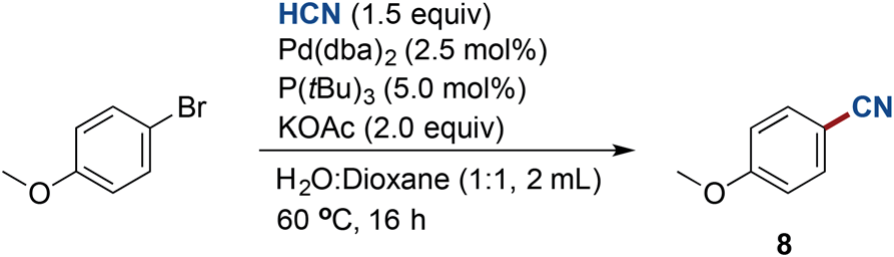		
Entry	Deviation from lead conditions	Conversion^*b*^ (%)
1	None	78
2	Ligand: PCy_3_	0
3	Ligand: XPhos	60
4	Ligand: *t*Bu-XPhos	74
5	Ligand: DPPF	0
6	Ligand: XantPhos (2.5 mol%)	0
7	Base: Cy_2_NMe	38
8	Base: DBU	5
9	Base: Et_3_N	67
10	Base: KOAc (3.0 equiv.)	82
**11**	**Pd/ligand: P(*t*Bu)**_**3**_ **-Pd-G3 KOAc (3.0 equiv.)**	**90 (90)** ^*c*^
12	Pd/ligand: P(*t*Bu)_3_-Pd-G3 solvent: THF	77
13	Pd/ligand: P(*t*Bu)_3_-Pd-G3 solvent: Toluene	61

^*a*^Chamber A: 4-bromoanisole (1.0 mmol), Pd(dba)_2_ (2.5 mol%), P(*t*Bu)_3_ (5.0 mol%), KOAc (2.0 mmol) in dioxane (1 mL) and H_2_O (1 mL). Chamber B: KCN (1.5 mmol), ethylene glycol (1 mL) and AcOH (9.0 mmol).

^*b*^Determined by ^1^H NMR using mesitylene as an internal standard. P(*t*Bu)_3_-Pd-G3 = third generation Buchwald precatalyst with the tri-*tert*-butylphosphine ligand.

^*c*^Isolated yield.

Different water : solvent combinations were tested and dioxane proved to be the solvent of choice (entries 12 and 13, see also ESI†). Notably, the presence of water as the co-solvent is of key importance as similar experiments applying only dioxane as the solvent resulted in complete catalytic shutdown. In our scope studies, we later discovered that a 2 : 1 mixture of water and dioxane provided higher yields for certain substrates, and hence this ratio was used for all subsequent cyanations. Not surprisingly and in accord with Grushin's previous results, carrying out the Pd-catalysed cyanation in a single chamber reactor applying KCN directly with or without the addition of acetic acid under the conditions developed led to no formation of **8** (result not shown).

**Scheme 3:**
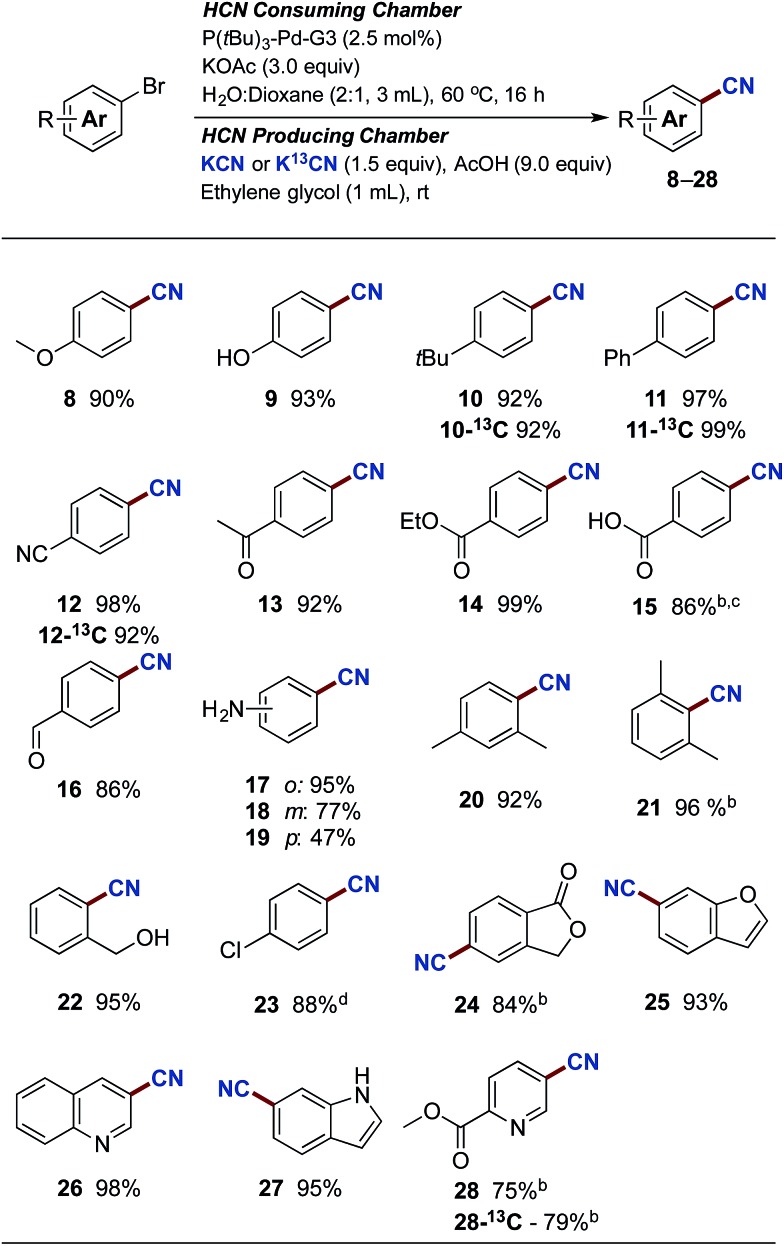
Pd-catalysed cyanation of aryl bromides. ^a^Chamber A: aryl bromide (1.0 mmol), P(*t*Bu)_3_-Pd-G3 (2.5 mol%) and KOAc (3.0 mmol) in dioxane (1 mL) and H_2_O (2 mL). Chamber B: KCN (1.5 mmol) or K^13^CN (1.5 mmol) ethylene glycol (1 mL) and AcOH (9 mmol). Isolated yields are given as an average of 2 runs. ^b^5.0 mol% catalyst used. ^c^4.0 equiv. of KOAc used. ^d^HCN consuming chamber only heated to 45 °C.

With the optimised reaction conditions in hand, we set out to explore the scope of the Pd-catalysed cyanation using gaseous HCN. All yields are reported as an average of two runs and the results are depicted in Scheme 3. Aryl bromides carrying electron donating substituents were initially examined. Methoxy-, hydroxy-, alkyl- and aryl-substituted aryl bromides provided the desired compounds in high yields ranging from 90% to 97% (compounds **8–11**). Electron withdrawing substituents such as cyano, acyl and carboxylate afforded compounds **12**, **13** and **14** in yields attaining quantitative. Even *p*-bromobenzoic acid underwent successful coupling affording *p*-cyanobenzoic acid (**15**) in an 86% yield, using 4 equiv. of KOAc combined with 5 mol% of Buchwald's precatalyst. The use of phenol and benzoic acid derivatives in the Pd-catalysed cyanation with NaCN were previously shown by Ushkov and Grushin to lead to catalyst deactivation due to the formation of HCN.^8^*p*-Bromobenzaldehyde also proved reactive with the isolation of **16** in a somewhat lower yield. This slight reduction in isolated yield can possibly be explained by competing benzoin condensation of both starting material and product. The substitution pattern of bromoanilines interfered significantly with the developed conditions and installment of the cyano-functionality onto 2-, 3-, and 4-bromoaniline was achieved in yields of 95%, 77% and 47% respectively (compounds **17–19**). Aryl bromides displaying *ortho*-substitutions were also effective for these substitution reactions as illustrated with compounds **17** and **20–22**, all isolated in yields between 92% and 96%. However, 2-bromo-1,3-dimethylbenzene did require an increase of the catalyst loading to achieve the high 96% isolated yield of **21**. Lowering the reaction temperature of the HCN consuming chamber, from 60 °C to 45 °C, ensured a chemoselective activation of the aromatic bromide in the presence of the *p*-chloride affording **23** in an 88% isolated yield. Subsequently, an aryl bromide possessing a benzylic alcohol, and five heteroaromatic bromides were tested under the developed conditions. All underwent successful cyanation to afford the desired target molecules in yields ranging from 75% to 95% (compounds **22**, **24–28**). It should be mentioned though that despite the successful coupling of these handful of heteroaryl bromides, two other heterocycles tested such as 2-acetyl-5-bromothiophene and 3-bromobenzothiophene failed to provide the corresponding nitrile product. The reasons for this catalytic shutdown are not completely understood (see Discussion in next section). Finally, attempts to perform catalytic cyanation on 4-biphenyl triflate, applying the developed protocol, were non-rewarding with no conversion observed for this electrophile.

Next, attention was directed to implementing this developed protocol for ^13^C-labelling. By simply substituting KCN with its ^13^C-labelled counterpart under otherwise unchanged conditions, direct access to isotopically labeled benzonitrile derivatives were achieved. Four entries from Scheme 3 were selected for labelling, and all compounds were isolated in near identical yields to the unlabeled compounds (compounds **10-^13^C**, **11-^13^C**, **12-^13^C** and **28-^13^C**).

**Scheme 4:**
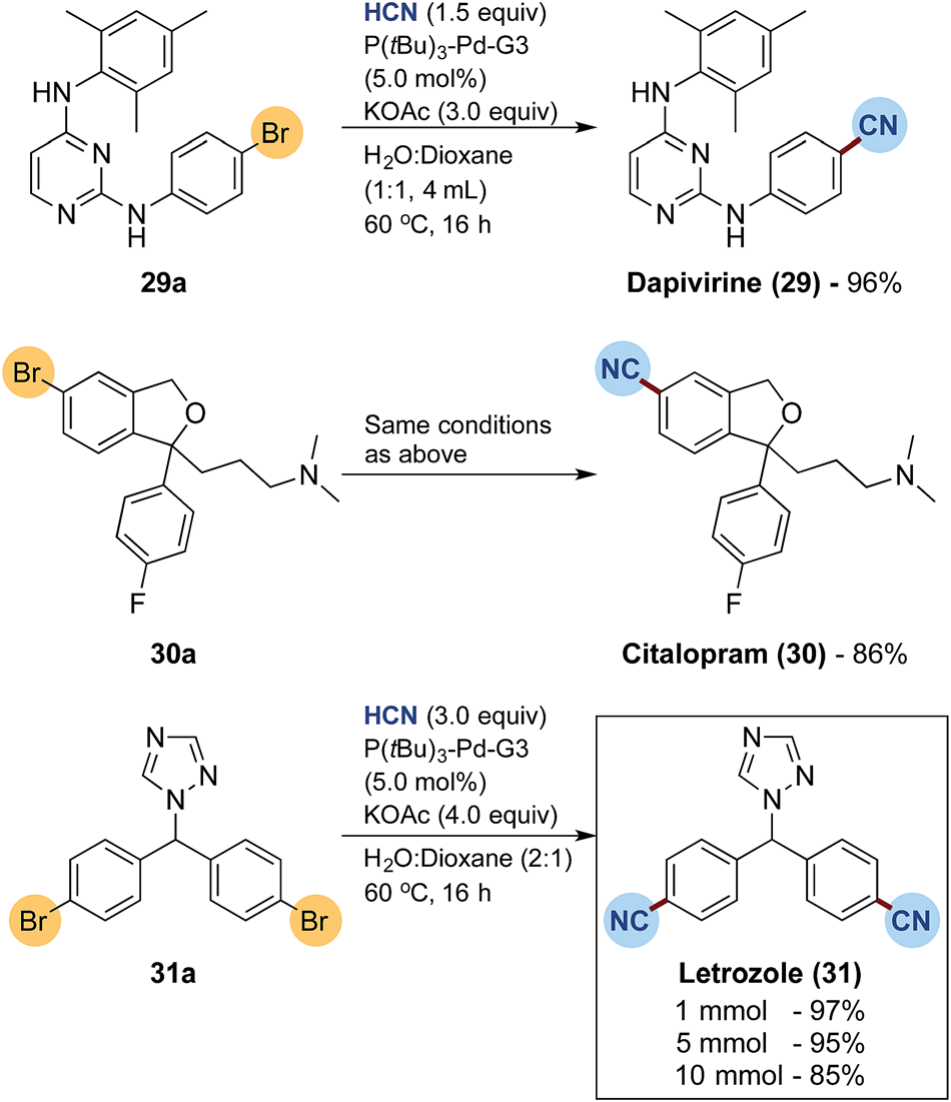
Synthesis of pharmaceuticals by Pd-catalysed cyanation and scale up studies. ^a^For reactions on 1.0 mmol scale, yields are an average of two runs.

Finally, the developed protocol was tested for the synthesis of three active pharmaceutical ingredients, namely dapivirine (reverse transcriptase inhibitor), citalopram (serotonin reuptake inhibitor) and letrozole (non-steroidal aromatase inhibitor), the results of which are depicted in Scheme 4.^33^ Dapivirine (**29**) and citalopram (**30**) were obtained in satisfactory 96% and 86% isolated yields, respectively, from the corresponding bromide precursors applying 5.0 mol% of the Buchwald precatalyst. For the synthesis of letrozole (**31**) a double cyanation is required with the dibromide **31a**. Doubling the loading of KCN for HCN release to 3 equivalents combined with an increase in KOAc to 4 equivalents, gratifyingly afforded the dicyanide **31** in an excellent 97% isolated yield again with 5.0 mol% precatalyst. The synthesis of letrozole was then attempted on a fivefold scale combined with a 100 mL two-chamber reactor to afford a near identical isolated yield of 95%. Finally, scaling up to 10 mmol in a 200 mL two-chamber reactor, with an HCN release from 30 mmol of KCN, corresponding to more than 700 mL of gaseous HCN, afforded the desired pharmaceutical in a good 85% isolated yield.

### Mechanistic investigations

Having established the scope of the Pd-catalysed cyanation of (hetero)aryl bromides with hydrogen cyanide, some questions still remained. Given a pKa difference in water for acetic acid (4.76) and HCN (9.21) of almost 4.5 units, indicates that HCN is potentially the reactive species in solution and not the free cyanide anion. However, previous reports by Grushin and co-workers clearly demonstrated that the presence of free HCN formed from the protonation of KCN with trace amounts of water, quickly leads to shutdown of the catalytic activity.^8^,^26^ Still this deactivation is a combined result of Pd(0) undergoing oxidative addition into the H–CN bond followed by trapping of this species with excess cyanide anion to generate off-cycle palladium(ii) cyanide complexes. Furthermore, there was not a clear indication why certain heteroaryl bromides were successful coupling partners with HCN, whereas others were not. With the exception of the protocol developed by Buchwald and Hooker, in which H^11^CN is used to form PET tracers, no reports are found on palladium-catalysed cyanation of aryl halides using gaseous HCN as the reactant.^21^ Although it should be noted under the conditions used for ^11^C-isotope-labelling, the Pd(ii)-aryl complex is in an approx. 1000-fold excess compared to the H^11^CN generated. With this in mind it was decided to take a closer look at the possible mechanism for the developed protocol.

**Scheme 5:**
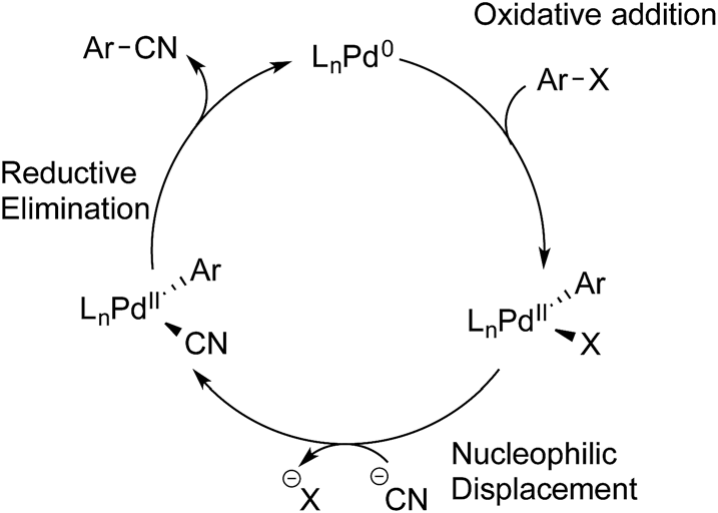
Accepted mechanism for the cyanation of aryl halides with cyanide salts.

The overall accepted mechanism for the Pd-catalysed cyanation using sodium or potassium cyanide is believed to go through an initial oxidative addition of the Pd(0) complex into the aryl halide, nucleophilic displacement by cyanide on the formed palladium(ii) centre, and lastly a reductive elimination furnishing the desired benzonitrile (Scheme 5).6h,^7^,^8^,^26^ When metal complexes such as Zn(CN)_2_ are utilised, a change in mechanism occurs, whereby nucleophilic substitution is replaced with a transmetallation step.9b,^34^

During the optimisation of the catalytic system, different bases were tested (Table 1, entries 7–10). A clear trend can be abstracted from this base screening, where an increase in base strength leads to a decrease in conversion. This can be explained by the higher degree of deprotonation of HCN with strong bases leading to catalytic shutdown. The effect is highest for the strong base DBU affording a mere 5% conversion to product **8**. The optimum conditions found in this work applies KOAc as base, suggesting that HCN is the reactive species and not cyanide itself.

**Fig. 1:**
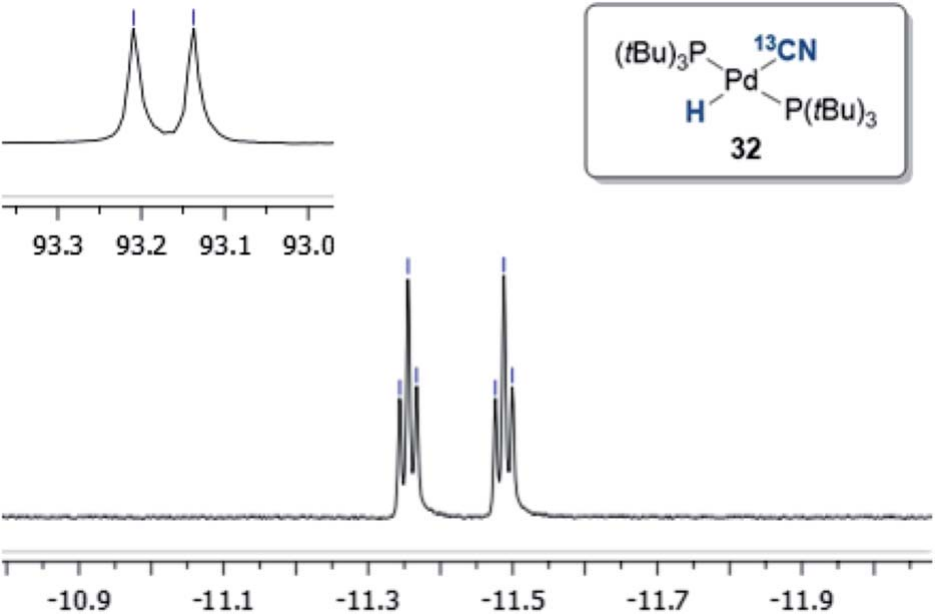
^1^H NMR (*J*_H–C_ = 53.0 Hz, *J*_H–P_ = 4.7 Hz) and ^31^P NMR (*J*_P–C_ = 11.7 Hz) of compound **32** in THF-d_8_.

**Fig. 2:**
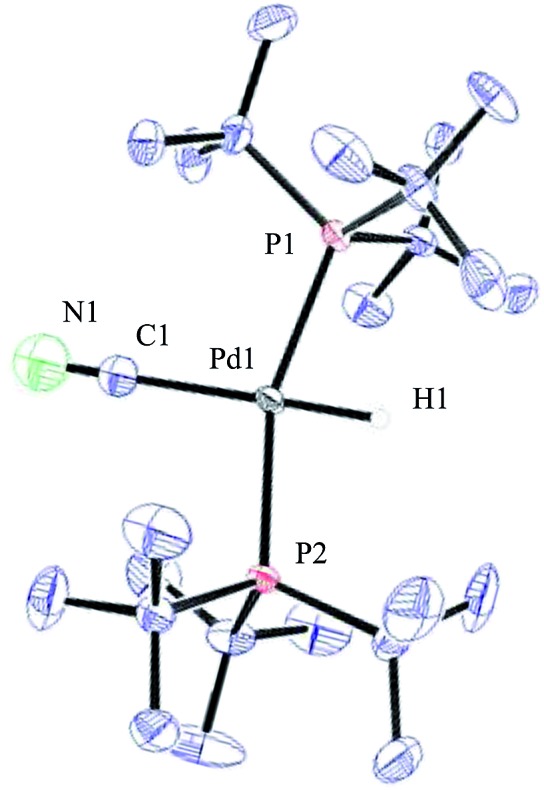
ORTEP representation of complex **32**.

**Fig. 3:**
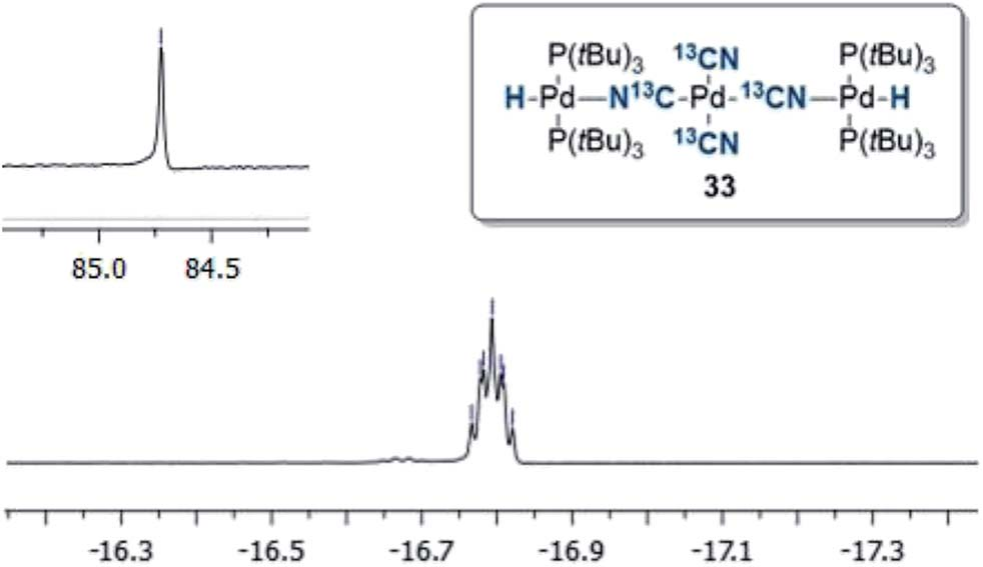
^1^H NMR and ^31^P NMR of compound **33** in CDCl_3_.

**Fig. 4:**
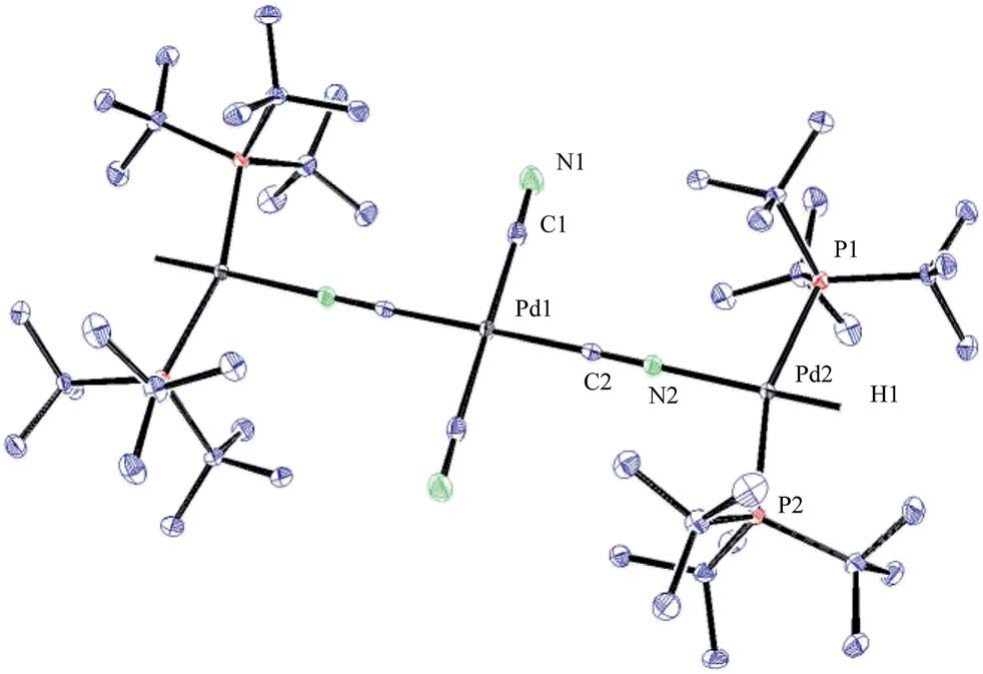
ORTEP representation of complex **33**.

Initially, the oxidative addition of Pd(P(*t*Bu)_3_)_2_ to H^13^CN in THF as the solvent was investigated using the two-chamber reactor (see ESI† for reaction details).§
§H^13^CN was used because this isotope eased the spectroscopic analysis of the complexes formed. This afforded two palladium-hydride species in roughly a 6 : 1 ratio, as observed by ^1^H NMR analysis of the reaction mixture, with the hydride signals residing at –11.42 and 16.79 ppm (Fig. 1 and 3).^35^ Careful manipulation of the reaction mixture allowed for the isolation and crystallisation of both hydride species **32** and **33**, the structures of which were confirmed by X-ray crystal structural analysis (Fig. 2 and 4).

Compound **32** was identified as the oxidative addition complex with H^13^CN, (P(*t*Bu)_3_)_2_Pd(H)(^13^CN). While others have reported the corresponding HBr and HCl adducts,^36^,^37^ it is to the best of our knowledge the first time compound **32** has been isolated. The hydride signal is located at –11.42 ppm as a double triplet in the ^1^H NMR spectrum (Fig. 1), whereby the multiplicity originates from a large *trans*-coupling with the 13-carbon of the cyanide, and small *cis*-coupling with the two equivalent phosphine ligands.¶
¶In the non-isotopically labelled version, the multiplicity is seen as a triplet. The ^31^P NMR analysis of **32** reveals a doublet at 93.1 ppm arising from P–C coupling with the ^13^C-labelled cyanide. Due to disturbance in the crystal structure, the hydride could not be located. However, in view of the combined data obtained from the NMR and the X-ray crystal structural analysis, we are confident that the structure of **32** is as indicated in Fig. 2.

To our surprise, the X-ray crystal structure analysis of the palladium-hydride complex **33** revealed it to be a tri-metallic species as illustrated in the ORTEP representation of Fig. 4. In the ^1^H NMR spectrum a hydride signal is located at –16.79 ppm as a multiplet (Fig. 3), whereas for the ^31^P NMR spectrum, a singlet at 84.7 ppm can be found. A closer look at this Pd_3_-complex, shows its resemblance to K_2_[Pd(^13^CN)_4_], which can also be produced from the reaction of Pd(0) with H^13^CN in turn formed from the hydrolysis of K^13^CN with water.^26^ In structure **33**, the anionic core, [Pd(^13^CN)_4_]^2–^, carries two cationic counter ions in the form of [(P(*t*Bu)_3_)Pd(H)]^+^.

To investigate the importance of the reaction conditions and influence on the formation of **32** and **33**, a series of experiments were performed the results of which are shown in Table 2. As can be seen from entries 1–3, neither the amount of H^13^CN nor the presence of water appears to influence the distribution between **32** and **33**. However, the addition of KOAc and water resulted in the exclusive formation of the Pd-hydride complex **32** (entry 4).‖
‖Complex **33** or the corresponding potassium salt (K_2_[Pd(^13^CN)_4_]) could not be detected by either ^31^P NMR or ^13^C NMR spectroscopic analysis. Increasing the H^13^CN loading from 1.0 to 3.0 equiv. provided a 92% conversion to **32**. Full conversion to **32** was obtained with 3 equiv. of H^13^CN in combination with 6 equiv. of KOAc (entry 6). Finally, direct formation of **33** from **32** can also be achieved by heating **32** in THF at 60 °C, and after 3 h, a 3 : 1 ratio between **32** and **33** is achieved. This result could be explained by the slow transmetallation between two Pd-hydride species **32** leading to the formation of (P(*t*Bu)_3_)_2_Pd(H)_2_ and (P(*t*Bu)_3_)_2_Pd(^13^CN)_2_.^35^,^38^ Whereas, the former can reductively eliminate generating Pd(0) and dihydrogen, we speculate that (P(*t*Bu)_3_)_2_Pd(^13^CN)_2_ could abstract cyanide from two Pd-hydride complexes **32** ultimately leading to the formation of the Pd_3_-complex **33**.

**Table 2:** Mechanistic evaluation of the reaction between H^13^CN and Pd(P(*t*Bu)_3_)_2_^*a*^

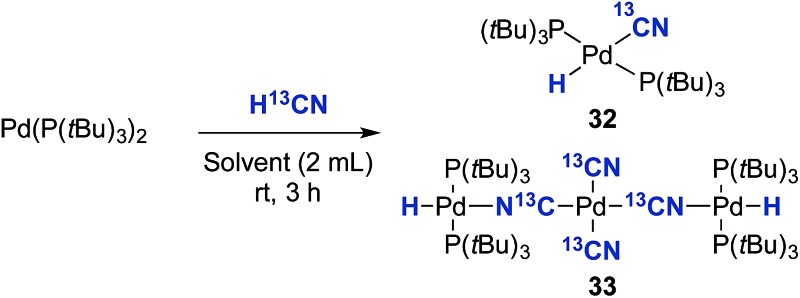				
Entry	Solvent	H^13^CN (equiv.)	KOAc (equiv.)	Ratio **32** : **33**^*b*^
1	THF	1.0	—	85 : 15 (100%)
2	THF	3.0	—	84 : 16 (100%)
3	THF : H_2_O	1.0	—	86 : 14 (100%)
4	THF : H_2_O	1.0	2.0	>95 : 5 (29%)
5	THF : H_2_O	3.0	2.0	>95 : 5 (92%)
6	THF : H_2_O	3.0	6.0	>95 : 5 (97%)

^*a*^Reactions performed on a 0.1 mmol scale. Reactions stopped after 3 h.

^*b*^Values in brackets are given as conversions.

**Scheme 6:**
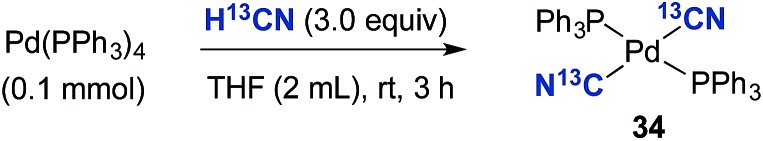
Reaction between Pd(PPh_3_)_4_ and H^13^CN. ^a^Reaction performed on a 0.1 mmol scale with a two-chamber system. The reaction was stopped after 3 h.

To further probe the necessity of the bulky and electron rich P(*t*Bu)_3_ ligand, Pd(PPh_3_)_4_ was dissolved in THF and treated with H^13^CN (Scheme 6). This resulted in the sole formation of (PPh_3_)_2_Pd(^13^CN)_2_ (**34**), the structure of which was confirmed by both NMR and X-ray analysis (see ESI†).**
**Compound **34** has previously been synthesised by Grushin and co-workers by the reaction of Pd(PPh_3_)_4_ with K^13^CN in the presence of water. See ref. 26. Possibly, the transmetallation event involving two (PPh_3_)_2_Pd(H)(^13^CN) species is significantly faster than that for complex **32**, and therefore cyanide abstraction from (PPh_3_)_2_Pd(H)(^13^CN) with complex **34** cannot compete as with similar palladium species bearing the P(*t*Bu)_3_ ligand.

**Scheme 7:**
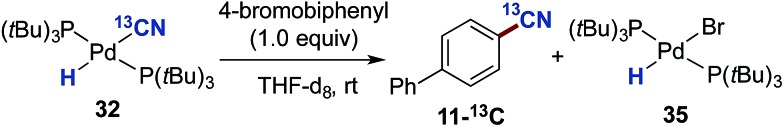
Reactivity studies between complex **32** with 4-bromo-biphenyl. ^a^0.02 mmol of both **32** and 4-bromobiphenyl were added to a NMR-tube. Mesitylene was used as an internal standard.

Next, we addressed the question whether complex **32** represents an active participant in the catalytic cycle. To this end, we examined the reaction between 4-bromobiphenyl and **32** in THF-d_8_ at room temperature with mesitylene as an internal standard (Scheme 7). The reaction progress was followed by both ^1^H- and ^31^P-NMR. From the ^1^H NMR spectrum it was clear that as the reaction progressed a build-up of the palladium-hydride species **35** in addition to the formation of the aromatic nitrile **11-^13^C** was observed. The reaction turned out to be relatively slow at 25 °C despite a 1 : 1 relationship between complex **32** and 4-bromobiphenyl. Nevertheless, after 12 h, the conversion into **11-^13^C** had reached 55%.

**Fig. 5:**
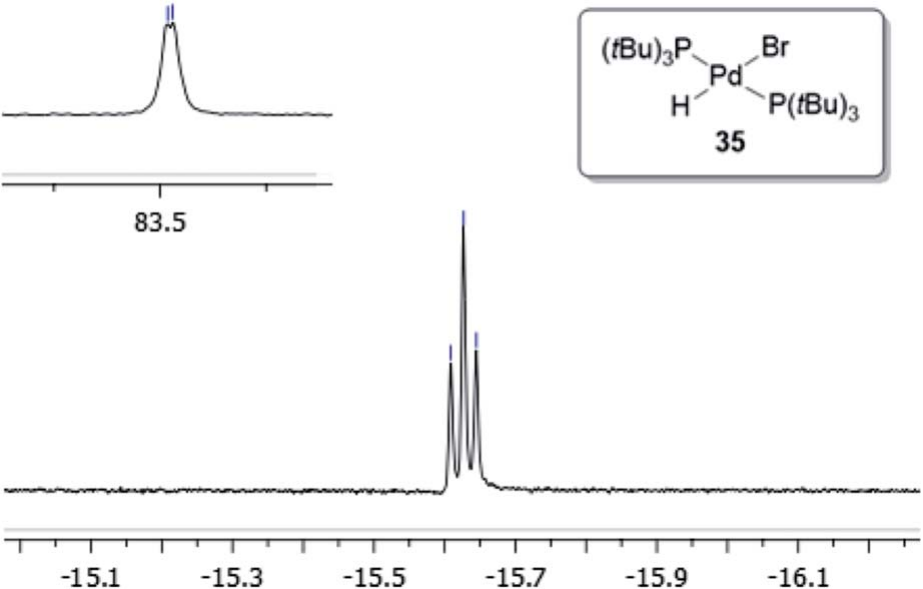
^1^H NMR (*J*_H–P_ = 7.2 Hz) and ^31^P NMR (*J* = 1.9 Hz) of compound **35** in THF-d_8_.

**Fig. 6:**
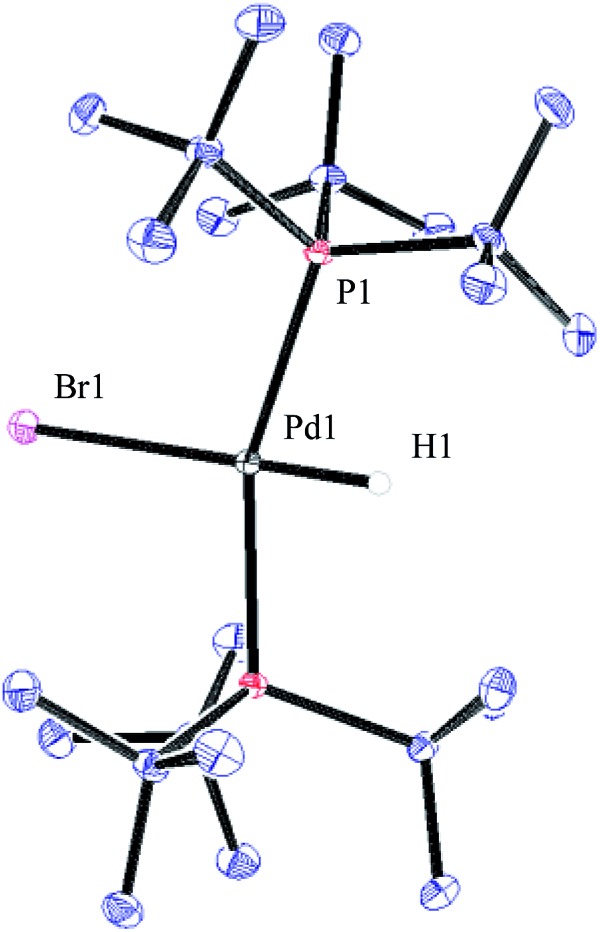
ORTEP representation of complex **35**.

To verify that the structure of the new Pd-hydride species formed indeed is as proposed for compound **35**, a sample of this complex was prepared according to a known literature procedure involving the treatment of Pd(P(*t*Bu)_3_)_2_ with pyridinium bromide in toluene.^39^ As can be seen in Fig. 5, complex **35** produced a triplet at –15.63 ppm in the ^1^H NMR spectrum arising from the coupling to the two equivalent phosphorus atoms. In the ^31^P NMR spectrum, **35** gives rise to a doublet located at 83.5 ppm. These spectroscopic data were in accord with those observed from the reaction of **32** with 4-bromobiphenyl. Finally, by dissolving **35** in a small amount of CH_2_Cl_2_ layered with heptane resulted in crystals that were suitable for X-ray analysis (Fig. 6).

The formation of aryl nitrile **11-^13^C** is only possible if complex **32** can reductively eliminate generating Pd(P(*t*Bu)_3_)_2_ and H^13^CN, thereby implying that the addition of HCN to Pd(0) is a reversible process.^40^ The Pd(0) species produced from this reductive elimination step can then either undergo an oxidative addition to H^13^CN again or alternatively to the aryl bromide generating (P(*t*Bu)_3_)Pd(Ar)(Br).^41^ With the presence in solution of both this complex and **32**, a transmetallation event could take place between these two species leading to the formation of the Pd-hydride **35** and (P(*t*Bu)_3_)Pd(Ar)(^13^CN), which subsequently undergoes reductive elimination to the desired benzonitrile **11-^13^C** and Pd(0).†
†During the reaction, no clear phosphine signals are observed from either the oxidative addition complex (*i.e.* (P(*t*Bu)_3_)Pd(Ar)(Br)) or the complex after transmetallation (*i.e.* (P(*t*Bu)_3_)Pd(Ar)(CN)). This further indicates that the rate determining step is the oxidative addition into the aryl bromide, since the generated P(*t*Bu)_3_)Pd(Ar)(Br) is consumed almost instantaneously (see J. L. Klinkenberg and J. F. Hartwig, *J. Am. Chem. Soc.*, 2012, **134**, 5748, which studies the reductive elimination of L_*n*_Pd(Ar)(CN) complexes.


**Fig. 7:**
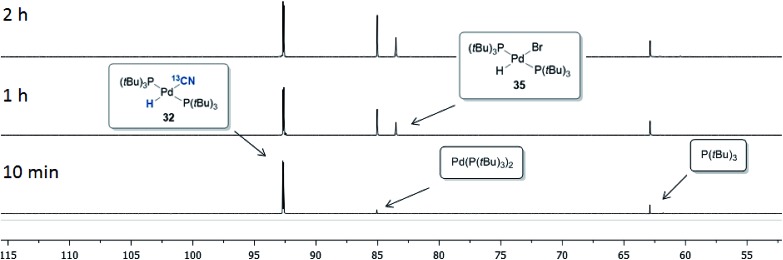
^31^P NMR spectra of the reaction between complex **32** and *p*-bromobiphenyl.

The progress of the reaction between **32** and *p*-bromobiphenyl was monitored by ^31^P NMR spectroscopy, the results of which are shown in Fig. 7. Within the first 10 minutes Pd(P(*t*Bu)_3_)_2_ (85.5 ppm) and free P(*t*Bu)_3_ (63.4 ppm) were formed, which is consistent with the observations by Fu and coworkers when a P(*t*Bu)_3_/Pd(0) ration of 2 : 1 is used.^42^ The depletion of complex **32** then occurred with concurrent build-up of the Pd-hydride **35** residing at 83.5 ppm. No signal from the oxidative addition complex (P(*t*Bu)_3_)Pd(Ar)(Br) was observed, suggesting that the oxidative addition of Pd(P(*t*Bu)_3_) to 4-bromobiphenyl is the rate-determining step in this setup.

**Scheme 8:**
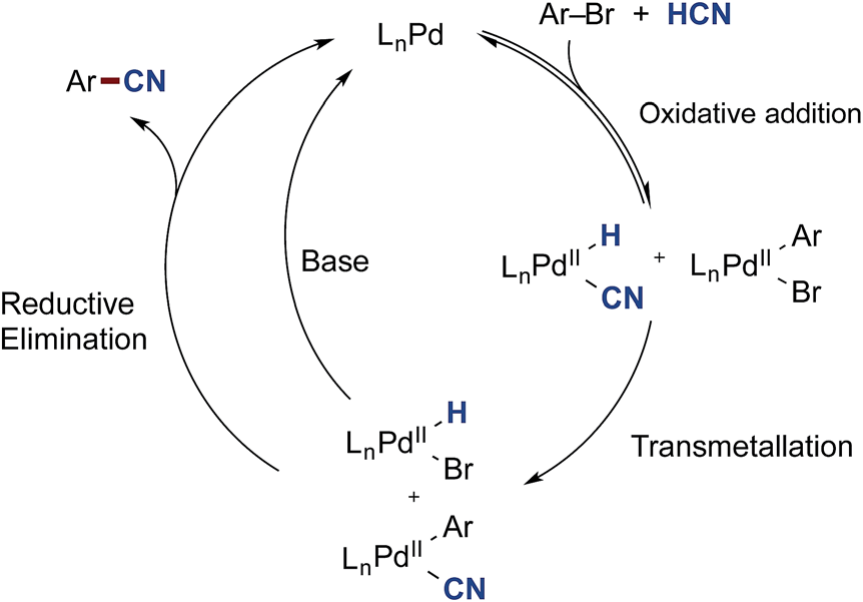
A proposed mechanism for the Pd-catalysed cyanation of aryl bromides with HCN.

From these initial experiments, a possible mechanistic scenario for the Pd-catalysed cyanation of aryl bromides with gaseous HCN is depicted in Scheme 8, which accounts for the above observations. After formation of the catalytically active palladium(0) species, two reversible oxidative addition events take place forming L_*n*_Pd(H)(CN) and L_*n*_Pd(Ar)(Br). The necessity for the oxidative addition steps to be reversible at least for the latter complex may explain why aryl triflates are not competent electrophiles for this catalytic protocol. The two complexes can then undergo transmetallation providing L_*n*_Pd(Ar)(CN) and L_*n*_Pd(H)(Br). Similar transmetallative events have previously been reported for two Pd(ii)-aryl species.^43^ Finally, the benzonitrile and the active L_*n*_Pd(0) species are reformed through two reductive elimination events involving both L_*n*_Pd(Ar)(CN) and L_*n*_Pd(H)(Br). In the latter case, base is required to initiate the regeneration of L_*n*_Pd(0), and the presence of aqueous KOAc should be sufficient for promoting this reduction step at the metal centre. Whereas stronger bases would be able to carry out this step as well, they would also lead to the deprotonation of HCN resulting in increased concentrations of cyanide anion in solution. Subsequently, this cyanide would trap the active species in the catalytic cycle as multi-cyano palladium complexes and as such lead to catalytic shutdown.

The mechanism depicted in Scheme 8 also supports the findings made by Buchwald and Hooker.^21^,^22^ In their setup, H^11^CN is introduced to a large excess of the oxidative addition complex of the same type as **36**. Only a small portion of this Ar–Pd(ii) complex would have to undergo reductive elimination to afford a Pd(0) species that could trap the added H^11^CN leading to product formation.

**Scheme 9:**
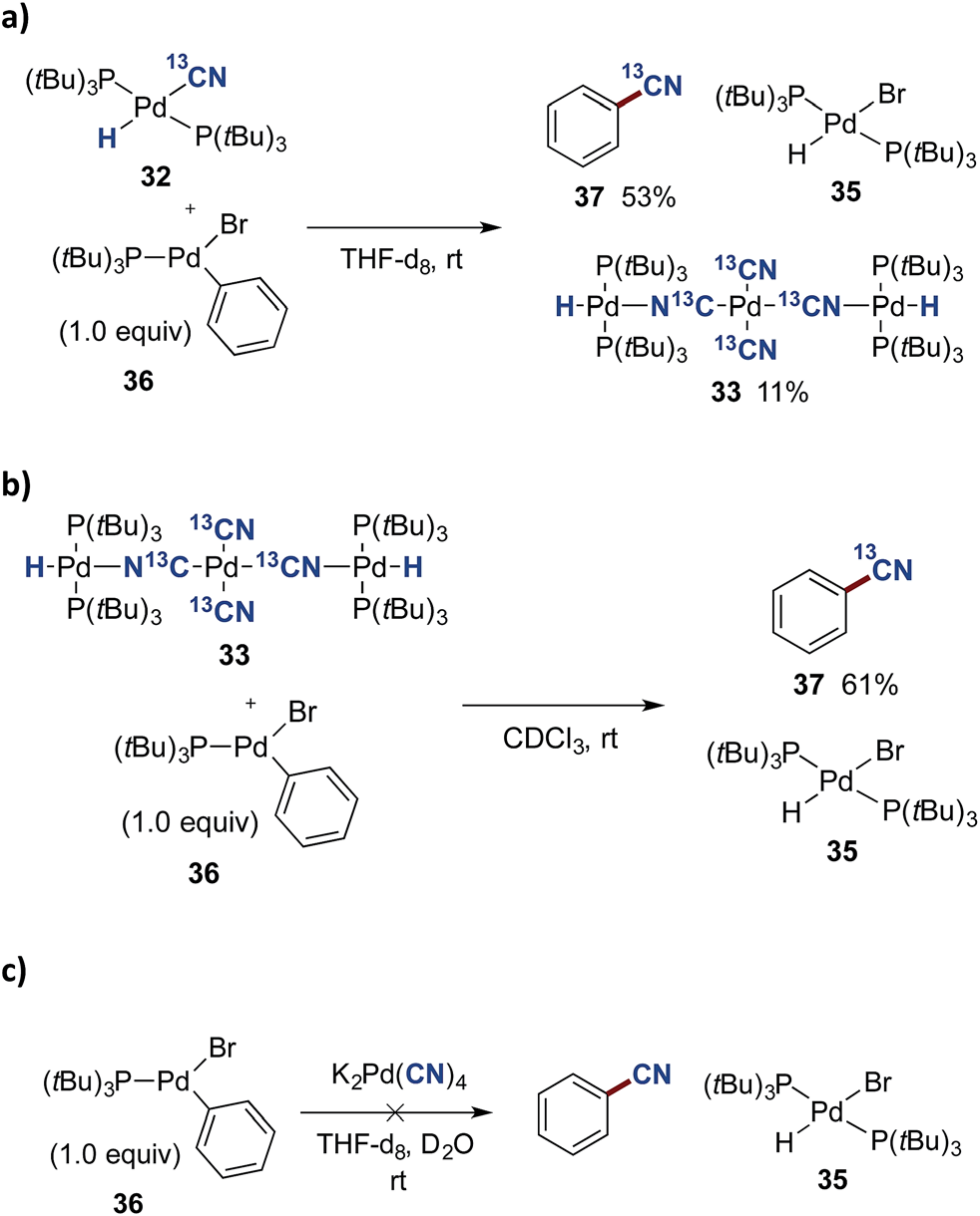
Transmetallation studies with complex **36**. ^a^0.02 mmol of either **32**, **33** or K_2_[Pd(CN)_4_] with **36** were added to a NMR-tube. Mesitylene was used as an internal standard.

In order to investigate the viability of such a transmetallation step between two Pd(ii) species of the proposed catalytic cycle in Scheme 8, we investigated the reaction between Pd-hydride **32** and the preformed oxidative addition complex **36** (Scheme 9a). The aryl–Pd complex **36** was prepared in a 79% isolated yield *via* the oxidative addition of Pd(P(*t*Bu)_3_)_2_ to bromobenzene according to a literature procedure.^44^ Complexes **32** and **36** were dissolved in THF-d_8_, and using mesitylene as the internal standard, their transformation was followed by ^31^P NMR spectroscopy. From this experiment, three species were formed as the reaction progressed, being ^13^C-benzonitrile (**37**), (P(*t*Bu)_3_)_2_Pd(H)(Br) (**35**) and the Pd_3_-complex **33**. The transformation was completed in less than 30 min, and applying the internal standard, **37** was formed in a 53% yield while most of the remaining ^13^C-labelled cyanide could be accounted for from the formation of **33** (11% yield).

Given the undoubtedly strong binding mode of cyanide to palladium(ii), the possibility of free cyanide in solution is most likely absent, thereby eliminating the possibility for the formation of **35** through a nucleophilic displacement pathway. This in turn indicates that **35** forms as a result of a transmetallation step between **36** and one or both of complexes **32** and **33**.

As mentioned above, the Pd_3_-complex **33** is presumably not formed under the optimised conditions. Nevertheless, we investigated whether **33** could also promote benzonitrile formation alone. The trinuclear complex **33** was mixed with **36** in a 1 : 1 relationship in CDCl_3_, and followed by ^1^H NMR spectroscopy. Surprisingly, complete formation of ^13^C-benzonitrile (**37**) and Pd-hydride **35** was achieved after only 2 h (Scheme 9b). To evaluate the importance of the [(P(*t*Bu)_3_)_2_Pd(H)]^+^ cation in **33**, the direct reaction between K_2_[Pd(CN)_4_] and **36** was tested (Scheme 9c). However, following the reaction by ^1^H NMR spectroscopy, no conversion was observed between these two palladium complexes as seen from the absence of signals for benzonitrile and Pd-hydride **35**.

**Scheme 10:**
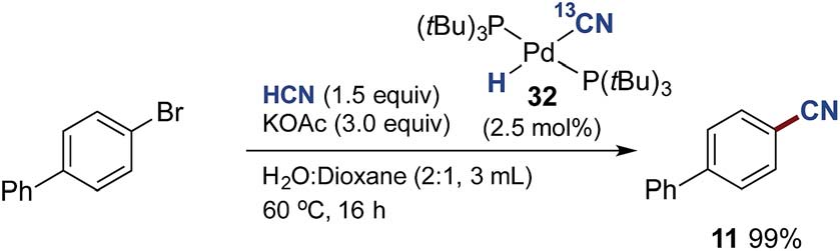
Pd-Catalysed cyanation of aryl bromides using **32** instead of P(*t*Bu_3_)-Pd-G3. ^a^Chamber A: 4-bromobiphenyl (1.0 mmol), **33** (2.5 mol%) and KOAc (3.0 mmol) in dioxane (1.0 mL) and H_2_O (2.0 mL). Chamber B: KCN (1.5 mmol), ethylene glycol (1.0 mL) and AcOH (9.0 mmol).

Next, we examined whether the different Pd-cyanide species are catalytically competent species and can promote the catalytic conversion of aryl bromides to aryl nitriles with HCN. By using **32** as the palladium source we could isolate biphenyl nitrile **11** in quantitative yield (Scheme 10). On the other hand, with **33** as the palladium source no conversion to product was observed. This is in perfect accordance with the observations shown in Table 2, demonstrating that the presence of aqueous KOAc prevents formation of Pd_3_-complex **33** and that K_2_[Pd(CN)_4_], which is most likely produced from **33** under these conditions, is inactive as a catalyst precursor.‡
‡K_2_[Pd(CN)_4_] was also tested. This resulted in no conversion to benzonitrile **11**.


**Scheme 11:**
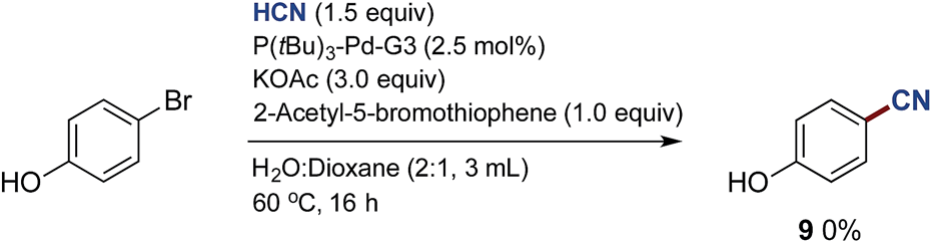
Robustness screening on the formation of benzonitrile **9**. ^a^Chamber A: 4-bromophenol (1.0 mmol), 2-acetyl-5-bromothiophene (1.0 mmol) P(*t*Bu)_3_-Pd-G3 (2.5 mol%) and KOAc (3.0 mmol) in dioxane (1.0 mL) and H_2_O (2.0 mL). Chamber B: KCN (1.5 mmol), ethylene glycol (1.0 mL) and AcOH (9.0 mmol).

**Scheme 12:**
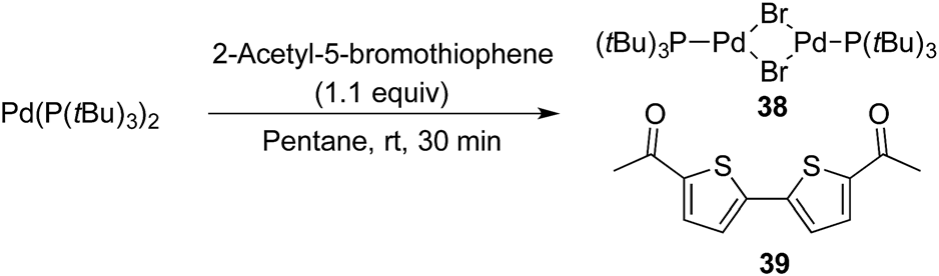
Formation of Pd_2_(μ-Br)_2_(P(*t*Bu)_3_)_2_ and 5,5′-diacetyl-2,2′-bithiophene from Pd(P(*t*Bu)_3_)_2_. ^a^Reaction performed on a 0.1 mmol scale.

Finally, while the substrate scope of the Pd-catalysed cyanation of aryl bromides using HCN proved broad, some heteroaryl bromides provided low or even no conversion to product. One of these inactive bromides, 2-acetyl-5-bromothiophenyl bromide, was added to the cyanation reaction of 4-bromophenol, which under our standard condition with HCN and in the absence of the heteroaryl bromide provided the corresponding 4-cyanophenol in high yield as depicted in Scheme 3.^45^§
§Lowering the amount of 2-acetyl-5-bromothiophenyl bromide to 0.5 equivalents or using other heteroaromatic bromides, such as 5-bromopyridine, gave the same outcome. However, under the exact same conditions but with the addition of this inactive heteroaryl bromide, the formation of benzonitrile **9** was completely inhibited (Scheme 11). Next, we attempted to isolate the oxidative addition complex of Pd(P(*t*Bu)_3_)_2_ and 2-acetyl-5-bromothiophene in order to test the stoichiometric reaction with Pd-hydride **32**. Despite extensive experimentation, *i.e.* different solvents and temperatures, we only observed the formation of Pd_2_(μ-Br)_2_(P(*t*Bu)_3_)_2_ (**38**) and 5,5′-diacetyl-2,2′-bithiophene (**39**) in all cases (Scheme 12).

The Pd-complex **38** has previously been synthesised by Hartwig and co-workers by a similar protocol using Pd(dba)_2_, P(*t*Bu)_3_ and a tenfold excess of 2-bromothiophene.^46^ The formation of **38** is fast at 25 °C when using either THF or pentane. Given that the developed conditions for the cyanation of aryl bromides operate at 60 °C, production of **38** is fast leading to apparent shutdown of the catalytic system. The formation of complex **38** offers an explanation to why some heteroaromatic bromides fail under the developed cyanation protocol, however further investigations are needed to understand the reasons for this divergence.^47^,^48^

## Conclusions

In summary, a new protocol for the direct use of stoichiometric gaseous hydrogen cyanide in the Pd-catalysed cyanation of aryl bromides has been developed. Contrary to previous studies, the use of HCN did not lead to catalytic shutdown, but instead provided a robust and reproducible method. A broad range of aryl bromides and a few heteroaromatic bromides afforded the desired benzonitrile derivatives and given the simple setup, utilising *ex situ* generation of HCN, labelling with H^13^CN was also straightforward. The presence of water as co-solvent and the use of the mild base KOAc proved imperative for catalytic efficiency. In particular, the suitability of this weak base indicated that possibly the mechanism in operation deviates from previous catalytic cyanation studies as the concentration of free cyanide would be virtually non-existent. This led to the proposal of a mechanism based on a transmetallation between two Pd-complexes produced from the oxidative addition of Pd(0) into hydrogen cyanide and an aryl bromide. This proposal was based on mechanistic indications that co-aligns with observations reported by Grushin, Beller and others. Further work is now on-going to examine a similar protocol under nickel catalysis, as well as examining other electrophiles than aryl halides. The results of this work will be reported in due course.

## Conflicts of interest

Troels Skrydstrup and Anders T. Lindhardt are co-owners of SyTracks a/s, which commercialises the two-chamber technology.

## Supplementary Material

Supplementary information

Crystal structure data
