# Supplementary material for: Ex situ generation of stoichiometric HCN and its application in the Pd-catalysed cyanation of aryl bromides: evidence for a transmetallation step between two oxidative addition Pd-complexes
Source: Chem Sci. 2017 Oct 6;8(12):8094–105. doi: 10.1039/c7sc03912c (PMC5855124; doi:10.1039/c7sc03912c)
Supplement: Supplementary file 1 [file SC-008-C7SC03912C-s001.pdf]

Electronic Supplementary Material (ESI) for Chemical Science.
This journal is © The Royal Society of Chemistry 2017

Supporting Information

Ex situ generation of stoichiometric HCN and its application in the Pd-catalysed cyanation of aryl bromides: Evidence for a transmetallation step between two oxidative addition Pd-complexes

Steffan K. Kristensen, Espen Z. Eikeland, Esben Taarning, Anders T. Lindhardt* and Troels Skrydstrup*

Table of content

General methods S3

Handling of hydrogen cyanide S3

Optimisation of the Ni-catalysed hydrocyanation of olefins S4

General procedure for Scheme 2: Ni-catalysed hydrocyanations of olefins S6

Optimisation of the Pd-catalysed cyanation of aryl bromides S9

General procedure for Scheme 3: Pd-catalysed cyanation of aryl bromides S13

Starting materials and procedures for Scheme 4 S20

Mechanistic Investigations S23

Isolation of 32 and 33 S23

Procedure for Table 2 S24

Procedure for Scheme 6 S24

Procedure for Scheme 7 S25

Formation of 35 S27

Formation of 36 S27

Procedure for Scheme 9a S28

Procedure for Scheme 9b S29

Procedure for Scheme 9c S31

Procedure for Scheme 10 S31

Procedure for Scheme 11 S31

Procedure for Scheme 12 S32

Crystallographic details S33

NMR Spectra S34

General methods

Unless otherwise stated, all reactions were performed in the commercial available COware two-chamber system, which were sealed with PTFE H-Caps.[1] All chemicals were purchased from Sigma-Aldrich and were used without further purification. Solvents were collected from a MBRAUN MB SP-800 purification system, further dried over 3Å molecular sieves and degassed by purging with argon. Analytical thin layer chromatography (TLC) was performed using silica coated aluminum plates (Merck Kieselgel 60 F_{254}), which were visualized under UV and/or stained with $KMnO_4$ (1.5 g $KMnO_4$ in 1.25 mL NaOH and 200 mL H_2O). Flash column chromatography was performed on silica gel (230-400 mesh). NMR experiments were acquired on a Bruker Ascend 400 spectrometer, where ^{1}H NMR, ^{13}C NMR, ^{19}F NMR and ^{31}P NMR were recorded at 400 MHz, 101 MHz, 367 MHz and 161.9 MHz, respectively. Chemical shifts are reported in parts per million (ppm) relative to the residual solvent signal ($CDCl_3$: 7.26 ppm for ^{1}H NMR, 77.16 ppm for ^{13}C NMR. THF-d_8: 3.58- and 1.72 ppm for ^{1}H NMR, 67.21- and 25.31 ppm for ^{13}C NMR. For ^{31}P NMR an internal H_3PO_4 (0.0 ppm) standard was used as a reference. ^{19}F NMR and ^{31}P NMR were recorded ^{1}H-decoupled. The following abbreviations are used to indicate the multiplicity in NMR spectra: s, singlet; d, doublet, t, triplet; q, quartet; dd, double doublet; ddd, double double doublet; dt, double triplet; m, multiplet; bs, broad singlet; apt, apparent triplet; apsept, apparent septet. Mass spectrometry was performed on a Bruker Maxis Impact spectrometer (LC TOF, ESI).

Handling of hydrogen cyanide

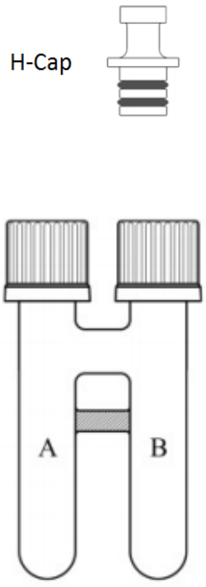

The HCN utilised in this work was produced in the two-chamber system (depicted to the right), which was sealed with PTFE H-Caps. **CAUTION!** Due to the high toxicity and low boiling point (b.p. 27 °C) of HCN, it is highly recommended that the organic waste is kept basic to quench leftover HCN. Furthermore, it is highly recommended that the chemistry is only performed in a well-ventilated fume hood.

[1] See www.Sytracks.com for a detailed description of the COWare glassware.

Optimisation of the Ni-catalysed hydrocyanation of olefins

Table S1: Ligand screening[a]

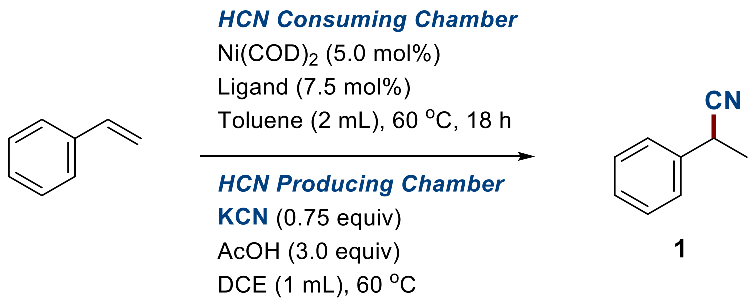

Entry	Ligand	Incorporation of HCN[b]
I	dppe	Trace
II	dppp	65%
III	dppb	54%
IV	dppf	58%
V	XantPhos	78%
VI	Si-XantPhos	41%
VII	*t*Bu-XantPhos	Trace

[a]Reactions performed using 1.0 mmol of styrene. [b]Incorporation of HCN given as a ^{1}H NMR yield using mesitylene as an internal standard.

Table S2: Ligand loading[a]

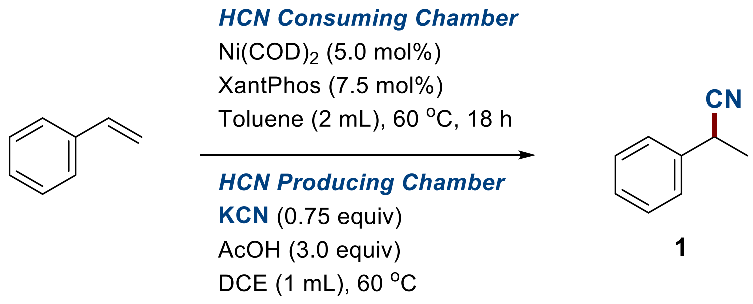

Entry	XantPhos (mol%)	Incorporation of HCN[b]
I	5.0	44%
II	6.25	52%
III	7.5	78%
IV	10.0	78%

[a]Reactions performed using 1.0 mmol of styrene. [b]Incorporation of HCN given as a ^{1}H NMR yield using mesitylene as an internal standard.

Table S3: KCN amount[a]

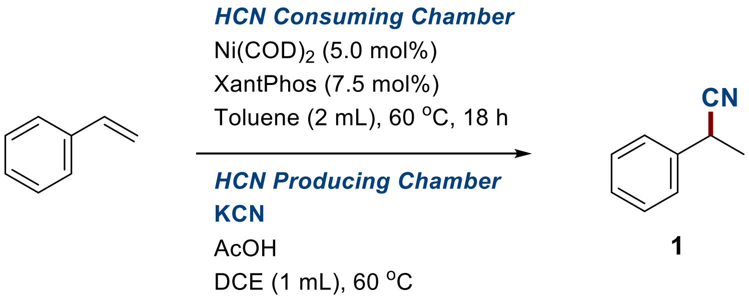

Entry	KCN (equiv)	Conversion[b]
I	1	54%
II	1.1	59%
III	1.25	65%
IV	1.5	77%
V	2.0	71%
VI	2.5	73%
VII	3.0	72%

[a]Reactions performed using 1.0 mmol of styrene. 6 equiv of AcOH compared to KCN was used. [b]Conversion given as a ^{1}H NMR yield using mesitylene as an internal standard.

At this point it was realized that DCE could diffuse to the "HCN Consuming Chamber", which potentially could hamper the reaction. Adding 250 µL DCE to the reaction as depicted in **Table S3** entry IV resulted in 0% conversion to **1**. Therefore, DCE was substituted for ethylene glycol, which gave a 87% conversion to product (see **Table S4**, entry VII).

Table S4: Solvent screening[a]

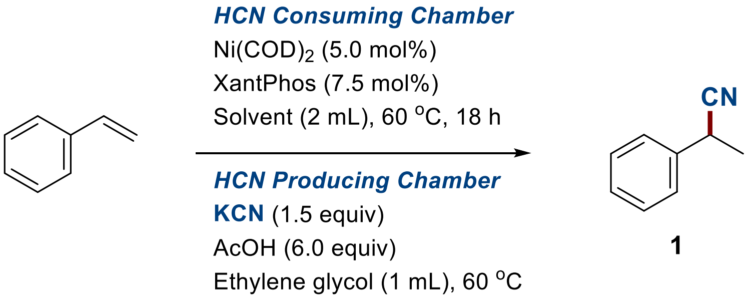

Entry	Solvent	Conversion[b]
I	THF	54%
II	MeOH	63%
III	Dioxane	58%
IV	Acetonitrile	69%
V	Anisole	79%
VI	CPME	92%
VII	Toluene	87%

[a]Reactions performed using 1.0 mmol of styrene. [b]Incorporation of HCN given as a ^{1}H NMR yield using mesitylene as an internal standard.

General procedure for Scheme 2: Ni-catalysed hydrocyanation of olefins

In an argon filled glovebox Ni(COD)$_2$ (13.8 mg, 0.05 mmol) and XantPhos (43.4 mg, 0.075 mmol) were premixed in CPME (1 mL) in a 4 mL dry vial for 20 min. HCN consuming chamber (A): To chamber A of the two-chamber system olefin (1.0 mmol) and the premixed Ni-complex were added. The total volume was increased to 2 mL of CPME. The chamber was sealed with a PTFE H-Cap and a screw cap. The HCN producing chamber (B, 1.5 equiv HCN): To chamber B of the two-chamber system KCN (97.7 mg, 1.5 mmol) and ethylene glycol (1 mL) were added. Lastly, AcOH (0.52 mL, 9 equiv) was carefully added on top of the ethylene glycol layer. The chamber was sealed with a PTFE H-Cap and a screw cap. Outside the glovebox the reaction was heated to 60 °C. After 18 hours the reaction was cooled to rt before the content of chamber A was filtered through a small Celite plug. The crude was concentrated onto Celite. Purification by flash column chromatography on silica gel using an appropriate eluent afforded the desired hydrocyanated product.

2-Phenylpropanenitrile (1)

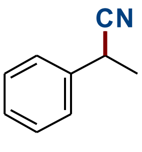

Prepared according to the general procedure in Scheme 2 using styrene (0.115 mL, 1.0 mmol). Product was purified by flash column chromatography using pentane/CH_2Cl_2 (6:1) as eluent which afforded **1** as a colorless oil (**Run 1**: 119.8 mg, 91%. **Run 2:** 122.1 mg, 93%). **^{1}H NMR (400 MHz, $CDCl_3$)**: δ 7.41-7.33 (m, 5H), 3.90 (q, *J* = 7.2 Hz, 1H), 1.65 (d, *J* = 7.2 Hz, 3H). **^{13}C NMR (101 MHz, $CDCl_3$)**: δ 137.1, 129.2 (2), 128.1, 126.7 (2), 121.6, 31.3, 21.5. **HRMS** (ESI+) *m/z* calcd. for C_9H_9N $[M+H]^+$: 132.0808; found: 132.0814.

2-(Biphenyl-4-yl)propanenitrile (2)

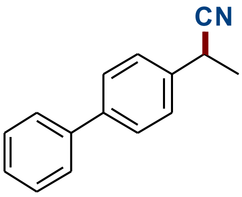

Prepared according to the general procedure in Scheme 2 using 4-vinylbiphenyl (180.3 mg, 1.0 mmol). Product was purified by flash column chromatography using pentane/acetone (20:1) as eluent which afforded **2** as a off-white solid (**Run 1**: 184.8 mg, 89%. **Run 2:** 188.2 mg, 91%). **^{1}H NMR (400 MHz, $CDCl_3$)**: δ 7.62-7.57 (m, 4H), 7.47-7.44 (m, 4H), 7.39-7.35 (m, 1H), 3.95 (q, *J* = 7.2 Hz, 1H), 1.69 (d, *J* = 7.2 Hz, 3H). **^{13}C NMR (101 MHz, $CDCl_3$)**: δ 141.3, 140.4, 136.1, 129.0 (2), 128.0 (2), 127.8, 127.3 (2), 127.2 (2), 121.7, 31.1, 21.6. **HRMS** (ESI+) *m/z* calcd. for $C_{15}H_{13}N$ $[M+Na]^+$: 230.0940; found: 230.0944.

2-(*m*-Tolyl)propanenitrile (3)

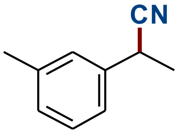

Prepared according to the general procedure in Scheme 2 using 3-methylstyrene (0.115 mL, 1.0 mmol). Product was purified by flash column chromatography using pentane/CH_2Cl_2 (6:1) as eluent which afforded **3** as a colorless oil (**Run 1**: 130.0 mg, 90%. **Run 2:** 139.8 mg, 96%). **^{1}H NMR (400 MHz, $CDCl_3$)**: δ 7.28 (t, *J* = 7.6 Hz, 1H), 7.19 (s, 1H), 7.16-7.14 (m, 2H), 3.87 (q, *J* = 7.2 Hz, 1H), 2.38 (s, 3H), 1.63 (d, *J* = 7.2 Hz, 3H). **^{13}C NMR (101 MHz, $CDCl_3$)**: δ 139.0, 137.1, 129.0, 128.8, 127.4, 123.8, 121.8, 31.2, 21.5, 21.4. **HRMS** (ESI+) *m/z* calcd. for $C_{10}H_{11}N$ $[M+H]^+$: 146.0964; found: 146.0955.

2-(3,4-Dimethoxyphenyl)propanenitrile (4)

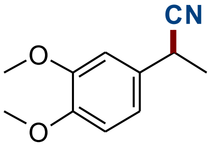

Prepared according to the general procedure in Scheme 2 using 3,4-dimethoxystyrene (0.133 mL, 1.0 mmol). Product was purified by flash column chromatography using pentane/EtOAc (15:1 to 4:1 gradient) as eluent which afforded **4** as a colorless oil (**Run 1**: 187.1 mg, 98%. **Run 2:** 175.5 mg, 92%). **^{1}H NMR (400 MHz, $CDCl_3$)**: δ 6.90-6.84 (m, 3H), 3.90 (s, 3H), 3.87 (s, 3H), 3.85 (q, *J* = 7.4 Hz, 1H), 1.63 (d, *J* = 7.4 Hz, 3H). **^{13}C NMR (101 MHz, $CDCl_3$)**: δ 149.5, 148.9, 129.6, 121.9, 119.0, 111.6, 109.9, 56.1 (2), 31.0, 21.6. **HRMS** (ESI+) *m/z* calcd. for $C_{11}H_{13}NO_2$ $[M+H]^+$: 192.1019; found: 192.1018.

2-(4-Fluorophenyl)propanenitrile (5)

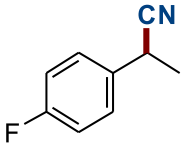

Prepared according to the general procedure in Scheme 2 using 4-fluorostyrene (0.119 mL, 1.0 mmol). Product was purified by flash column chromatography using pentane/CH_2Cl_2 (6:1) as eluent which afforded **5** as a colorless oil (**Run 1**: 144.1 mg, 97%. **Run 2:** 138.2 mg, 93%). **^{1}H NMR (400 MHz, $CDCl_3$)**: δ 7.35-7.31 (m, 2H), 7.10-7.05 (m, 2H), 3.89 (q, *J* = 7.2 Hz, 1H), 1.63 (d, *J* = 7.2 Hz, 3H). **^{13}C NMR (101 MHz, $CDCl_3$)**: δ 162.5 (d, *J* = 247.3 Hz, 1C), 133.0 (d, *J* = 3.3 Hz,

1C), 128.6 (d, *J* = 8.3 Hz, 2C), 121.5, 116.3 (d, *J* = 21.8 Hz, 2C), 30.7, 21.7. **^{19}F NMR (376 MHz, $CDCl_3$)**: δ 113.9 **HRMS** (ESI+) *m/z* calcd. for C_9H_8FN $[M+H]^+$: 150.0714; found: 150.0725.

2-(4-Acetoxyphenyl)propanenitrile (6)

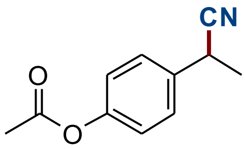

Prepared according to the general procedure in Scheme 2 using 4-acetoxystyrene (162.2 mg, 1.0 mmol). Product was purified by flash column chromatography using pentane/acetone (10:1) as eluent which afforded **6** as a colorless solid (**Run 1**: 171.8 mg, 91%. **Run 2:** 172.1 mg, 91%). **^{1}H NMR (400 MHz, $CDCl_3$)**: δ 7.37 (d, *J* = 8.4 Hz, 2H), 7.12 (d, *J* = 8.4 Hz, 2H), 3.91 (q, *J* = 7.2 Hz, 1H), 2.31 (s, 3H), 1.65 (d, *J* = 7.2 Hz, 3H). **^{13}C NMR (101 MHz, $CDCl_3$)**: δ 169.5, 150.5, 134.7, 128.0 (2), 122.5 (2), 121.5, 30.9, 21.6, 21.2. **HRMS** (ESI+) *m/z* calcd. for $C_{11}H_{11}NO_2$ $[M+H]^+$: 190.0863; found: 190.0864.

4-(1-Cyanoethyl)benzoic acid (7)

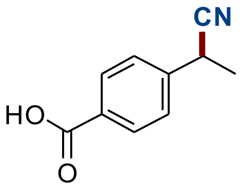

Prepared according to the general procedure in Scheme 2 using 4-vinylbenzoic acid (148.2 mg, 1.0 mmol). After filtration the crude mixture was diluted with CH_2Cl_2 (10 mL) and Na_2CO_3 (sat. aq., 15 mL) was added. The aqueous phase was washed with CH_2Cl_2 (3x10 mL), acidified with 4M HCl (aq.) and extracted with CH_2Cl_2 (5x10 mL). The organic phase was concentrated under reduced pressure, dried over $MgSO_4$, filtered and evaporated onto Celite. Purification by flash column chromatography using CH_2Cl_2/EtOAc (2:1) as eluent afforded **7** as a colorless solid (**Run 1**: 160.4 mg, 92%. **Run 2:** 161.5 mg, 92%). **^{1}H NMR (400 MHz, $CDCl_3$)**: δ 11.73 (bs, 1H), 8.15 (d, *J* = 8.3 Hz, 2H), 7.49 (d, *J* = 8.3 Hz, 2H), 3.99 (q, *J* = 7.3 Hz, 1H), 1.69 (d, *J* = 7.3 Hz, 3H). **^{13}C NMR (101 MHz, $CDCl_3$)**: δ 171.0, 143.0, 131.3 (2), 129.3, 127.1 (2), 120.9, 31.5, 21.4. **HRMS** (ESI+) *m/z* calcd. for $C_{10}H_9NO_2$ $[M+H]^+$: 176.0706; found: 176.0706.

Optimisation of the Pd-catalysed cyanation of aryl bromides

Table S5: Ligand screening[a]

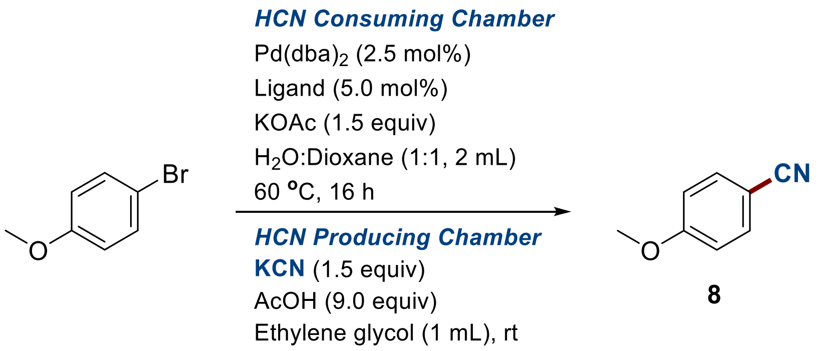

Entry	ligand	Conversion[b]
I	PCy_3	0%
II	CataCXium A	0%
III	PPh_3	0%
IV	P(*t*Bu)$_3$	78%
V	XPhos	60%
VI	*t*Bu-XPhos	74%
VII	XantPhos (2.5 mol%)	0%
VIII	dppp (2.5 mol%)	0%
IX	dppf (2.5 mol%)	0%

[a]Reactions performed using 1.0 mmol of 4-bromoanisole. [b]Conversion given as a ^{1}H NMR yield using mesitylene as an internal standard.

Table S6: Control experiments[a]

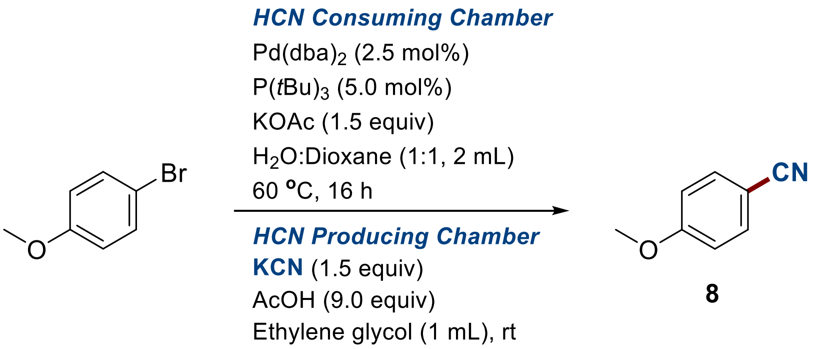

Entry	Deviations	Conversion[b]
I	No Pd or ligand	0%
II	No Base	0%
III	Only Dioxane	0%

[a]Reactions performed using 1.0 mmol of 4-bromoanisole. [b]Conversion given as a ^1H NMR yield using mesitylene as an internal standard.

Table S7: Base Screening[a]

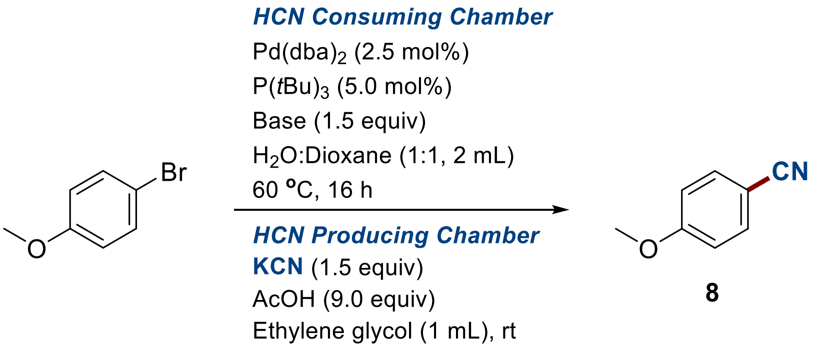

Entry	Base	Conversion[a]
I	KOAc	78%
II	NaOAc	82%
III	KO-*t*Bu	60%
IV	Cs_2CO_3	42%
V	K_3PO_4	30%
VI	Cy_2NMe	38%
VII	DBU	5%
VIII	Et_3N	67%

[a]Reactions performed using 1.0 mmol of 4-bromoanisole. [b]Conversion given as a ^1H NMR yield using mesitylene as an internal standard.

NaOAc was abandoned, since KOAc came as a fine powder, while NaOAc had to be grinded prior to use.

Table S8: Base amount[a]

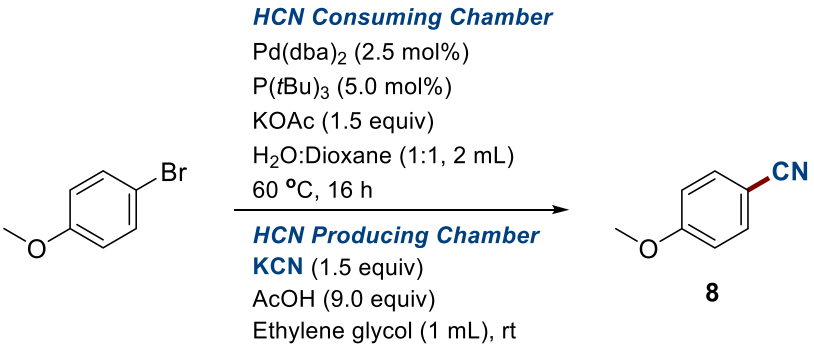

Entry	KOAc (equiv)	Conversion[b]
I	1.5	78%
II	2.0	73%
III	3.0	82%
IV	4.0	74%

[a]Reactions performed using 1.0 mmol of 4-bromoanisole. [b]Conversion given as a ^{1}H NMR yield using mesitylene as an internal standard.

Table S9: Ligand loading[a]

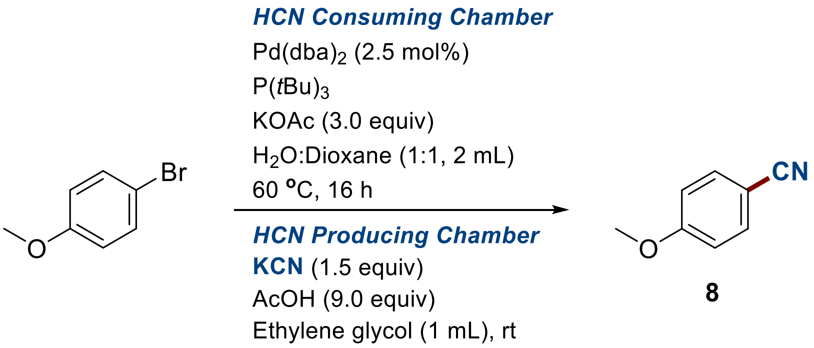

Entry	Ligand (mol%)	Conversion[b]
I	5.0	82%
II	3.75	80%
III	2.5	79%

[a]Reactions performed using 1.0 mmol of 4-bromoanisole. [b]Conversion given as a ^{1}H NMR yield using mesitylene as an internal standard.

Table S10: Pre-catalyst and evaluation of base amount[a]

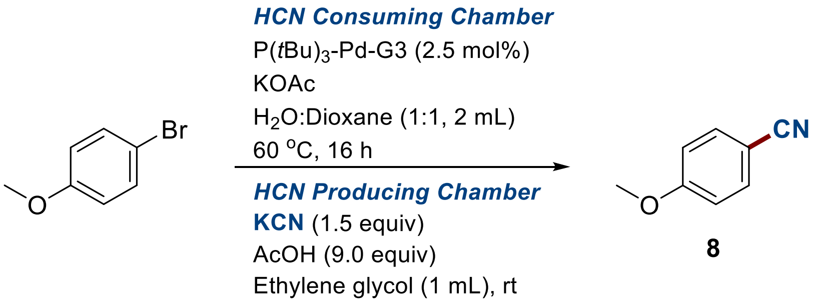

Entry	Base (equiv)	Conversion[b]
I	2.0	87%
II	3.0	90%
III	4.0	91%

[a]Reactions performed using 1.0 mmol of 4-bromoanisole. [b]Conversion given as a ^{1}H NMR yield using mesitylene as an internal standard.

Table S11: Solvent screening[a]

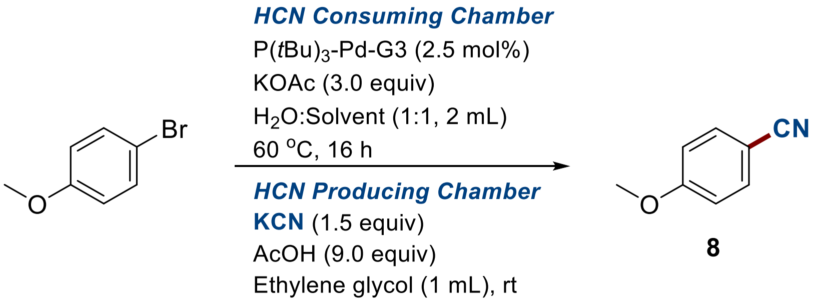

Entry	Solvent	Conversion[b]
I	Dioxane	90%
II	THF	77%
III	CH_3CN	83%
IV	CPME	85%
V	Toluene	61%
VI	Anisole	71%

[a]Reactions performed using 1.0 mmol of 4-bromoanisole. [b]Conversion given as a ^{1}H NMR yield using mesitylene as an internal standard.

Table S12: KCN loading[a]

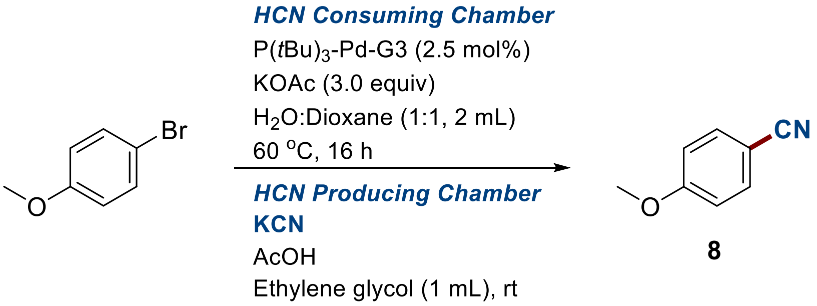

Entry	KCN (equiv)	Conversion[b]
I	1.0	69%
II	1.25	70%
III	1.5	90%
IV	1.75	92%
V	2.0	93%

[a]Reactions performed using 1.0 mmol of 4-bromoanisole. 6 equiv of AcOH compared to KCN was used [b]Conversion given as a ^{1}H NMR yield using mesitylene as an internal standard.

General procedure for Scheme 4: Pd-catalysed cyanation of aryl bromides

All reactions were set up in an argon filled glovebox. The HCN consuming chamber (A): To chamber A of the two-chamber system P(*t*Bu)$_3$-Pd-G3 (14.3 mg, 0.025 mmol), KOAc (0.295 g, 3.0 mmol), aryl bromide (1.0 mmol), dioxane (1 mL) and H_2O (2 mL) were added. The chamber was sealed with a PTFE H-Cap and a screw cap. The HCN producing chamber (B, 1.5 equiv HCN): To chamber B of the two-chamber system KCN (97.7 mg, 1.5 mmol) or K^{13}CN (99.2 mg, 1.5 mmol) and ethylene glycol (1 mL) were added. Lastly, AcOH (0.52 mL, 9 equiv) was added carefully on top of the ethylene glycol layer. The chamber was sealed with a PTFE H-Cap and a screw cap. Outside the glovebox chamber A was heated to 60 °C, while chamber B was kept at rt. After 16 hours chamber A was cooled to rt before its content was filtered through a small Celite plug. The mixture was added H_2O (5 mL) and the two phases were separated. The aqueous phase was extracted with CH_2Cl_2 (3x5 mL), and the combined organic phases were dried over $MgSO_4$, filtrated and concentrated onto Celite. Purification by flash column chromatography on silica gel using an appropriate eluent afforded the desired benzonitrile.

4-Methoxybenzonitrile (8)

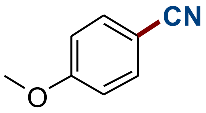

Prepared according to the general procedure in Scheme 3 using 4-bromoanisole (0.125 mL, 1.0 mmol). Product purified by flash column chromatography using pentane/Et_2O (4:1) as eluent which afforded **8** as a yellow solid (**Run 1:** 121.2 mg, 91%. **Run 2:** 120.2 mg, 90%). **^{1}H NMR (400 MHz, $CDCl_3$)**: δ 7.59 (d, *J* = 8.9 Hz, 2H), 6.95 (d, *J* = 8.9 Hz, 2H), 3.86 (s, 3H). **^{13}C NMR (101 MHz, $CDCl_3$)**: δ 163.0, 134.1 (2), 119.4, 114.9 (2), 104.1, 55.7. **HRMS** (ESI+) *m/z* calcd. for C_8H_7NO $[M+H]^+$: 134.0600; found: 134.0601.

4-Hydroxybenzonitrile (9)

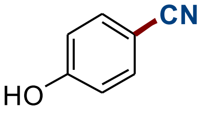

Prepared according to the general procedure in Scheme 3 using 4-bromophenol (173.0 mg, 1.0 mmol). Product was purified by flash column chromatography using pentane/acetone (5:1) as eluent which afforded **9** as a colorless solid (**Run 1**: 110.4 mg, 93%. **Run 2:** 110.0 mg, 92%). **^{1}H NMR (400 MHz, $CDCl_3$)**: δ 7.57-7.55 (m, 2H), 6.93-6.91 (m, 2H) 6.02 (bs, 1H). **^{13}C NMR (101 MHz, $CDCl_3$)**: δ 159.9, 134.5 (2), 119.3, 116.5 (2), 103.8. **HRMS** (ESI+) *m/z* calcd. for C_7H_5NO $[M+H]^+$: 142.0444; found: 142.0442.

4-(*tert*-Butyl)benzonitrile (10)

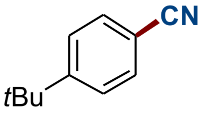

Prepared according to the general procedure in Scheme 3 using 1-bromo-4-*tert*-butylbenzene (159.2 mg, 1.0 mmol). Product was purified by flash column chromatography using pentane/Et_2O (25:1) as eluent which afforded **10** as a colorless oil (**Run 1**: 150.2 mg, 94%. **Run 2:** 143.0 mg, 90%). **^{1}H NMR (400 MHz, $CDCl_3$)**: δ 7.59 (d, *J* = 8.6 Hz, 2H), 7.48 (d, *J* = 8.6 Hz, 2H), 1.33 (s, 9H). **^{13}C NMR (101 MHz, $CDCl_3$)**: δ 156.8, 132.1 (2), 126.3 (2), 119.3, 109.5, 35.4, 31.1 (3). **HRMS** (ESI+) *m/z* calcd. for $C_{11}H_{13}N$ $[M+H]^+$: 160.1121; found: 160.1121.

4-(*tert*-Butyl)benzonitrile-(^{13}CN) (10-^{13}C)

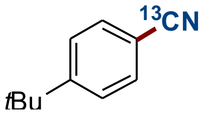

Prepared according to the general procedure in Scheme 3 using 1-bromo-4-*tert*-butylbenzene (213.1 mg, 1.0 mmol) and $K^{13}CN$ (99.2 mg, 1.5 mmol) instead of KCN. Product was purified by flash column chromatography using pentane/acetone (25:1) as eluent which afforded **10-^{13}C** as a colorless solid (147.2 mg, 92%). **^{1}H NMR (400 MHz, $CDCl_3$)**: δ 7.61-7.57 (m, 2H), 7.50-7.46 (m, 2H), 1.33 (s, 9H). **^{13}C NMR (101 MHz, $CDCl_3$)**: δ 156.8 (d, *J* = 1.5 Hz, 1C), 132.1 (d, *J* =

2.6 Hz, 2C), 126.3 (d, J = 5.5 Hz, 2C), 119.3, 109.4 (d, J = 81.9 Hz, 1C), 35.4, 31.1 (3C). **HRMS** (ESI+) m/z calcd. for $C_{10}{}^{13}CH_{13}N$ $[M+H]^+$: 161.1154; found: 161.1156.

(1,1'-Biphenyl)-4-carbonitrile (11)

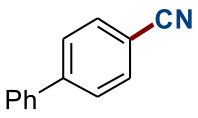

Prepared according to the general procedure in Scheme 3 using 4-bromo-1,1'-biphenyl (233.1 mg, 1.0 mmol). Product was purified by flash column chromatography using pentane/Et_2O (15:1) as eluent which afforded **11** as a colorless solid (**Run 1**: 173.9 mg, 97%. **Run 2:** 173.1, 97%). **^{1}H NMR (400 MHz, $CDCl_3$)**: δ 7.74-7.68 (m, 4H), 7.61-7.58 (m, 2H), 7.51-7.47 (m, 2H), 7.45-7.41 (m, 1H). **^{13}C NMR (101 MHz, $CDCl_3$)**: δ 145.8, 139.3, 132.7 (2), 129.3 (2), 128.8, 127.9 (2), 127.4 (2), 119.1, 111.1. **HRMS** (ESI+) m/z calcd. for $C_{13}H_9N$ $[M+H]^+$: 180.0808; found: 180.0804.

(1,1'-Biphenyl)-4-carbonitrile-(^{13}CN) (11-^{13}C)

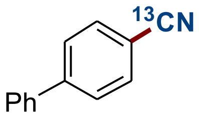

Prepared according to the general procedure in Scheme 3 using 4-bromo-1,1'-biphenyl (233.1 mg, 1.0 mmol) and $K^{13}CN$ (99.2 mg, 1.5 mmol) instead of KCN. Product was purified by flash column chromatography using pentane/Et_2O (25:1) as eluent which afforded **11-^{13}C** as a colorless solid (178.1 mg, 99%). **^{1}H NMR (400 MHz, $CDCl_3$)**: δ 7.75-7.68 (m, 4H), 7.61-7.58 (m, 2H), 7.51-7.47 (m, 2H), 7.45-7.41 (m, 1H). **^{13}C NMR (101 MHz, $CDCl_3$)**: δ 145.8 (d, J = 1.7 Hz, 1C), 139.3, 132.7 (d, J = 2.6 Hz, 2C), 129.3 (2), 128.8, 127.9 (d, J = 5.6 Hz, 2C), 127.4 (2), 119.1, 111.0 (d, J = 82.1 Hz, 1C). **HRMS** (ESI+) m/z calcd. for $C_{12}{}^{13}CH_9N$ $[M+H]^+$: 181.0841; found: 181.0842.

1,4-Dicyanobenzene (12)

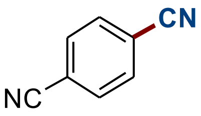

Prepared according to the general procedure in Scheme 3 using 4-bromobenzonitrile (182.0 mg, 1.0 mmol). Product was purified by flash column chromatography using pentane/acetone (7:1) as eluent which afforded **12** as a colorless solid (**Run 1**: 123.0 mg, 96%. **Run 2:** 126.9 mg, 99%).**^{1}H NMR (400 MHz, $CDCl_3$)**: δ 7.80 (s, 4H). **^{13}C NMR (101 MHz, $CDCl_3$)**: δ 132.9 (4), 117.1 (2), 116.9 (2). **HRMS** (ESI+) m/z calcd. for $C_8H_4N_2$ $[M+H]^+$: 129.0447; found: 129.0452.

1,4-Dicyanobenzene-(^{13}CN) (12-^{13}C)

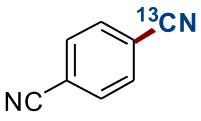

Prepared according to the general procedure in Scheme 3 using 4-bromobenzonitrile (182.0 mg, 1.0 mmol) and $K^{13}CN$ (99.2 mg, 1.5 mmol) instead of KCN. Product was purified by flash column chromatography using pentane/acetone (15:1) as eluent which

afforded **12-^{13}C** as a colorless solid (118.2 mg, 92%). **^{1}H NMR (400 MHz, $CDCl_3$)**: δ 7.80 (s, 2H), 7.79 (s, 2H). **^{13}C NMR (101 MHz, $CDCl_3$)**: δ 132.8 (d, *J* = 1.4 Hz, 2C), 132.8 (d, *J* = 1.6 Hz, 2C), 117.1, 117.0, 116.9 (d, *J* = 4.4 Hz, 1C), 116.8 (d, *J* = 1.8 Hz, 1C). **HRMS** (ESI+) *m/z* calcd. for $C_7{}^{13}CH_4N_2$ $[M+H]^+$: 130.0481; found: 130.0482.

4-Acetylbenzonitrile (13)

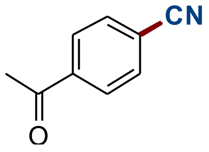

Prepared according to the general procedure in Scheme 3 using 4-bromoacetophenone (199.0 mg, 1.0 mmol). Product was purified by flash column chromatography using pentane/acetone (6:1) as eluent which afforded **13** as an yellow solid (**Run 1**: 129.8 mg, 90%. **Run 2:** 134.6 mg, 93%). **^{1}H NMR (400 MHz, $CDCl_3$)**: δ 8.04 (d, *J* = 8.6 Hz, 2H), 7.78 (d, *J* = 8.6 Hz, 2H), 2.64 (s, 3H). **^{13}C NMR (101 MHz, $CDCl_3$)**: δ 196.6, 140.1, 132.7 (2), 128.8 (2), 118.1, 116.6, 26.9. **HRMS** (ESI+) *m/z* calcd. for C_9H_7NO $[M+H]^+$: 146.0600; found: 146.0601

Ethyl 4-cyanobenzoate (14)

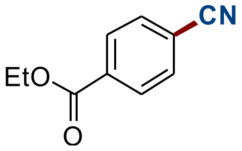

Prepared according to the general procedure in Scheme 3 using ethyl 4-bromobenzoate (0.163 mL, 1.0 mmol). Product was purified by flash column chromatography using pentane/Et_2O (5:1) as eluent which afforded **14** as a yellow solid (**Run 1**: 173.4 mg, 99%. **Run 2:** 174.2 mg, 99%). **^{1}H NMR (400 MHz, $CDCl_3$)**: δ 8.14 (d, *J* = 8.6 Hz, 2H), 7.74 (d, *J* = 8.6 Hz, 2H), 4.42 (q, *J* = 7.1 Hz, 2H), 1.41 (t, *J* =7.1 Hz, 3H) **^{13}C NMR (101 MHz, $CDCl_3$)**: δ 165.0, 134.2, 132.2 (2), 130.1 (2), 118.0, 116.3, 61.8, 14.2. **HRMS** (ESI+) *m/z* calcd. for $C_{10}H_9NO_2$ $[M+H]^+$: 176.0706; found: 176.0709.

4-Cyanobenzoic acid (15)

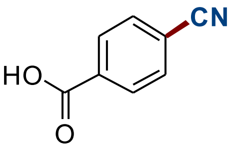

Prepared according to the general procedure in Scheme 3 using 4-bromobenzoic acid (147.1 mg, 1.0 mmol), P(*t*Bu)$_3$-Pd-G3 (28.6 mg, 0.05 mmol) and KOAc (392.6 mg, 4.0 mmol). After filtration the crude mixture was diluted with CH_2Cl_2 (10 mL) and Na_2CO_3 (sat. aq., 15 mL) were added. The aqueous phase was washed with CH_2Cl_2 (3x10 mL), acidified with 4M HCl (aq.) and extracted with CH_2Cl_2 (5x10 mL). The organic phase was concentrated under reduced pressure, dried over $MgSO_4$, filtered and evaporated onto Celite. Purification by flash column chromatography using CH_2Cl_2/EtOAc (2:1) as eluent afforded **15** as a colorless solid (**Run 1**: 127.2 mg, 86%. **Run 2:** 126.3 mg, 86%). **^{1}H NMR (400 MHz, $CDCl_3$)**: δ 8.22 (d, *J* = 8.0 Hz, 2H), 7.80 (d, *J* = 8.0 Hz, 2H) **^{13}C NMR (101 MHz, $CDCl_3$)**: δ 169.5, 133.0, 132.5 (2), 130.9 (2), 117.9, 117.5. **HRMS** (ESI+) *m/z* calcd. for $C_8H_5NO_2$ $[M+H]^+$: 148.1405; found: 148.1405.

4-Formylbenzonitrile (16)

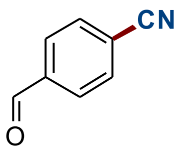

Prepared according to the general procedure in Scheme 3 using 4-bromobenzaldehyde (185.0 mg, 1.0 mmol). Product was purified by flash column chromatography using pentane/acetone (10:1) as eluent which afforded **16** as a yellow solid (**Run 1**: 113.8 mg, 87%. **Run 2:** 112.1 mg, 86%). **^{1}H NMR (400 MHz, $CDCl_3$)**: δ 10.10 (s, 1H), 8.0 (d, *J* = 8.4 Hz, 2H), 7.85 (d, *J* = 8.4 Hz, 2H). **^{13}C NMR (101 MHz, $CDCl_3$)**: δ 190.7, 138.9, 133.0 (2), 130.0 (2), 117.8, 117.8. **HRMS** (ESI+) *m/z* calcd. for C_8H_5NO [M+H]$^+$: 132.0444; found: 132.0444.

2-Aminobenzonitrile (17)

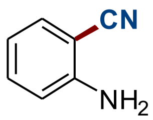

Prepared according to the general procedure in Scheme 3 using 2-bromoaniline (172.0 mg, 1.0 mmol). Product was purified by flash column chromatography using pentane/Et_2O (2:1) as eluent which afforded **17** as a off-white solid (**Run 1**: 110.8 mg, 94%. **Run 2:** 112.2 mg, 95%). **^{1}H NMR (400 MHz, $CDCl_3$)**: δ 7.39 (dd, *J* = 7.9, 1.3 Hz, 1H), 7.35-7.31 (m, 1H), 6.76-6.73 (m, 2H), 4.38 (bs, 2H). **^{13}C NMR (101 MHz, $CDCl_3$)**: δ 149.7, 134.1, 132.5, 118.2, 117.7, 115.3, 96.3. **HRMS** (ESI+) *m/z* calcd. for $C_7H_6N_2$ [M+H]$^+$: 119.0604; found: 119.0604.

3-Aminobenzonitrile (18)

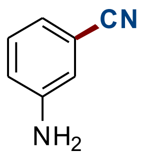

Prepared according to the general procedure in Scheme 3 using 3-bromoaniline (0.109 mL, 1.0 mmol). Product was purified by flash column chromatography using pentane/EtOAc (5.5:1) as eluent which afforded **18** as a off-white solid (**Run 1**: 76.9 mg, 65%. **Run 2:** 104.4 mg, 88%). **^{1}H NMR (400 MHz, $CDCl_3$)**: δ 7.21 (apt, *J* = 7.9 Hz, 1H), 7.01 (dt, *J* = 7.9, 1.1 Hz, 1H), 6.90 (apt, *J* = 2.0, 1H), 6.86 (ddd, *J* = 7.9, 2.0, 1.1, 1H), 3.88 (bs, 2H). **^{13}C NMR (101 MHz, $CDCl_3$)**: δ 147.1, 130.2, 122.1 (2), 119.3, 117.6, 113.1. **HRMS** (ESI+) *m/z* calcd. for $C_7H_6N_2$ [M+H]$^+$: 119.0604; found: 119.0604.

4-Aminobenzonitrile (19)

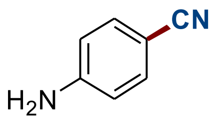

Prepared according to the general procedure in Scheme 3 using 4-bromoaniline (172.0 mg, 1.0 mmol). Product was purified by flash column chromatography using pentane/EtOAc (5.5:1) as eluent which afforded **19** as a colorless solid (**Run 1**: 53.8 mg, 46%. **Run 2:** 58.1mg, 49%). **^{1}H NMR (400 MHz, $CDCl_3$)**: δ 7.40 (d, *J* = 8.7 Hz, 2H), 6.64 (d, *J* = 8.7 Hz, 2H), 4.17 (bs, 2H). **^{13}C NMR (101 MHz, $CDCl_3$)**: δ 150.5, 133.9 (2), 120.2, 114.6 (2), 100.3. **HRMS** (ESI+) *m/z* calcd. for $C_7H_6N_2$ [M+H]$^+$: 119.0604; found: 119.0604.

2,4-Dimethylbenzonitrile (20)

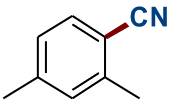

Prepared according to the general procedure in Scheme 3 using 2-bromo-2,4-dimethylbenzene (0.135 mL, 1.0 mmol). Product was purified by flash column chromatography using pentane/Et_2O (10:1) as eluent which afforded **20** as a colorless oil (**Run 1**: 123.1 mg, 93%. **Run 2:** 118.3 mg, 90%). **^{1}H NMR (400 MHz, $CDCl_3$)**: δ 7.50 (d, *J* = 7.7 Hz, 1H), 7.14 (s, 1H), 7.09 (d, *J* = 7.7 Hz, 1H) 2.53 (s, 3H), 2.40 (s, 3H). **^{13}C NMR (101 MHz, $CDCl_3$)**: δ 143.5, 141.8, 132.4, 131.0, 127.0, 118.5, 109.7, 21.7, 20.4. **HRMS** (ESI+) *m/z* calcd. for C_9H_9N $[M+H]^+$: 132.0808; found: 132.0813.

2,6-Dimethylbenzonitrile (21)

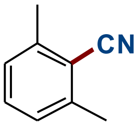

Prepared according to the general procedure in Scheme 3 using 2-bromo-1,3-dimethylbenzene (0.133 mL, 1.0 mmol) and P(*t*Bu)$_3$-Pd-G3 (28.6 mg, 0.05 mmol). Product was purified by flash column chromatography using pentane/Et_2O (10:1) as eluent which afforded **21** as a colorless solid (**Run 1**: 126.2 mg, 96%. **Run 2:** 125.7 mg, 96%). **^{1}H NMR (400 MHz, $CDCl_3$)**: δ 7.34 (t, *J* = 7.7 Hz, 1H), 7.12 (d, *J* = 7.7 Hz, 2H), 2.53 (s, 6H). **^{13}C NMR (101 MHz, $CDCl_3$)**: δ 142.3 (2), 132.2, 127.4 (2), 117.4, 113.5, 20.9 (2). **HRMS** (ESI+) *m/z* calcd. for C_9H_9N $[M+H]^+$: 132.0808; found: 132.0810.

2-(Hydroxymethyl)benzonitrile (22)

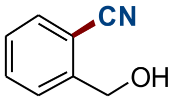

Prepared according to the general procedure in Scheme 3 using (2-bromophenyl) methanol (187.0 mg, 1.0 mmol). Product was purified by flash column chromatography using pentane/acetone (5:1) as eluent which afforded **22** as a yellow solid (**Run 1**: 126.2 mg, 95%. **Run 2:** 127.9 mg, 96%). **^{1}H NMR (400 MHz, $CDCl_3$)**: δ 7.94 (d, *J* = 7.6 Hz, 1H), 7.69 (td, *J* = 7.6, 0.9 Hz, 1H), 7.56-7.49 (m, 2H), 5.33 (s, 2H). **^{13}C NMR (101 MHz, $CDCl_3$)**: δ 171.1, 146.5, 134.0, 129.1, 125.8, 125.8, 122.1, 69.6. **HRMS** (ESI+) *m/z* calcd. for C_8H_7NO $[M+H]^+$: 134.0600; found: 134.0600.

4-Chlorobenzonitrile (23)

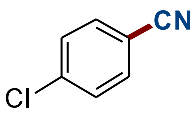

Prepared according to the general procedure in Scheme 3 using 1-bromo-4-chlorobenzene (182.0 mg, 1.0 mmol). Chamber A was only heated to 45 °C. Product was purified by flash column chromatography using pentane/EtOAc (20:1) as eluent which afforded **23** as a colorless solid (**Run 1**: 127.1 mg, 92%. **Run 2:** 115.1 mg, 84%). **^{1}H NMR (400 MHz, $CDCl_3$)**: δ 7.60 (d, *J* = 8.6 Hz, 2H), 7.47 (d, *J* = 8.6 Hz, 2H). **^{13}C NMR (101 MHz, $CDCl_3$)**: δ 139.7, 133.5 (2), 129.9 (2), 118.1, 110.9. **HRMS** (ESI+) *m/z* calcd. for C_7H_4ClN $[M+H]^+$: 138.0105; found: 138.0108.

5-Cyano-isobenzofuran-1-one (24)

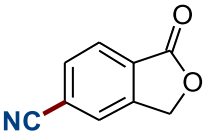

Prepared according to the general procedure in Scheme 3 using 5-bromoisobenzofuran-1(*3H*)-one (213.0 mg, 1.0 mmol) and P(*t*Bu)$_3$-Pd-G3 (28.6 mg, 0.05 mmol). Product was purified by flash column chromatography using pentane/acetone (4.5:1) as eluent which afforded **24** as colorless solid (**Run 1**: 132.4 mg, 83%. **Run 2:** 134.8 mg, 85%). **^{1}H NMR (400 MHz, CDCl$_3$)**: δ 8.06 (d, *J* = 8.3 Hz, 1H), 7.84 (d, *J* = 8.3 Hz, 1H), 7.84 (s, 1H), 5.40 (s, 2H). **^{13}C NMR (101 MHz, CDCl$_3$)**: δ 169.1, 146.8, 133.1, 129.7, 126.9, 126.5, 117.8, 117.6, 69.4. **HRMS** (ESI+) *m/z* calcd. for $C_9H_5NO_2$ [M+H]$^+$: 160.0393; found: 160.0393.

Benzofuran-5-carbonitrile (25)

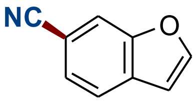

Prepared according to the general procedure in Scheme 3 using 5-bromobenzofuran (197.0 mg, 1.0 mmol). Product was purified by flash column chromatography using pentane/Et_2O (5:1) as eluent which afforded **25** as a colorless solid (**Run 1**: 130.2 mg, 91%. **Run 2:** 134.3 mg, 94%). **^{1}H NMR (400 MHz, CDCl$_3$)**: δ 7.96 (s, 1H), 7.75 (d, *J* = 2.1 Hz, 1H), 7.59 (s, 2H), 6.85 (d, *J* = 2.1 Hz, 1H). **^{13}C NMR (101 MHz, CDCl$_3$)**: δ 156.7, 147.3, 128.2, 128.1, 126.5, 119.6, 112.8, 106.9, 106.8. **HRMS** (ESI+) *m/z* calcd. for C_9H_5NO [M+H]$^+$: 144.0444; found: 144.0445.

Quinoline-3-carbonitrile (26)

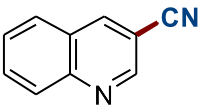

Prepared according to the general procedure in Scheme 3 using 3-bromoquinoline (208.1 mg, 1.0 mmol). Product was purified by flash column chromatography using pentane/acetone (3:1) as eluent which afforded **26** as a colorless solid (**Run 1**: 148.1 mg, 96%. **Run 2:** 152.1 mg, 99%). **^{1}H NMR (400 MHz, CDCl$_3$)**: δ 9.05 (d, *J* = 1.9 Hz, 1H), 8.55 (d, *J* = 1.9 Hz, 1H), 8.18 (d, *J* = 8.8 Hz, 1H), 7.92-7.90 (m, 2H), 7.72 (dt, *J* = 7.9, 0.6 Hz, 1H). **^{13}C NMR (101 MHz, CDCl$_3$)**: δ 149.9, 149.0, 141.6, 132.9, 130.0, 128.7, 128.4, 126.4, 117.3, 106.8. **HRMS** (ESI+) *m/z* calcd. for $C_{10}H_6N_2$ [M+H]$^+$: 155.0604; found: 155.0605.

1*H*-Indole-5-carbonitrile (27)

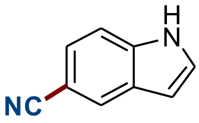

Prepared according to the general procedure in Scheme 3 using 5-bromoindole (196.0 mg, 1.0 mmol). Product was purified by flash column chromatography using pentane/Et_2O (2.5:1) as eluent which afforded **27** as a colorless solid (**Run 1**: 136.5 mg, 95%. **Run 2:** 133.5 mg, 94%). **^{1}H NMR (400 MHz, CDCl$_3$)**: δ 8.57 (bs, 1H), 8.00 (s, 1H), 7.48-7.42 (m, 2H), 7.35 (t, *J* =2.6 Hz, 1H), 6.66-6.62 (m, 1H). **^{13}C NMR (101 MHz, CDCl$_3$)**: δ 137.6, 127.8, 126.6, 126.5, 125.1, 120.9, 112.1, 103.7, 103.1. **HRMS** (ESI+) *m/z* calcd. for $C_9H_6N_2$ [M+H]$^+$: 143.0604; found: 143.0605.

Methyl 5-cyanopyridine-2-carboxylate (28)

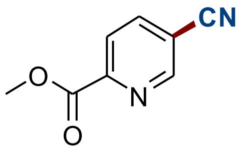

Prepared according to the general procedure in Scheme 3 using methyl 5-bromopicolinate (216.0 mg, 1.0 mmol) and P(*t*Bu)$_3$-Pd-G3 (28.6 mg, 0.05 mmol). Product was purified by flash column chromatography using pentane/acetone (7:1) as eluent which afforded **28** as a colorless solid (**Run 1**: 118.9 mg, 73%. **Run 2:** 131.7 mg, 81%).^{1}**H NMR (400 MHz, CDCl$_3$)**: δ 9.03 (d, *J* = 1.1 Hz, 1H), 8.28 (dd, *J* = 8.0, 1.1 Hz, 1H), 8.17 (dd, *J* = 8.0, 2.0 Hz, 1H), 4.08 (s, 3H). ^{13}**C NMR (101 MHz, CDCl$_3$)**: δ 164.4, 152.5, 150.7, 140.9, 125.0, 116.0, 113.2, 53.8. **HRMS** (ESI+) *m/z* calcd. for $C_8H_6N_2O_2$ $[M+H]^+$: 163.0502; found: 163.0502.

Methyl 5-cyanopyridine-2-carboxylate-(^{13}CN) (28-^{13}C)

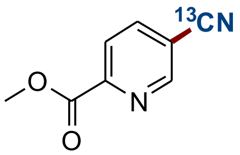

Prepared according to the general procedure in Scheme 3 using methyl 5-bromopicolinate (216.0 mg, 1.0 mmol), K^{13}CN (99.2 mg, 1.5 mmol) instead of KCN and P(*t*Bu)$_3$-Pd-G3 (28.6 mg, 0.05 mmol). Product was purified by flash column chromatography using pentane/EtOAc (4:1) as eluent which afforded **28-^{13}C** as a colorless solid (128.2 mg, 79%) ^{1}**H NMR (400 MHz, CDCl$_3$)**: δ 9.01-9.00 (m, 1H), 8.26 (ad, *J* = 8.1 Hz, 1H), 8.15 (ddd, *J* = 8.1, 5.2, 2.0 Hz, 1H), 4.05 (s, 3H). ^{13}**C NMR (101 MHz, CDCl$_3$)**: δ 164.3, 152.4 (d, *J* = 3.9 Hz, 1C), 150.6 (d, *J* = 1.4 Hz, 1C), 140.8 (d, *J* = 2.1 Hz, 1C), 124.9 (d, *J* = 4.8 Hz, 1C), 115.9, 113.1 (d, *J* = 84.4 Hz, 1C), 53.7. **HRMS** (ESI+) *m/z* calcd. for $C_7{}^{13}CH_6N_2O_2$ $[M+H]^+$: 164.0536; found: 164.0536.

Starting materials and procedures for Scheme 4

Synthesis of Dapivirine

2-Chloro-*N*-mesitylpyridine-4-amine

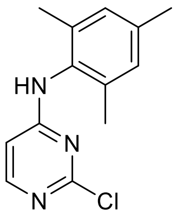

In a 250 mL round bottom flask 2,4-dichloropyrimidine (2.98 g, 20.0 mmol) was dissolved in *n*BuOH (100 mL). 2,4,6-Trimethylaniline (2.70 g, 20.0 mmol) and DIPEA (4.5 mL, 26.0 mmol) was added and the mixture was heated to 160 °C. After 24 hours the solvent was removed under reduced pressure and the crude was dissolved in EtOAc (50 mL) and H_2O (50 mL). The organic layer was separated and the aqueous layer was extracted with EtOAc (3 x 20 mL). The combined organic layers was washed brine (50 mL), dried over $MgSO_4$, filtered and concentrated. Purification by flash column chromatography (10:1 pentane:EtOAc to 4:1 pentane:EtOAc) afforded the title compound as an orange solid (3.30 g, 67%). ^{1}**H NMR (400 MHz, CDCl$_3$)**: δ 8.00 (d, *J* = 5.8 Hz, 1H), 6.97 (s, 2H), 6.98 (bs, 1H), 5.83 (d, *J* = 5.8 Hz, 1H), 2.31 (s, 3H), 2.16 (s, 6H). ^{13}**C NMR (101 MHz, CDCl$_3$)**: δ 163.8, 160.8, 158.2, 138.3, 136.4 (2C), 130.7, 129.6 (2C), 100.9, 21.0, 18.1 (2C) **HRMS** (ESI+) *m/z* calcd. for $C_{13}H_{14}ClN_3$ $[M+H]^+$: 248.0949; found: 248.0949.

2-(4-Bromophenyl)-*N*-mesitylpyrimidin-4-amine (29a)

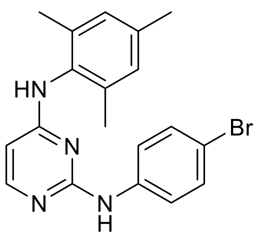

A 500 mL round bottom flask was charged with 2-chloro-*N*-mesitylpyridine-4-amine (3.30 g, 13.32 mmol), 4-bromoaniline (2.29 g, 13.32 mmol) and a stir bar. Isopropanol (200 mL) was added and the mixture was stirred for 30 min before TFA (0.102 mL, 1.33 mmol) was added and the mixture was heated to reflux. After 24 hours NaOH (20 mL) was added, and the mixture was evaporated dry. Purification by flash column chromatography (6:1 pentane: acetone to 3:1 pentane:acetone) afforded **29a** as an off white solid (4.23 g, 84%). **^{1}H NMR (400 MHz, $CDCl_3$)**: δ 7.93 (d, *J* = 5.7 Hz, 1H), 7.55-7.29 (m, 4H), 7.24-7.20 (m, 1H), 6.97 (s, 2H), 6.64 (bs, 1H), 5.47 (bs, 1H), 2.33 (s, 3H), 2.19 (s, 6H). **^{13}C NMR (101 MHz, $CDCl_3$)**: δ 163.0, 159.8, 157.3, 139.3, 137.6, 136.8, 131.9, 131.7, 129.4, 120.8, 114.2, 95.2, 21.1, 18.4. **HRMS** (ESI+) *m/z* calcd. for $C_{19}H_{19}BrN_4$ $[M+H]^+$: 383.0866; found: 383.0871.

4-((4-(Mesitylamino)pyrimidin-2-yl)amino)benzonitrile (29, Dapivirine)

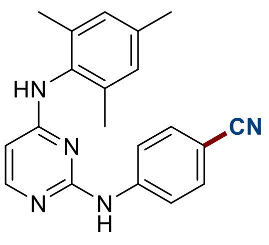

Prepared according to the general procedure in Scheme 3 using 2-(4-bromophenyl)-*N*-mesitylpyrimidin-4-amine (383.3 mg, 1.0 mmol), P(*t*Bu)$_3$-Pd-G3 (28.6 mg, 0.05 mmol), KOAc (294.5 mg, 3.0 mmol) and 2 mL of dioxane. Product was purified by flash column chromatography using pentane/acetone (5:1) as eluent which afforded **29** as a colorless solid (**Run 1**: 322.8 mg, 98%. **Run 2:** 310.2 mg). **^{1}H NMR (400 MHz, $CDCl_3$)**: δ 7.97 (d, *J* = 5.3 Hz, 1H), 7.83-7.47 (m, 4H), 7.41 (s, 1H), 6.98 (s, 2H), 6.45 (bs, 1H), 5.54 (bs, 1H), 2.34 (s, 3H), 2.20 (s, 6H). **^{13}C NMR (101 MHz, $CDCl_3$)**: δ 163.0, 159.3 (2C), 157.3, 144.4 (2C), 137.9, 136.7, 133.2 (2C), 131.6, 129.5, 119.8, 118.4, 104.0, 96.1, 21.1, 18.4. **HRMS** (ESI+) *m/z* calcd. for $C_{20}H_{19}N_5$ $[M+H]^+$: 330.1713; found: 330.1725.

Synthesis of Citalopram

3-(5-Bromo-1-(4-fluorophenyl)-1,3-dihydroisobenzofuran-1-yl)-*N*,*N*-dimethylpropan-1-amine (30a)

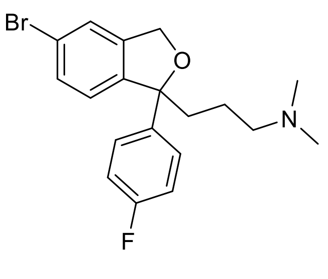

The corresponding aryl bromide of Citalopram was synthesised by the procedure reported in the literature. (*J. Med. Chem.* **2010**, *53*, 6112). **^{1}H NMR (400 MHz, $CDCl_3$)**: δ 7.45-7.40 (m, 3H), 7.34 (s, 1H), 7.21 (d, *J* = 8.1 Hz, 1H), 6.99 (dd, *J* = 8.7 Hz, 2H), 5.13 (d, *J* = 12.7 Hz, 1H), 5.08 (d, *J* = 12.7 Hz, 1H), 2.65-2.57 (m, 2H), 2.42 (s, 6H), 2.30-2.15 (m, 2H), 1.69-1.62 (m, 1H), 1.61-1.46 (m, 1H) **^{13}C NMR (101 MHz, $CDCl_3$)**: δ 162.1 (d, *J* = 246 Hz), 143.1, 141.4, 140.1 (d, *J* = 2.5 Hz), 131.0, 126.8 (d, *J* = 8.0 Hz, 2C), 124.7, 123.5, 121.9, 115.4 (d, *J* = 21.3 Hz, 2C), 90.7, 71.4, 58.8, 44.2 (2C), 38.7, 20.9. ^{19}F NMR (376 MHz, $CDCl_3$): δ -115.6 **HRMS** (ESI+) *m/z* calcd. for $C_{19}H_{21}BrFNO$ $[M+H]^+$: 378.0863; found: 378.0864.

1-(3-(dimethylamino)propyl)-1-(4-fluorophenyl)-1,3-dihydroisobenzofuran-5-carbonitrile (30, Citalopram)

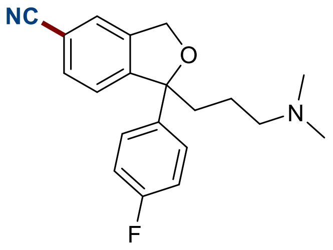

Prepared according to the general procedure in Scheme 3 using 3-(5-Bromo-1-(4-fluorophenyl)-1,3-dihydroisobenzofuran-1-yl)-*N*,*N*-dimethylpropan-1-amine (378.3 mg, 1.0 mmol) and P(*t*Bu)$_3$-Pd-G3 (28.6 mg, 0.05 mmol). Product was purified by flash column chromatography using chloroform/MeOH (20:1) as eluent which afforded **30** as a yellow liquid (**Run 1**: 281.9 mg, 89%. **Run 2:** 274.9 mg, 85%). **^{1}H NMR (400 MHz, CDCl$_3$)**: δ 7.59 (d, *J* = 7.9 Hz, 1H), 7.49 (s, 1H), 7.45-7.24 (m, 3H), 7.00 (dd, *J* = 8.7, 8.6 Hz, 2H), 5.20 (d, *J* = 13.0 Hz, 1H), 5.14 (d, *J* = 13.0 Hz, 1H), 2.49 (t, *J* = 7.2 Hz, 2H), 2.35-2.15 (m, 2H), 2.32 (s, 6H), 1.62-1.53 (m, 1H), 1.49-1.39 (m, 1H). **^{13}C NMR (101 MHz, CDCl$_3$)**: δ 162.2 (d, *J* = 246.5 Hz, 1C), 149.3, 140.3, 139.3 (d, *J* = 3.1 Hz, 1C), 132.1, 126.8 (d, *J* = 8.1 Hz, 2C), 125.3, 122.9, 118.7, 115.6 (d, *J* = 21.4 Hz, 2C), 112.0, 91.1, 71.4, 59.0, 44.6 (2C), 38.7, 21.4. ^{19}F NMR (376 MHz, CDCl$_3$): δ -115.2 **HRMS** (ESI+) *m/z* calcd. for $C_{20}H_{21}FN_2O$ $[M+H]^+$: 325.1711; found: 325.1718.

Synthesis of Letrozole

Bis(4-bromophenyl)methanol

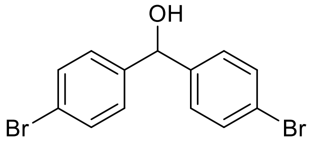

A 500 mL round-bottom flask was under argon charged with 1,4-dibromobenzene (18.87 g, 80 mmol), a stirbar and anhydrous THF (200 mL). The mixture was cooled to -78 °C before *n*-BuLi (50 mL, 80 mmol, 1.6M in hexane) was added dropwise. The reaction was stirred for 20 minutes before a solution of 4-bromobenzaldehyde (14.80 g, 80 mmol) dissolved in anhydrous THF (50 mL) was added dropwise. The reaction was stirred for 30 minutes before cooling was removed. Once the reaction had reached room temperature H_2O (100 mL) was added and the organic phase was separated, and the aqueous phase was extracted with EtOAc (3x 40 mL). The combined organic phases were dried over $MgSO_4$, filtered and concentrated under reduced pressure. The crude was triturated with heptane to obtain a white solid, which was washed with heptane to afford the desired product (23.22 g, 85%). **^{1}H NMR (400 MHz, CDCl$_3$)**: δ 7.47 (d, *J* = 8.4Hz, 4H), 7.23 (d, *J* = 8.4 Hz, 4H), 5.75 (d, *J* = 3.0 Hz, 1H), 2.19 (d, *J* = 3.0 Hz, 1H). **^{13}C NMR (101 MHz, CDCl$_3$)**: δ 142.4 (2C), 131.9 (4C), 128.3 (4C), 121.9 (2C), 75.2.

1-(Bis(4-bromophenyl)methyl)-1*H*-1,2,4-triazole (31a)

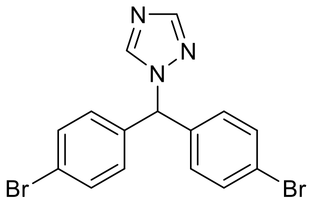

In a 500 mL round-bottom flask bis(4-bromophenyl)methanol (23.94 g, 70 mmol) was under an inert atmosphere dissolved in CH_2Cl_2 (250 mL) and the mixture was cooled to 0 °C before thionyl chloride (50.1 mL, 0.7 mol) was added dropwise. Cooling was removed and the reaction was stirred for two hours after which the reaction was concentrated under reduced pressure. Full conversion to product was verified by ^{1}H NMR. The crude was under an inert atmosphere dissolved in DMF (200 mL), added 1,2,4-triazole (7.25 g, 105 mmol) and K_2CO_3 (14.51 g, 105 mmol) after which the reaction was heated to 80 °C for 24 hours. The reaction was poured into cold water, and the aqueous phase was extracted with Et_2O (6x 50 mL). The combined organic phases were washed with brine (2x 100 mL), dried over $MgSO_4$, filtered and concentrated under reduced pressure which afforded a yellow solid. Recrystallization from 96% EtOH afforded **31a** as a white crystalline solid (20.86 g, 76%). **^{1}H NMR (400 MHz,**

$CDCl_3$): δ 8.03 (s, 1H), 7.96 (s, 1H), 7.52 (d, *J* = 8.4Hz, 4H), 7.01 (d, *J* = 8.4 Hz, 4H), 6.65 (s, 1H). **^{13}C NMR (101 MHz, $CDCl_3$)**: δ 152.8, 143.6, 136.7 (2C), 132.4 (4C), 129.9 (4C), 123.3 (2C), 66.7. **HRMS** (ESI+) *m/z* calcd. for $C_{15}H_{11}Br_2N_3$ $[M+H]^+$: 391.9392; found: 391.9388.

1-(Bis(4-cyanophenyl)methyl)-1*H*-1,2,4-triazole (31, Letrozole).

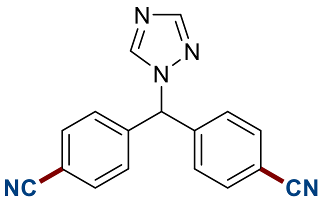

Prepared according to the general procedure in Scheme 3 using 1-(Bis(4-bromophenyl)methyl)-1*H*-1,2,4-triazole (393.1 mg, 1.0 mmol), P(*t*Bu)$_3$-Pd-G3 (28.6 mg, 0.05 mmol), KOAc (392.6 mg, 4.0 mmol) and KCN (195.4 mg, 3.0 mmol). Product was purified by flash column chromatography using EtOAc/pentane (3:1) as eluent which afforded **31** as a colorless solid (**Run 1**: 283.2 mg, 99%. **Run 2:** 271.2 mg, 95%). **^1H NMR (400 MHz, $CDCl_3$)**: δ 8.08 (s, 1H), 8.07 (s, 1H), 7.71 (d, *J* = 8.3Hz, 4H), 7.28 (d, *J* = 8.3 Hz, 4H), 6.80 (s, 1H). **^{13}C NMR (101 MHz, $CDCl_3$)**: δ 153.1, 143.7 (2C), 141.8, 133.0 (4C), 128.9 (4C), 117.8 (2C), 113.4 (2C), 66.4. **HRMS** (ESI+) *m/z* calcd. for $C_{17}H_{11}N_5$ $[M+H]^+$: 286.1087; found: 286.1089.

For the reaction on a 5 mmol scale, a 100 mL two-chamber reactor was used. To chamber A of the two-chamber system P(*t*Bu)$_3$-Pd-G3 (143.0 mg, 0.25 mmol), KOAc (1.963 g, 20.0 mmol), **31a** (1.195 g, 5.0 mmol), dioxane (5 mL) and H_2O (10 mL) were added. To chamber B of the two-chamber system KCN (0.997 g, 15 mmol) and ethylene glycol (5 mL) were added. Lastly, AcOH (4.29 mL, 75 mmol) was added carefully on top of the ethylene glycol layer. The reaction was performed as according to the general procedure for Scheme 3. Product was purified by flash column chromatography using EtOAc/pentane (3:1) as eluent which afforded **31** as a colorless solid (1.35 g, 95%).

For the reaction on a 10 mmol scale, a 200 mL two-chamber reactor was used. To chamber A of the two-chamber system P(*t*Bu)$_3$-Pd-G3 (286.0 mg, 0.5 mmol), KOAc (3.926 g, 40 mmol), **31a** (3.93 g, 10.0 mmol), dioxane (10 mL) and H_2O (20 mL) were added. To chamber B of the two-chamber system KCN (1.954 g, 30 mmol) and ethylene glycol (10 mL) were added. Lastly, AcOH (8.59 mL, 150 mmol) was added carefully on top of the ethylene glycol layer. The reaction was performed as according to the general procedure for Scheme 3. Product was purified by flash column chromatography using EtOAc/pentane (3:1) as eluent which afforded **31** as a colorless solid (2.42g, 85%).

Mechanistic investigations

Isolation of compounds 32 and 33

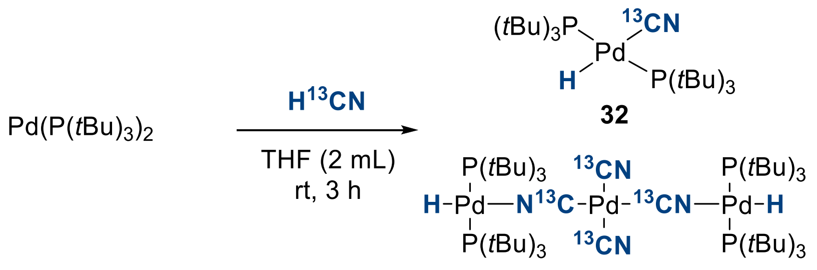

In an argon filled glovebox, a two-chamber system made from two glass vials (8 mL each) connected by a glass tube was utilized for the reaction. To the HCN consuming chamber (A) $Pd(P(tBu)_3)_2$ (102.2 mg, 0.2 mmol), THF (2 mL) and a stir bar were charged. To the HCN producing chamber (B) $K^{13}CN$ (39.7 mg, 0.6 mmol), ethylene glycol (1 mL), octanoic acid (0.38 mL, 2.4 mmol) and a stir bar were charged. Chamber B was heated to 40 °C and stirring was initiated. After 3 hours the content of chamber A was transferred to a 8 mL vial and the solvent was removed under reduced pressure. This gave a colorless solid (99.2 mg) which consisted of a mixture of **32** and **33**. To the solid was added THF (6 mL) and the mixture was heated to reflux. As the solution cooled down, **33** recrystallized from the solution as colorless needles. The crystals were filtered and washed with pentane which gave crystals of **33** that were suitable for X-ray analysis. The mother liquid was dissolved in CH_2Cl_2 and layered with heptane. After two days colorless crystals had emerged. The solution was decanted and the crystals were gently washed with heptane. This afforded colorless crystals of **32** which were suitable for X-ray analysis.

(P(*t*Bu)$_3$)$_2$Pd(H)(^{13}CN) (32)

^{1}H NMR (400 MHz, THF-d$_8$): δ 1.56 (apt, *J* = 6.2 Hz, 54H), -11.42 (dt, *J* = 53.0, 4.7 Hz, 1H). **^{13}C NMR (101 MHz, THF-d$_8$)**: δ 149.4 (dt, *J* = 11.7, 3.5 Hz, 1C), 39.8 (t, *J* = 4.3 Hz, 6C), 33.0 (t, *J* = 2.5 Hz, 18C). **^{31}P NMR (161.9 MHz, THF-d$_8$)**: δ 93.1 (d, *J* = 11.7 Hz, 2P).

Compound 33

^{1}H NMR (400 MHz, CDCl$_3$): δ 1.52 (apt, *J* = 6.3 Hz, 108H), -16.79 (apsept, 2H). **^{13}C NMR (101 MHz, CDCl$_3$)**: δ 141.7 (t, *J* = 7.3 Hz, 2C), 126.3 (t, *J* = 7.3 Hz, 2C), 39.4 (t, *J* = 4.3 Hz, 12C), 32.9 (t, *J* = 4.0 Hz, 36C). **^{31}P NMR (161.9 MHz, CDCl$_3$)**: δ 84.7.

Procedure for Table 2

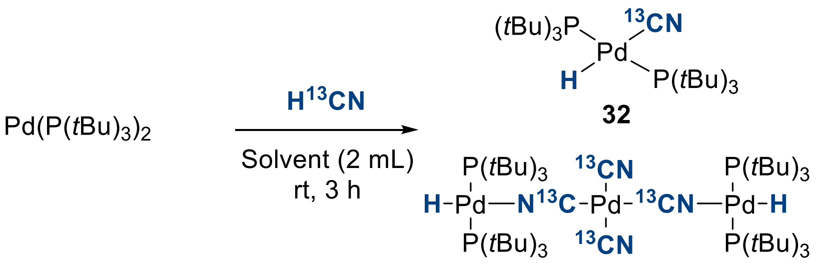

32

33

In an argon filled glovebox, a two-chamber system made from two glass vials (8 mL each) connected with a glass tube, the HCN consuming chamber (A) was charged with $Pd(P(tBu)_3)_2$ (102.2 mg, 0.2 mmol), solvent (2 mL), a stir bar and for entry 2–6 KOAc. The HCN producing chamber (B) was charged with $K^{13}CN$, ethylene glycol (1 mL), octanoic acid (4 equiv compared to $K^{13}CN$) and a stir bar. After 3 hours the content of chamber A was transferred to a 50 mL round bottom flask and evaporated dry. Crude NMR was used to deduce the distribution between the different Pd-species.

Procedure for Scheme 6:

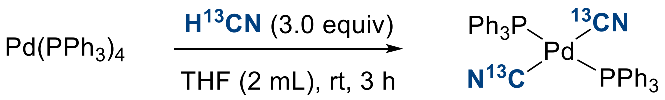

34

In an argon filled glovebox, a two-chamber system made from two glass vials (8 mL each) connected with a glass tube, the HCN consuming chamber (A) was charged with $Pd(PPh_3)_4$ (115.6 mg, 0.1 mmol), THF (2 mL) and a stir bar. The HCN producing chamber (B) was charged with $K^{13}CN$ (19.8 mg, 0.3 mmol), ethylene glycol (0.5 mL), octanoic acid (0.19 mL, 2.4 mmol) and a stir bar. Stirring was initiated at room temperature. After 3 hours the content of chamber A was transferred to a 8 mL vial, diluted with THF (3 mL) and heated to reflux. The solution was allowed to cool to rt. After 14 hours the solution was decanted and the crystals were washed with pentane. This gave both colorless and yellow crystals. The yellow crystals were determined to be $Pd(PPh_3)_4$, while the colorless crystals were determined to be compound **34**. The colorless crystals could easily be separated from the yellow $Pd(PPh_3)_4$ crystals by manual separation, thereby obtaining crystals of **34** which were suitable for X-ray analysis.

$(PPh_3)_2Pd(^{13}CN)_2$ (34)

^{1}**H NMR (400 MHz, CDCl$_3$)**: δ 7.71-7.66 (m, 4H), 7.51-7.41 (m, 6H). ^{13}**C NMR (101 MHz, CDCl$_3$)**: δ 134.6 (t, *J* = 6.5 Hz, 6C), 131.1 (t, *J* = 15.6 Hz, 2C), 128.6 (t, *J* = 5.5 Hz, 6C). ^{31}**P NMR (161.9 MHz, CDCl$_3$)**: δ 23.4 (t, *J* = 15.6 Hz).

Due to low relaxation we weren't able to get a good ^{13}C NMR of compound **34**. Even after 8192 scans it was only possible to get 3 signals in ^{13}C NMR although there should be 5.

Procedure for Scheme 7:

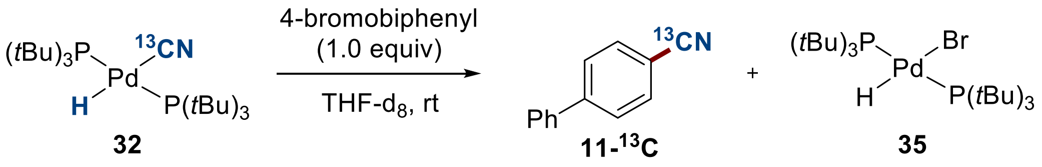

In an argon filled glovebox, **32** (10.8 mg, 0.02 mmol) and 4-bromo-1,1'-biphenyl (4.7 mg, 0.02 mmol) were added to a 4 mL vial. The content was transferred with THF-d$_8$ (total volume 0.8 mL) to a NMR-tube. Mesitylene (2.0 µL) was added as an internal standard. The NMR-tube was sealed and quickly placed in a NMR apparatus, where the reaction was followed by ^{1}H- and ^{31}P NMR.

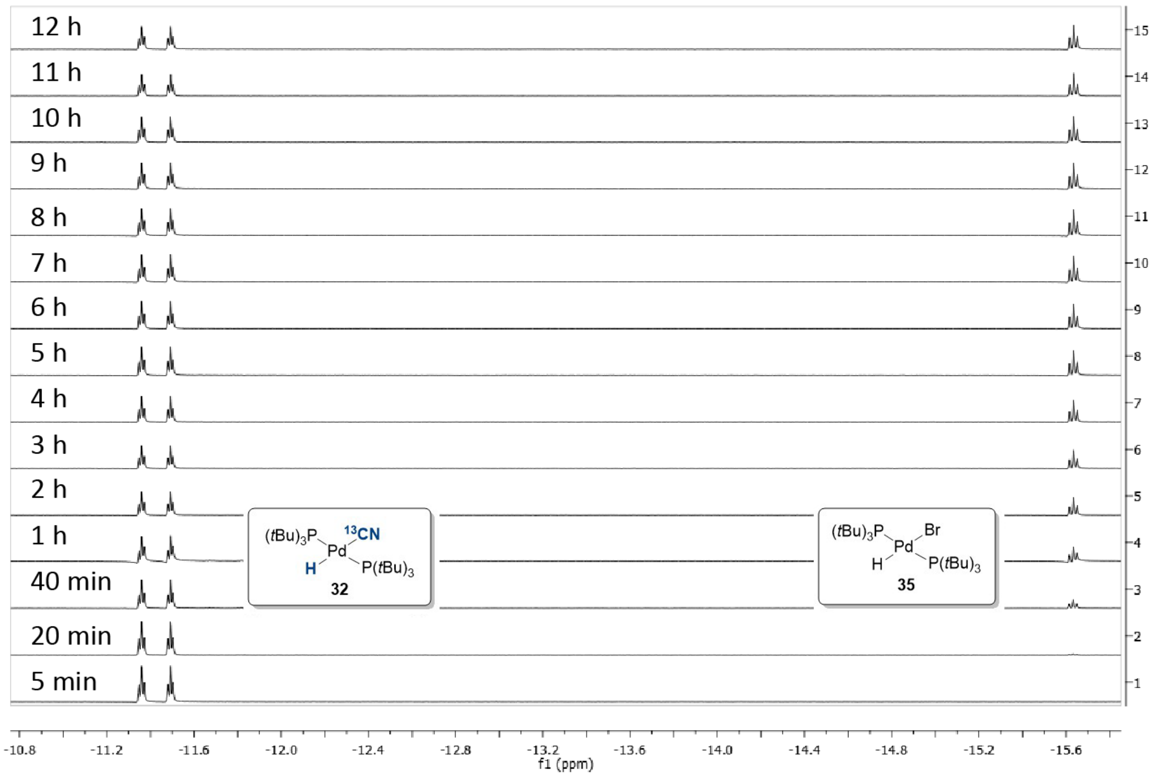

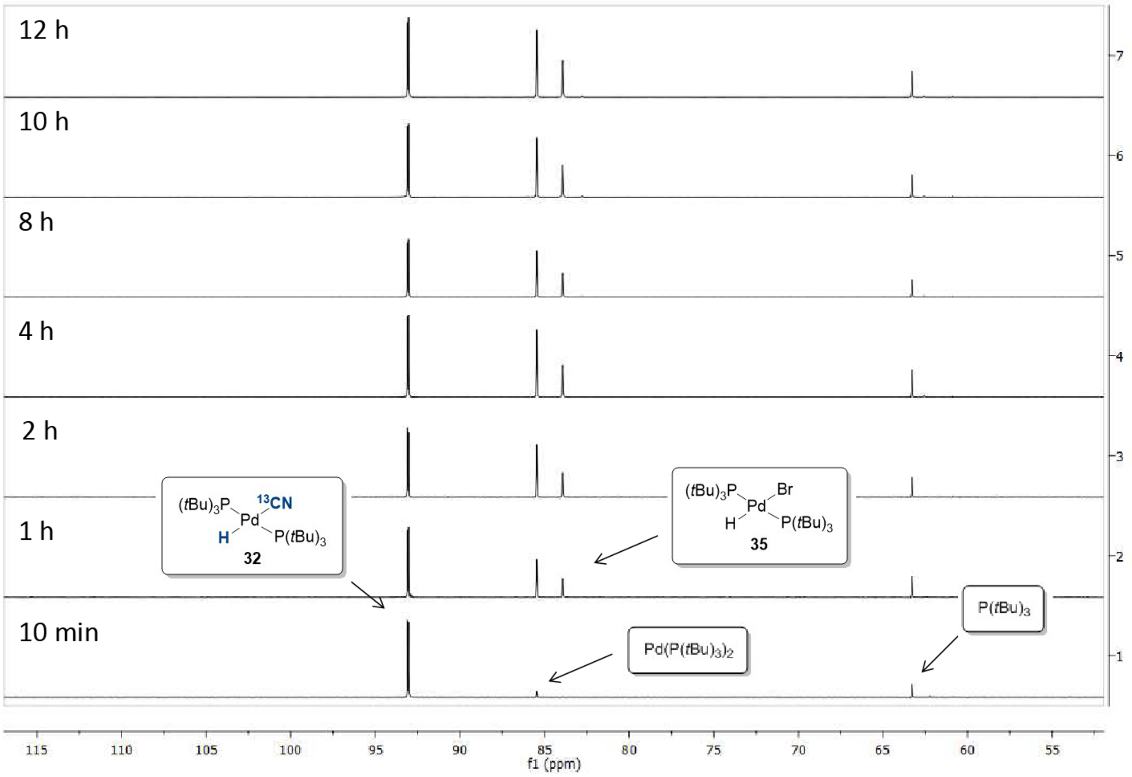

Formation of 35:

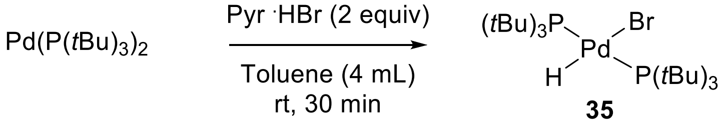

In an argon filled glovebox, Pd(P(*t*Bu)$_3$)$_2$ (51.1 mg, 0.1 mmol) was dissolved in toluene (4 mL) in a 50 mL round bottom flask. Pyridine-HBr (32.0 mg, 0.2 mmol) was dissolved in CH_3CN (1.6 mL) and added dropwise after which the solution was stirred for 30 min. The mixture was evaporated dry, and heptane (7.0 mL) was triturated into the flask and the mixture was cooled to -37 ^{o}C for 4 hours. The product was filtered and washed with heptane (10x2 mL) to obtain compound **35** as a green solid (31.1 mg, 52%). A sample of 10.0 mg was dissolved in CH_2Cl_2 (0.2 mL) and layered with heptane (2.5 mL). After one day, fine crystals had emerged, which were suitable for X-ray analysis.

(P(*t*Bu)$_3$)Pd(H)(Br) (35)

^{1}H NMR (400 MHz, THF-d$_8$): δ 1.59 (t, *J* = 6.2 Hz, 54H), -15.63 (t, *J* = 7.2 Hz, 1H). **^{13}C NMR (101 MHz, THF-d$_8$)**: δ 40.5 (t, *J* = 3.6 Hz, 6C), 33.5 (t, *J* = 2.8 Hz, 18C). **^{31}P NMR (161.9 MHz, THF-d$_8$)**: δ 83.9 (d, *J* = 1.9 Hz, 2P).

Formation of 36:

(P(*t*Bu)$_3$)Pd(Ph)(Br) (36)

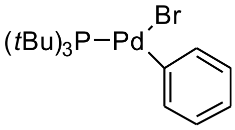

In an argon filled glovebox, a 8 mL vial was charged with Pd(P(*t*Bu)$_3$)$_2$ (102.2 mg, 0.2 mmol), bromobenzene (0.94 mL, 9.0 mmol) and a stir bar. The solution was heated to 70 °C under stirring. After 2.5 hour ^{31}P NMR deemed the reaction done. The solution was transferred with THF to a 50 mL round bottom flask whereafter it was evaporated dry. Pentane (12 mL) was added which resulted in product precipitation. The solution was stirred for 10 min, before it was filtered under suction. The product was washed with pentane (5x2mL) which afforded **36** (74.0 mg, 79%) as a yellow solid.

^{1}H NMR (400 MHz, THF-d$_8$): δ 7.11 (d, *J* = 5.1 Hz, 2H), 6.70-6.60 (m, 3H), 1.36 (d, *J* = 12.4 Hz, 27H). **^{31}P NMR (161.9 MHz, THF-d$_8$)**: δ 62.7

Procedure for Scheme 9a:

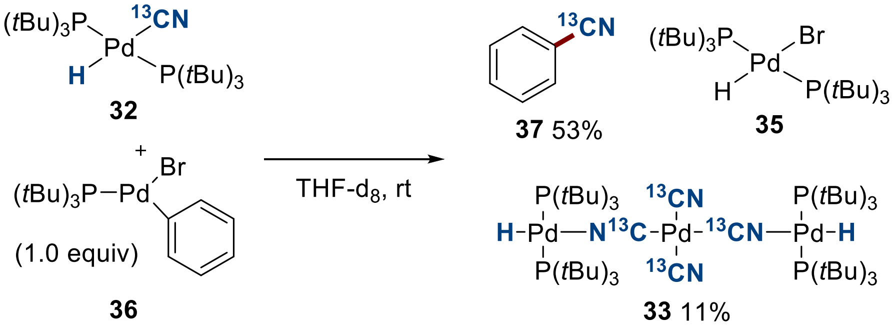

In an argon filled glovebox, **32** (10.8 mg, 0.02 mmol) and **36** (9.3 mg, 0.02 mmol) were added to a 4 mL vial. The mixture was transferred to a NMR-tube with THF-d$_8$ (total volume 0.8 mL). Mesitylene (2.0 µL) was added as an internal standard. The NMR tube was sealed and removed from the glovebox, and NMR was used to follow the reaction progress.

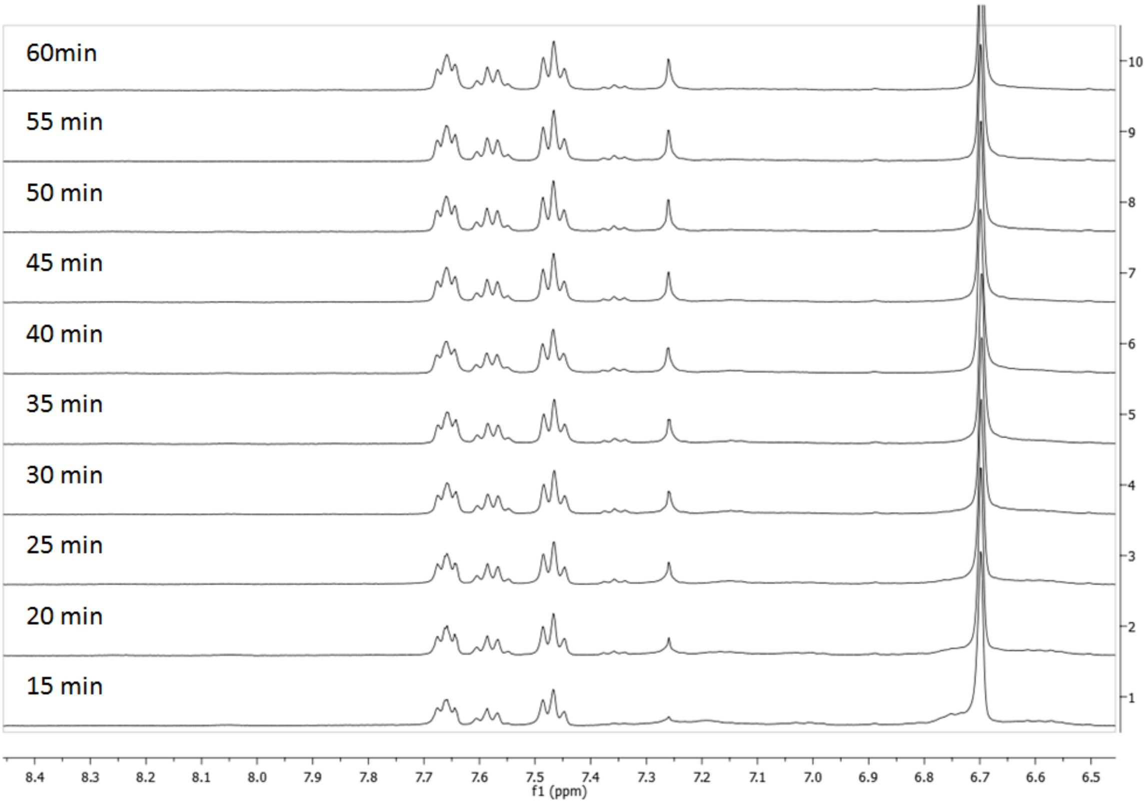

60min
55 min
50 min
45 min
40 min
35 min
30 min
25 min
20 min
15 min
f1 (ppm)

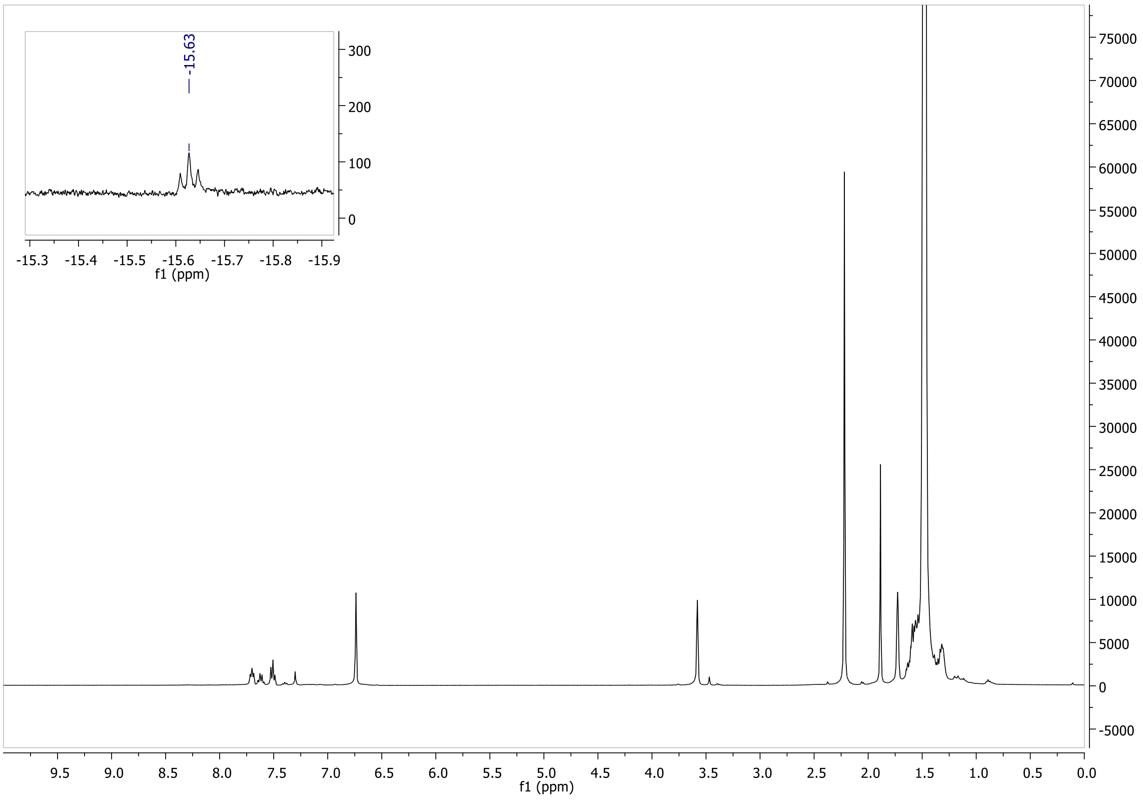

-15.63
f1 (ppm)
f1 (ppm)

Procedure for Scheme 9b:

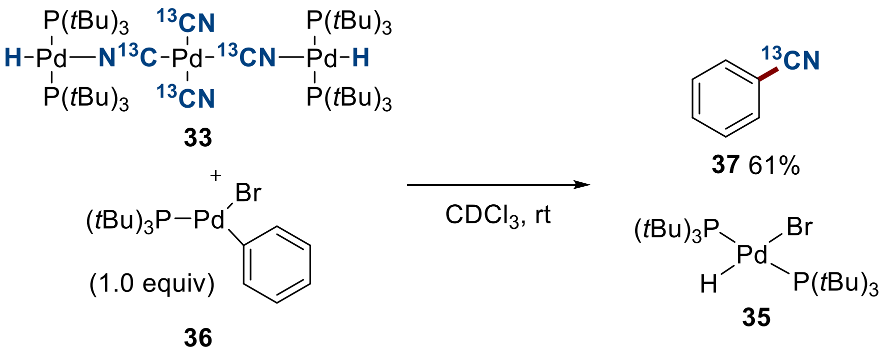

In an argon filled glovebox, **33** (8.0 mg, 0.0065 mmol) and **36** (3.0 mg, 0.02 mmol) were added to a 4 mL vial. The mixture was transferred to a NMR-tube with $CDCl_3$ (total volume 0.8 mL). Mesitylene (20.0 µL) was added as an internal standard. The NMR tube was sealed and removed from the glovebox, and NMR was used to follow the reaction progress.

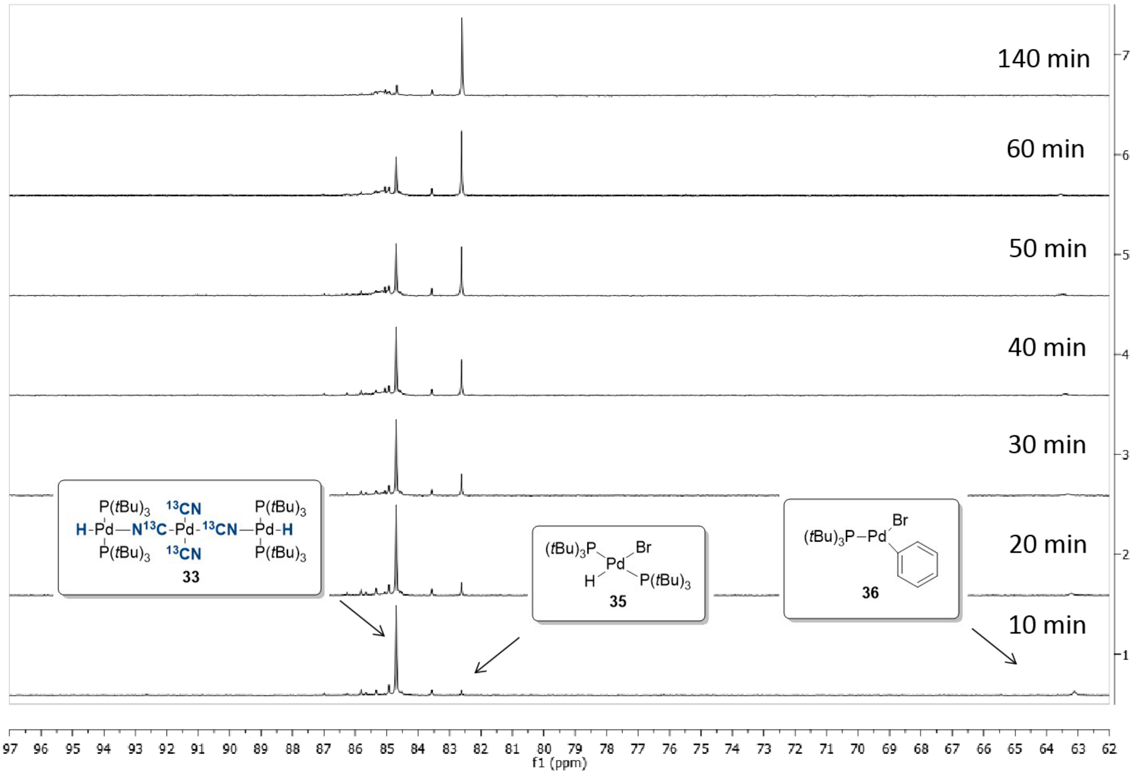

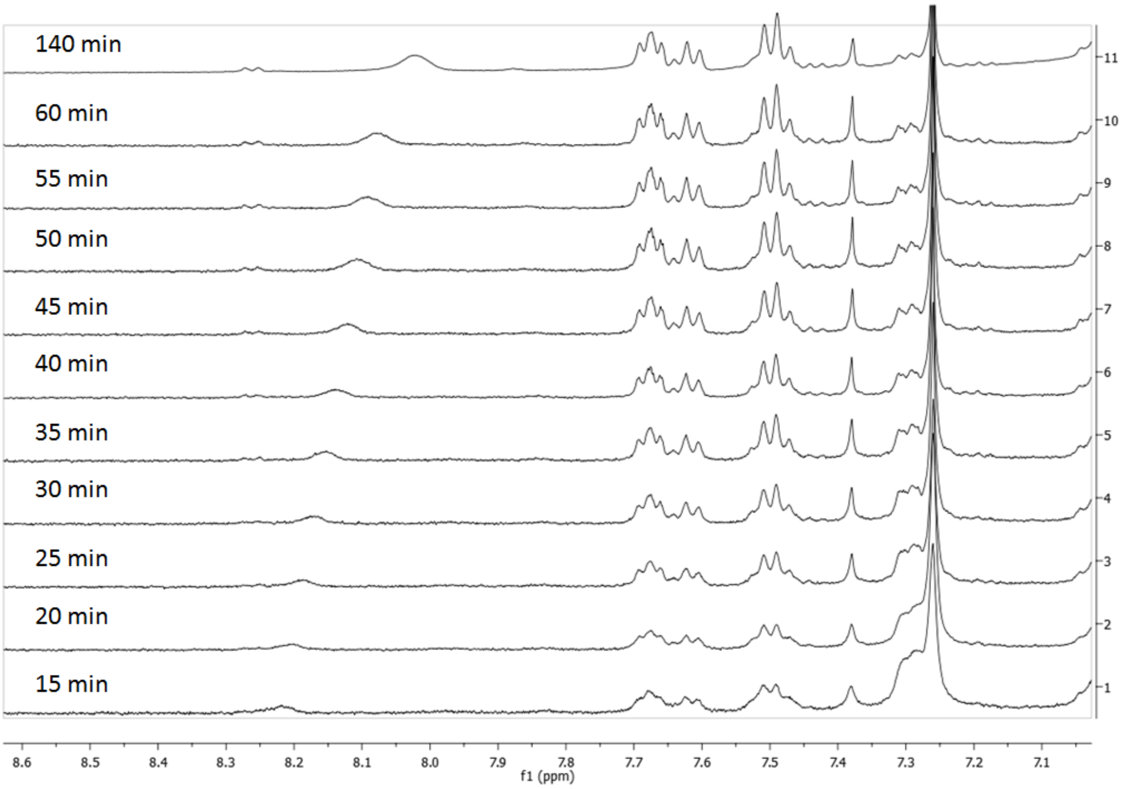

Procedure for Scheme 9c:

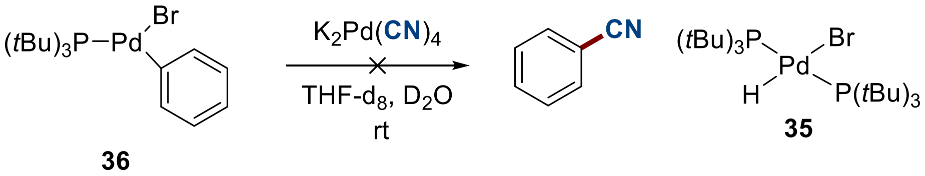

In an argon filled glovebox, **36** (9.3 mg, 0.02 mmol) and $K_2Pd(CN)_4x(H_2O)$ (6.1 mg, 0.02 mmol) were added to a NMR-tube along with THF-d_8 (0.7 mL) and D_2O (0.1 mL). Mesitylene (2.0 μL) was added as an internal standard. The NMR tube was sealed and removed from the glovebox, and NMR was used to follow the reaction progress.

Procedure for Scheme 10:

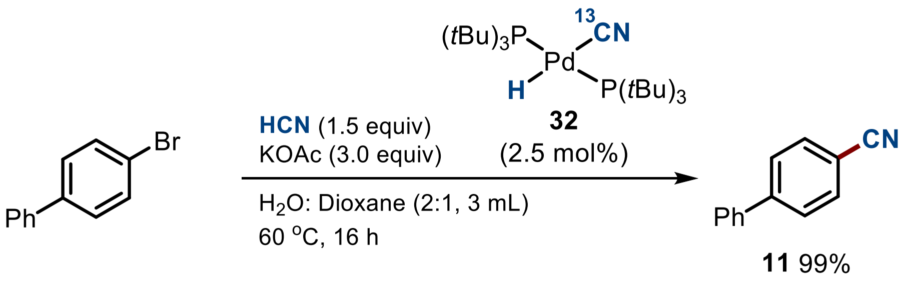

Prepared according to the general procedure in Scheme 3 using 4-bromo-1,1'-biphenyl (233.1 mg, 1.0 mmol) and **32** (13.5 mg, 0.025 mmol). Product was purified by flash column chromatography using pentane/Et_2O (15:1) as eluent which afforded **11** as a colorless solid (178.0 mg, 99%).

Procedure for Scheme 11:

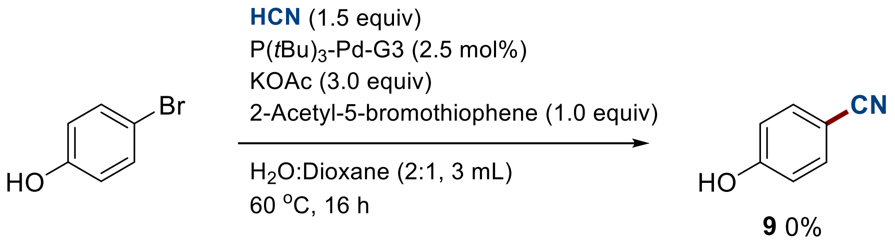

Prepared according to the general procedure in Scheme 3 using 4-bromophenol (173.0 mg, 1.0 mmol) and 2-acetyl-5-bromothiophene (205.1 mg, 1.0 mmol). Crude NMR with mesitylene as an internal standard showed no conversion to the desired benzonitrile.

The reaction was repeated with 0.5 equiv of 2-acetyl-5-bromothiophene, which also resulted in no conversion to the desired benzonitrile.

Procedure for Scheme 12:

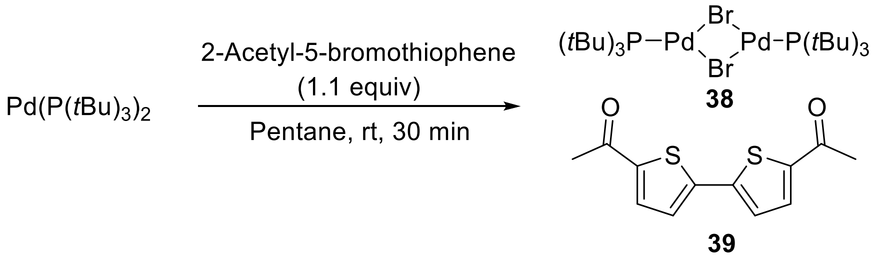

In an argon filled glovebox, a 8 mL vial was charged with $Pd(P(tBu)_3)_2$ (51.1 mg, 0.1 mmol), 2-acetyl-5-bromothiophene (22.6 mg, 0.11 mmol), pentane (3 mL) and a stir bar. After 24 hours the reaction was filtered and washed with pentane (3x2 mL). The compound was dried under high vacuum which afforded a mixture of **38** and **39**. 5 mg of the mixture was dissolved in CH_2Cl_2 (0.2 mL) and layered with heptane (2.0 mL). After two days fine green crystals of **38** had emerged which were suitable for X-ray analysis.

Crystallographic details

Table S13. Crystallographic information for the four structures solved using single crystal X-ray diffraction. Four diffraction experiments (**32** - **35**) were performed on a SuperNova diffractometer from Agilent Technologies, using $Mo_{K\alpha}$ radiation (λ= 0.71073 Å). The diffracted intensities were collected on a CCD detector and data were integrated and corrected for absorption using CrysAlisPro.[1] The last diffraction experiment (**38**) was conducted on a Apex2 diffractometer from Bruker, using A$g_{K\alpha}$ radiation (λ= 0.56086 Å). Data were collected on a CCD detector, integrated using SAINT+[2] and absorption-corrected in SADABS.[3] The structure solution and refinement were carried out with SHELXT, using Olex2, for all compounds.[4]

Sample	**Complex 32**	**Complex 33**	**Complex 34**	**Complex 35**	**Complex 38**
**Chemical fromula**	$C_{25}H_{54}NP_2Pd$	$C_{52}H_{110}N_4P_4Pd_3$	$C_{38}H_{30}N_2P_2Pd$	$C_{24}H_{55}BrP_2Pd$	$C_{24}H_{54}Br_2P_2Pd_2$
**M_r / g × mol^{-1}**	537.03	1234.51	682.98	591.93	777.21
**T / K**	100.00(11)	100.00(10)	100.00(14)	100.00(10)	296.15
**Crystal system**	triclinic	monoclinic	triclinic	monoclinic	monoclinic
**Space group**	$P\bar{1}$	*P2₁/c*	$P\bar{1}$	*C2/c*	P21/n
**a/Å**	12.6035(3)	12.1372(2)	9.0667(3)	12.7069(4)	13.094(4)
**b/Å**	17.2591(4)	16.2799(2)	9.6985(4)	34.9448(9)	14.447(5)
**c/Å**	25.6455(5)	16.5783(3)	10.1017(3)	14.1937(6)	16.046(5)
**α/°**	90.2601(18)	90	111.130(3)	90	90
**β/°**	90.0672(18)	110.126(2)	93.150(3)	116.366(5)	92.715(7)
**γ/°**	90.8113(19)	90	108.191(3)	90	90
**Volume/Å3**	5577.9(2)	3075.74(10)	772.96(5)	5647.0(4)	3032.2(17)
**Z**	8	2	1	8	4
**ρ_{calc} / g × cm^{-3}**	1.279	1.333	1.467	1.392	1.703
**μ / mm^{-1}**	0.792	1.006	0.734	2.195	2.101
**F(000)**	2296	1296	348	2480	1560
**Crystal size / mm^3**	0.15×0.1×0.07	0.13×0.1×0.08	0.17×0.11×0.09	0.16×0.13×0.09	0.2 × 0.2 × 0.07
**$(\sin\theta/\lambda)_{max}$ / Å^{-1}**	0.624	0.624	0.624	0.714	0.833
**$N_{Tot,obs}$**	58488	25060	17898	42484	124681
**$N_{Uniq,obs}$**	22646	6204	3157	8624	14694
**$N_{Parameters}$**	1093	308	196	277	289
**GOF**	1.117	1	1.078	1.03	1.037
**R_{int}**	0.0421	0.0406	0.032	0.0389	0.0585
**R_1 , $R_1[F^2>2s(F^2)]$**	0.0842, 0.0601	0.0329, 0.0266	0.0220, 0.0201	0.0333, 0.0249	0.0792, 0.0500
**wR_2, $wR_2[F^2>2s(F^2)]$**	0.1373, 0.1272	0.0608, 0.0579	0.0513, 0.0502	0.0556, 0.0523	0.1468, 0.1266
**$\Delta\rho_{max}$, $\Delta\rho_{min}$ / e Å^{-3}**	1.87, -0.98	0.65, -0.47	0.45, -0.37	0.76, -0.98	7.24/-3.00

NMR Spectra

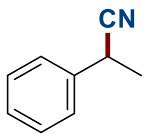

1

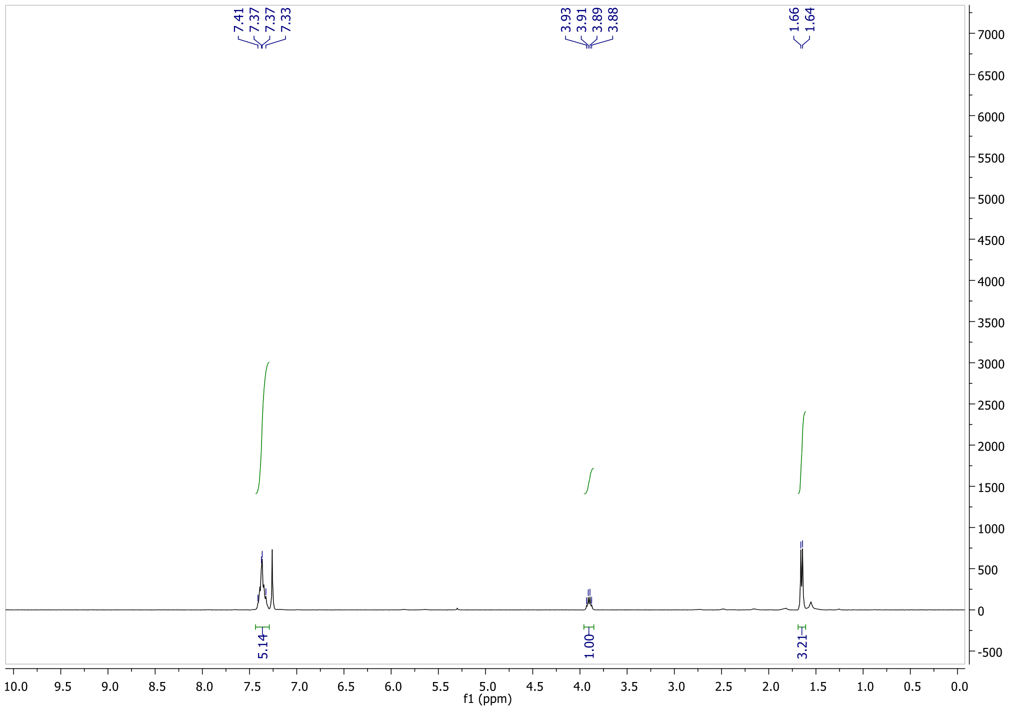

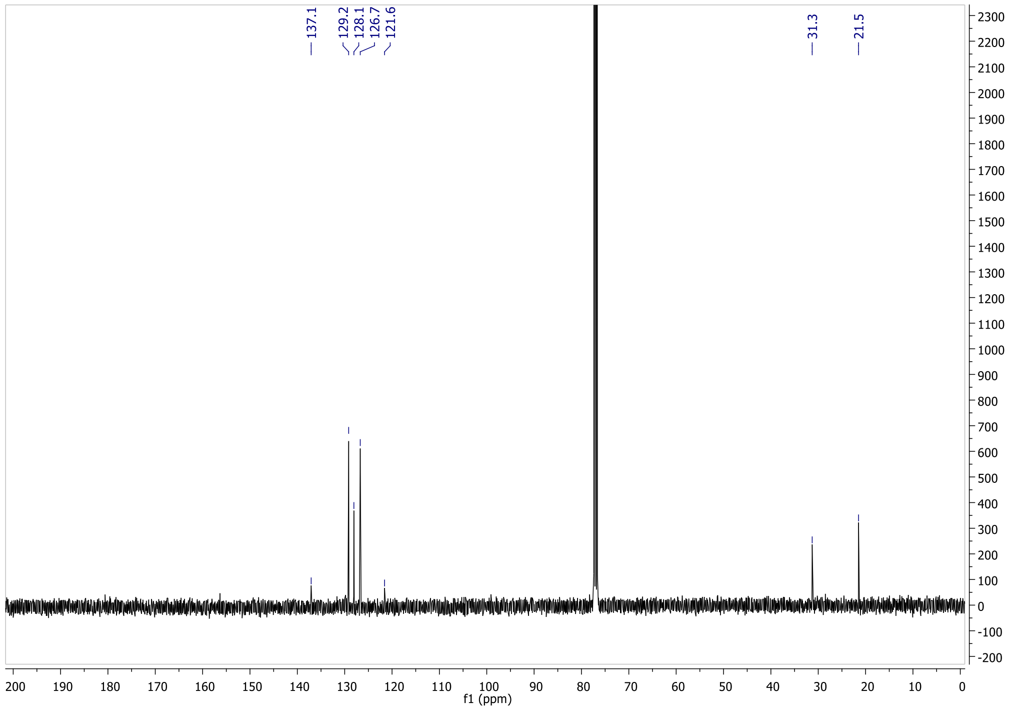

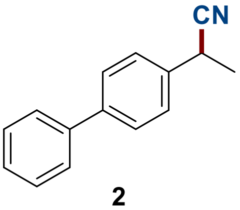
CN

2

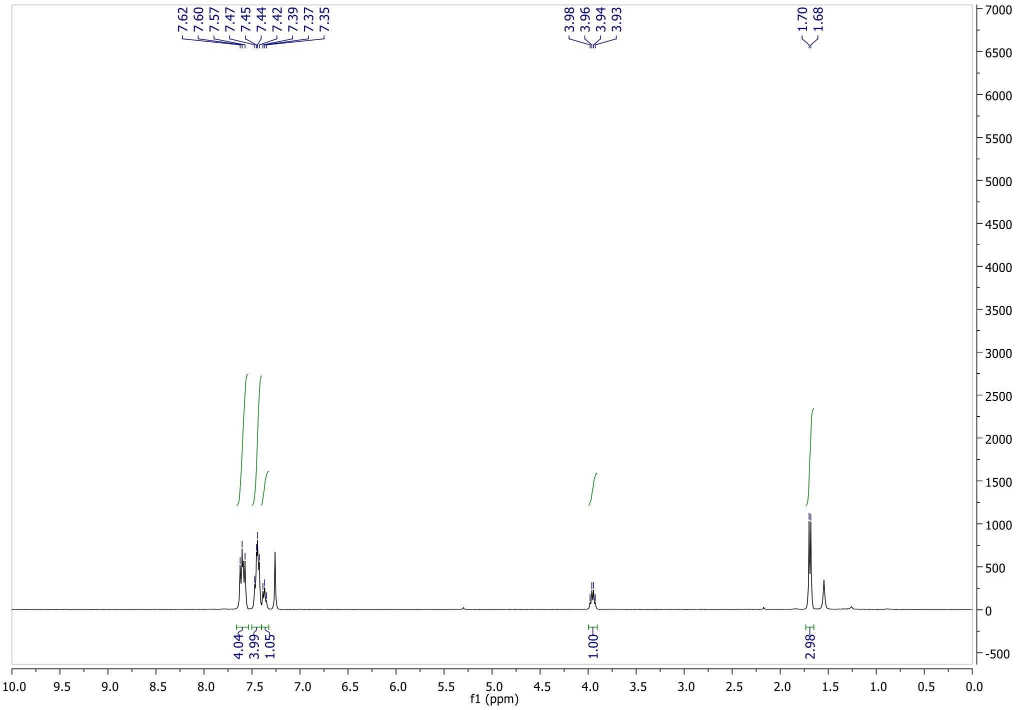
7.62
7.60
7.57
7.47
7.45
7.44
7.42
7.39
7.37
7.35
3.98
3.96
3.94
3.93
1.70
1.68
4.04
3.99
1.05
1.00
2.98
f1 (ppm)

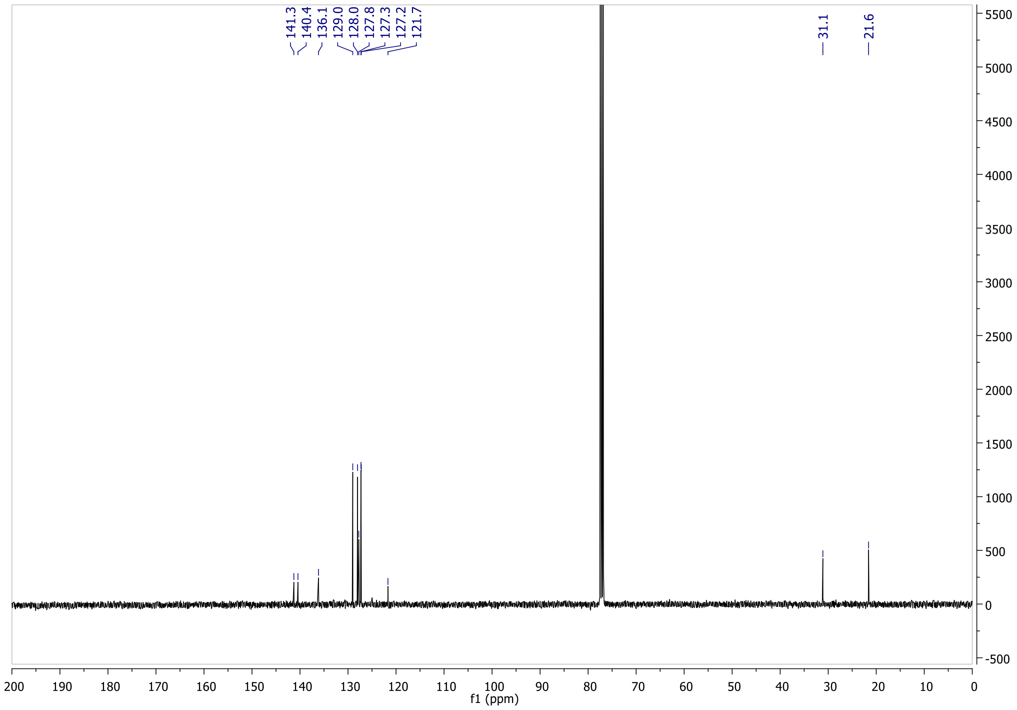
141.3
140.4
136.1
129.0
128.0
127.8
127.3
127.2
121.7
31.1
21.6
f1 (ppm)

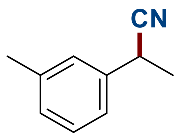

3

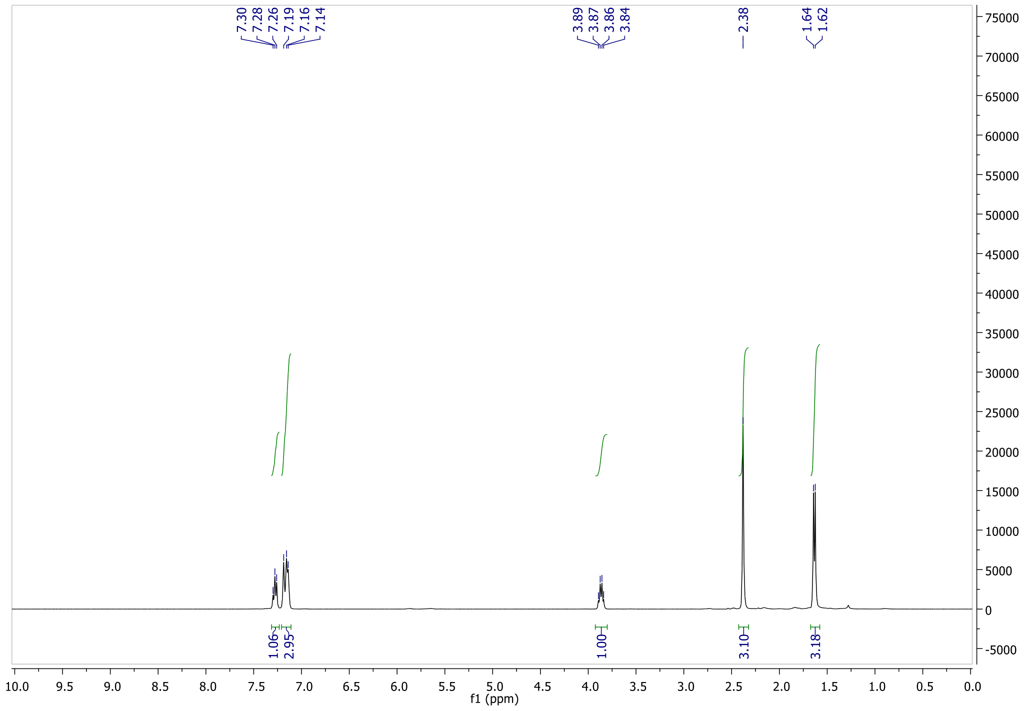

7.30
7.28
7.26
7.19
7.16
7.14
3.89
3.87
3.86
3.84
2.38
1.64
1.62
1.06
2.95
1.00
3.10
3.18
f1 (ppm)

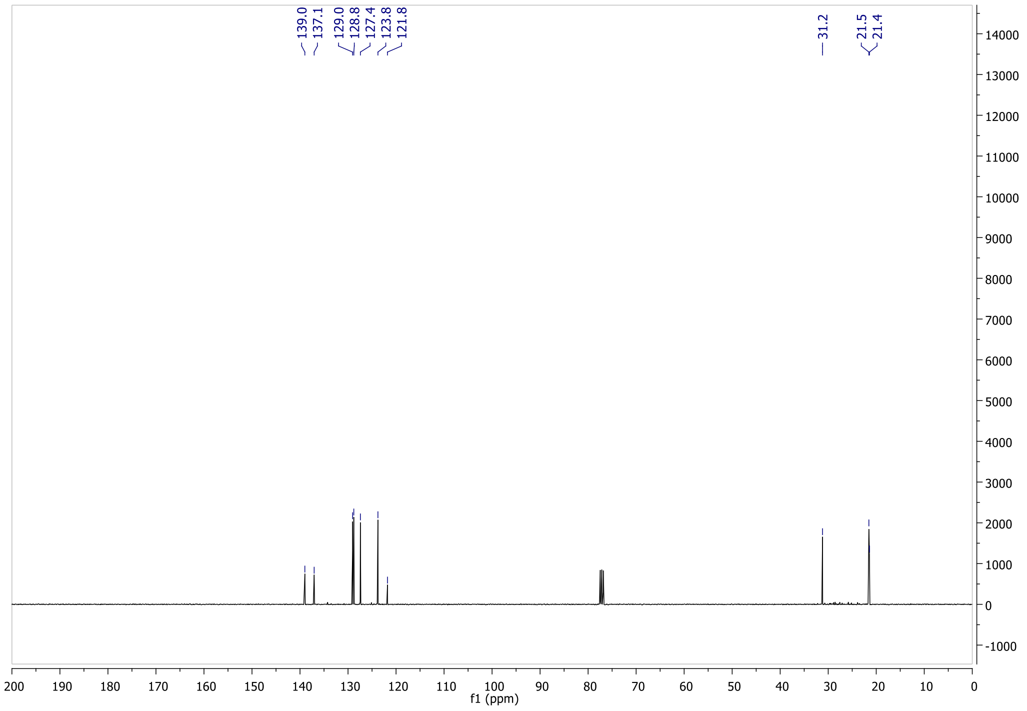

139.0
137.1
129.0
128.8
127.4
123.8
121.8
31.2
21.5
21.4
f1 (ppm)

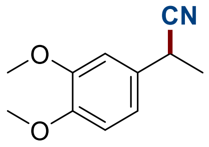
CN
O
O

4

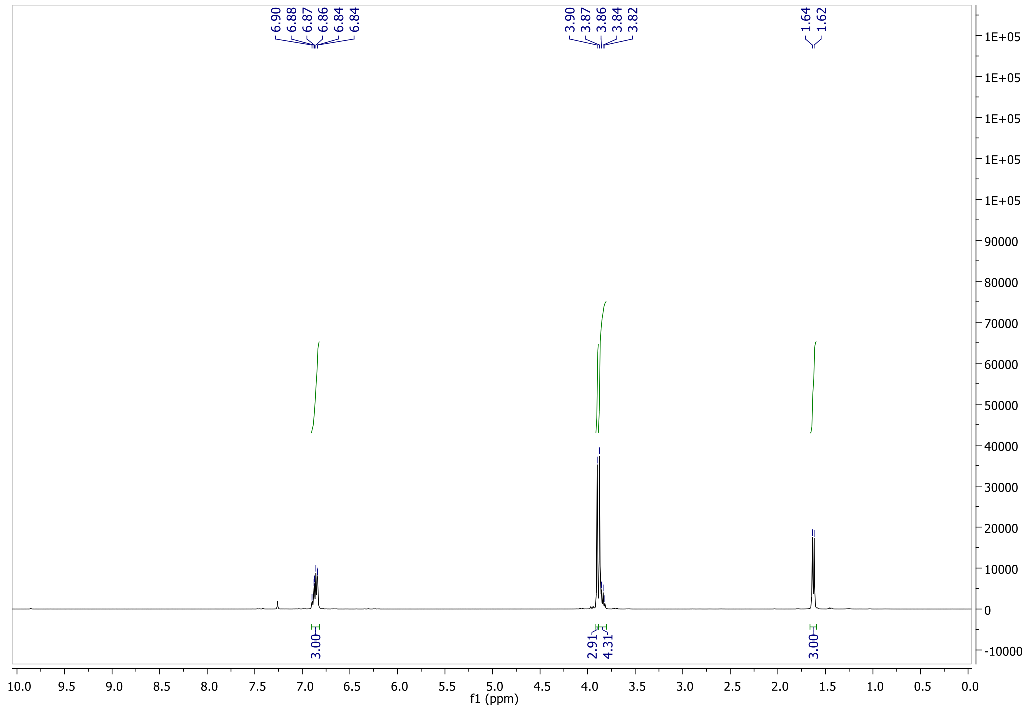
6.90
6.88
6.87
6.86
6.84
6.84
3.90
3.87
3.86
3.84
3.82
1.64
1.62
3.00
2.91
4.31
3.00
f1 (ppm)

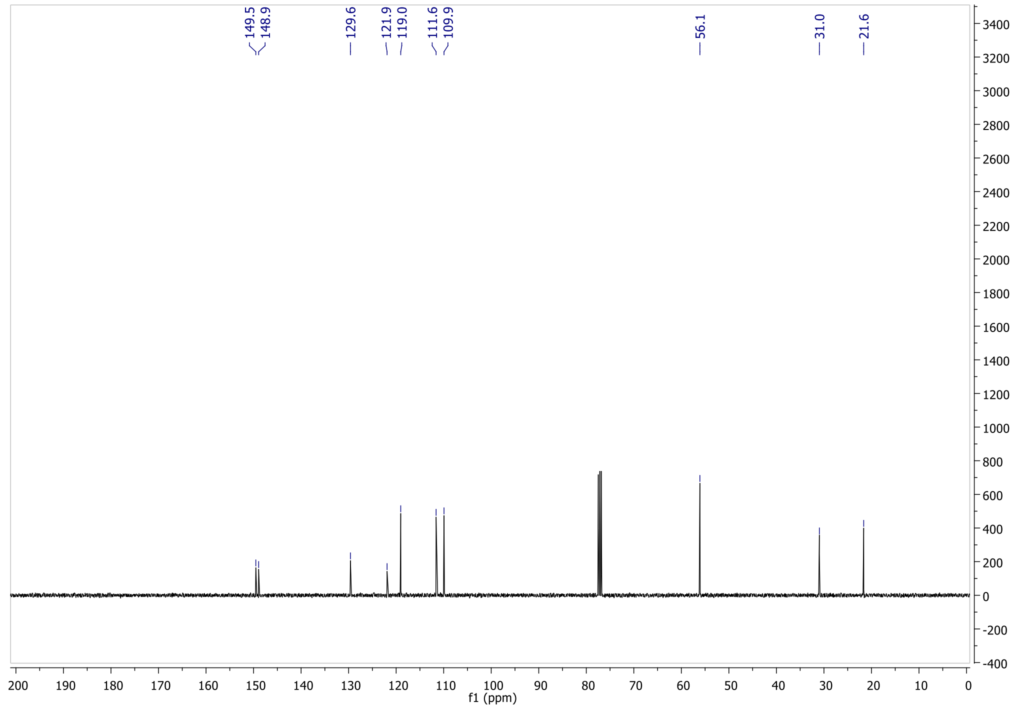
149.5
148.9
129.6
121.9
119.0
111.6
109.9
56.1
31.0
21.6
f1 (ppm)

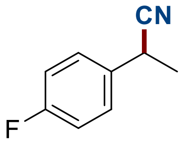

5

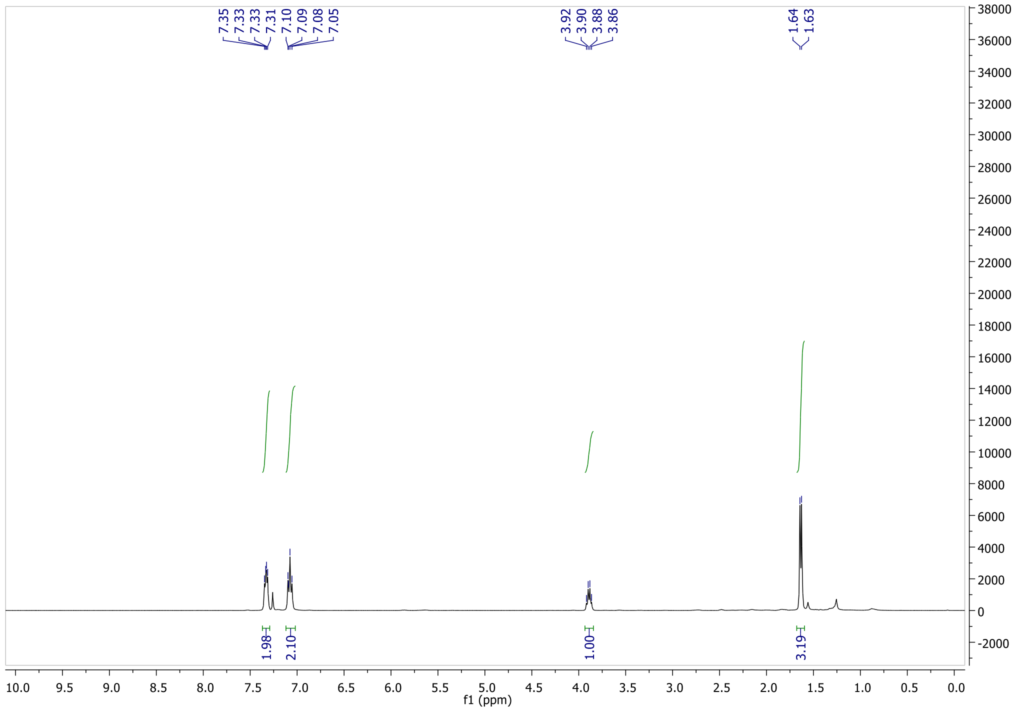

7.35
7.33
7.33
7.31
7.10
7.09
7.08
7.05
3.92
3.90
3.88
3.86
1.64
1.63
1.98
2.10
1.00
3.19
f1 (ppm)

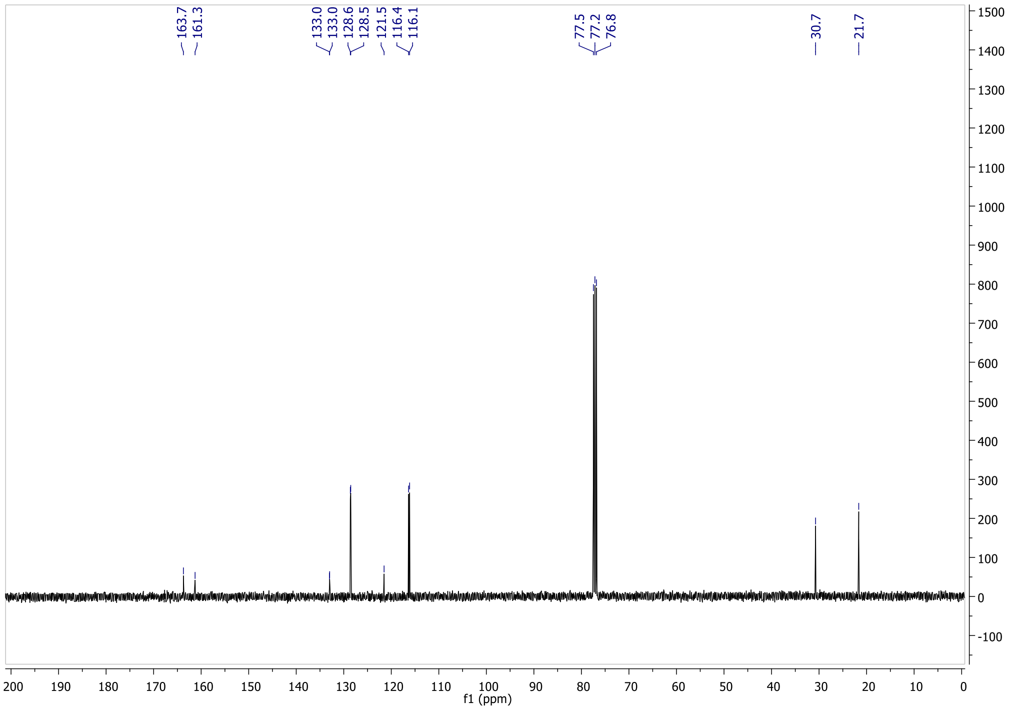

163.7
161.3
133.0
133.0
128.6
128.5
121.5
116.4
116.1
77.5
77.2
76.8
30.7
21.7
f1 (ppm)

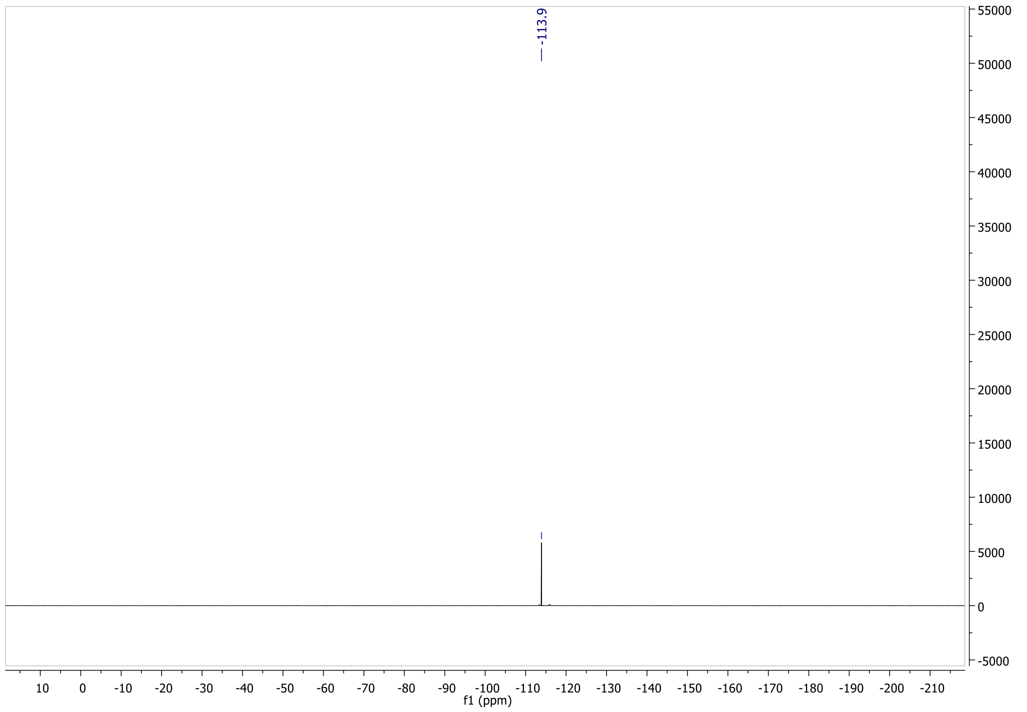
-113.9
55000
50000
45000
40000
35000
30000
25000
20000
15000
10000
5000
0
-5000
10 0 -10 -20 -30 -40 -50 -60 -70 -80 -90 -100 -110 -120 -130 -140 -150 -160 -170 -180 -190 -200 -210
f1 (ppm)

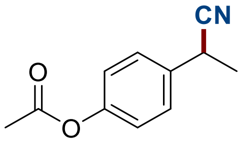
CN
O
O

6

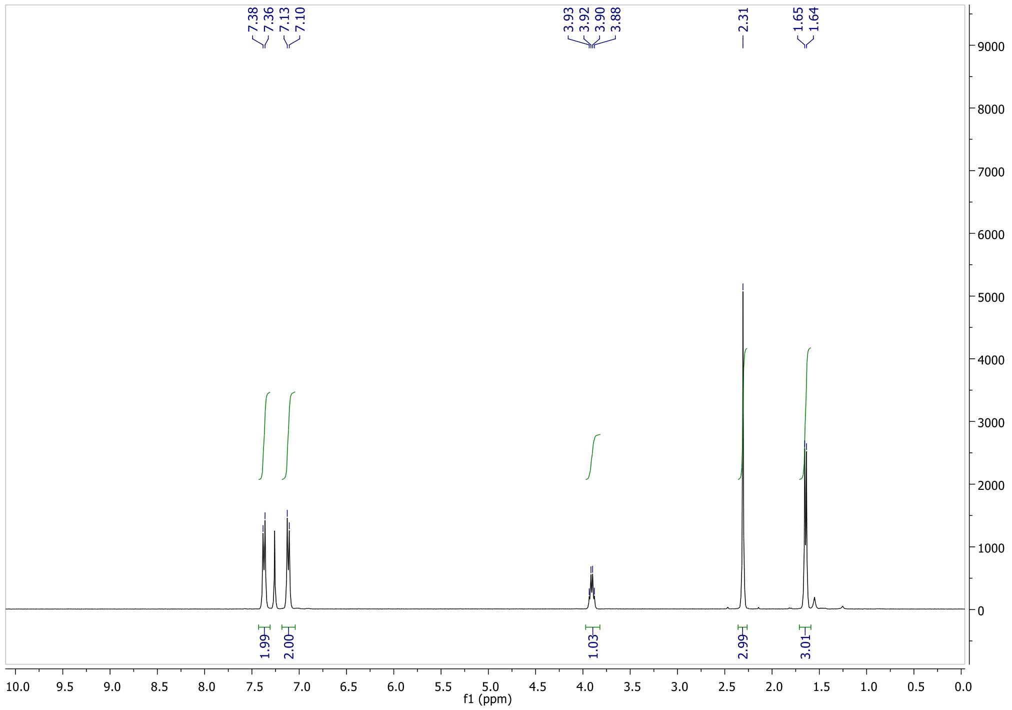
7.38
7.36
7.13
7.10
3.93
3.92
3.90
3.88
2.31
1.65
1.64
1.99
2.00
1.03
2.99
3.01
f1 (ppm)

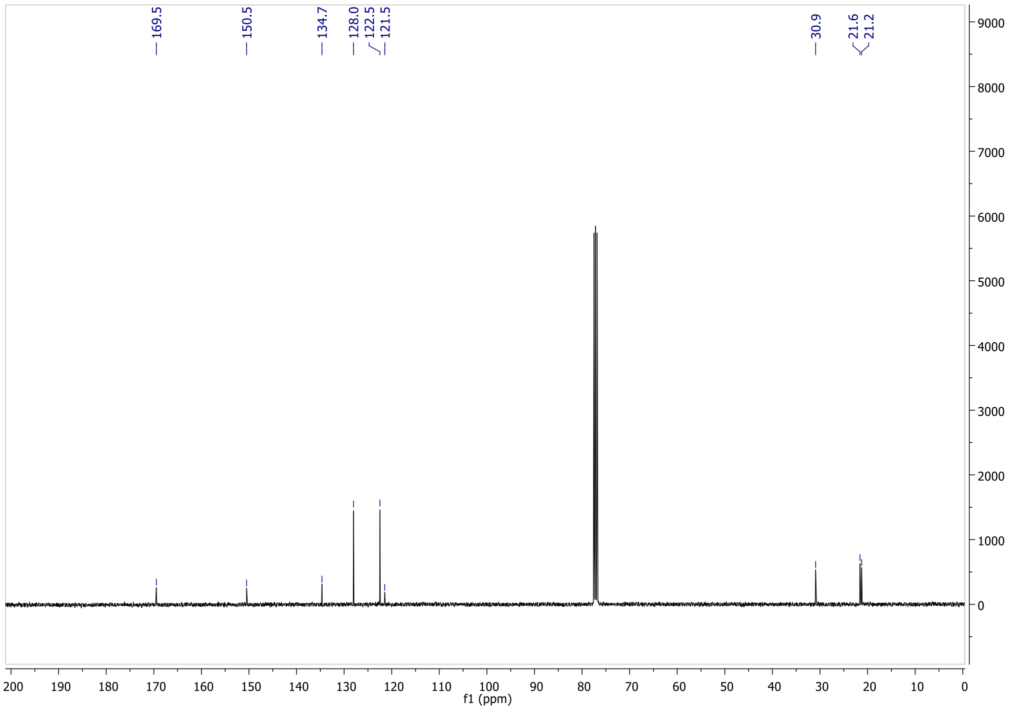
169.5
150.5
134.7
128.0
122.5
121.5
30.9
21.6
21.2
f1 (ppm)

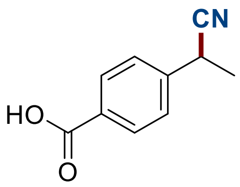
CN
HO
O

7

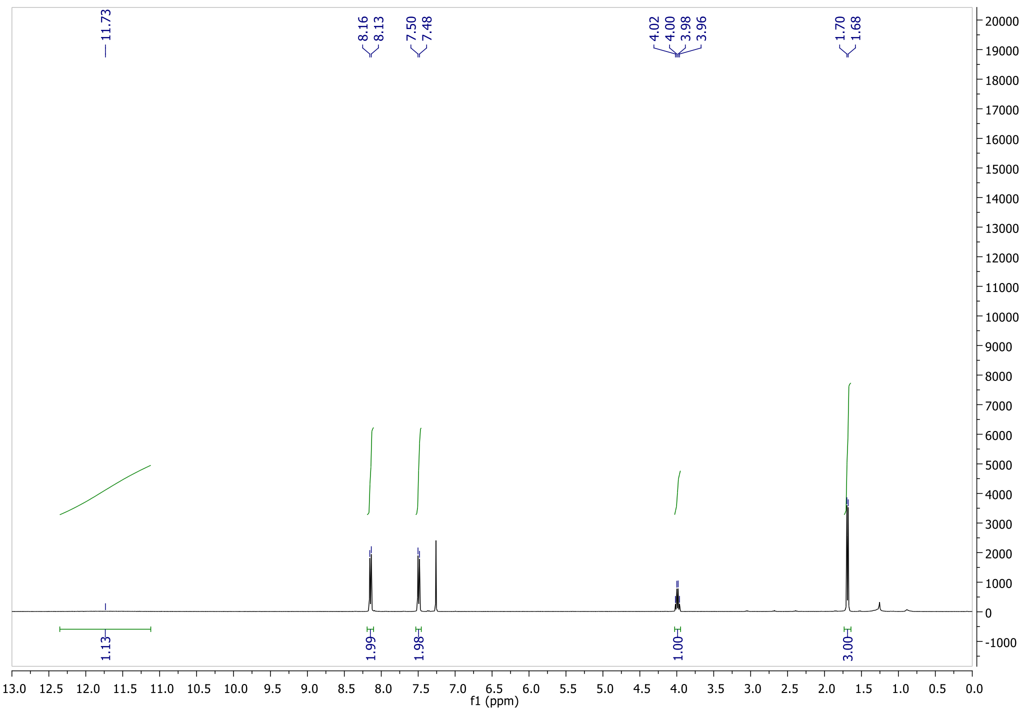
11.73
8.16
8.13
7.50
7.48
4.02
4.00
3.98
3.96
1.70
1.68
1.13
1.99
1.98
1.00
3.00
f1 (ppm)

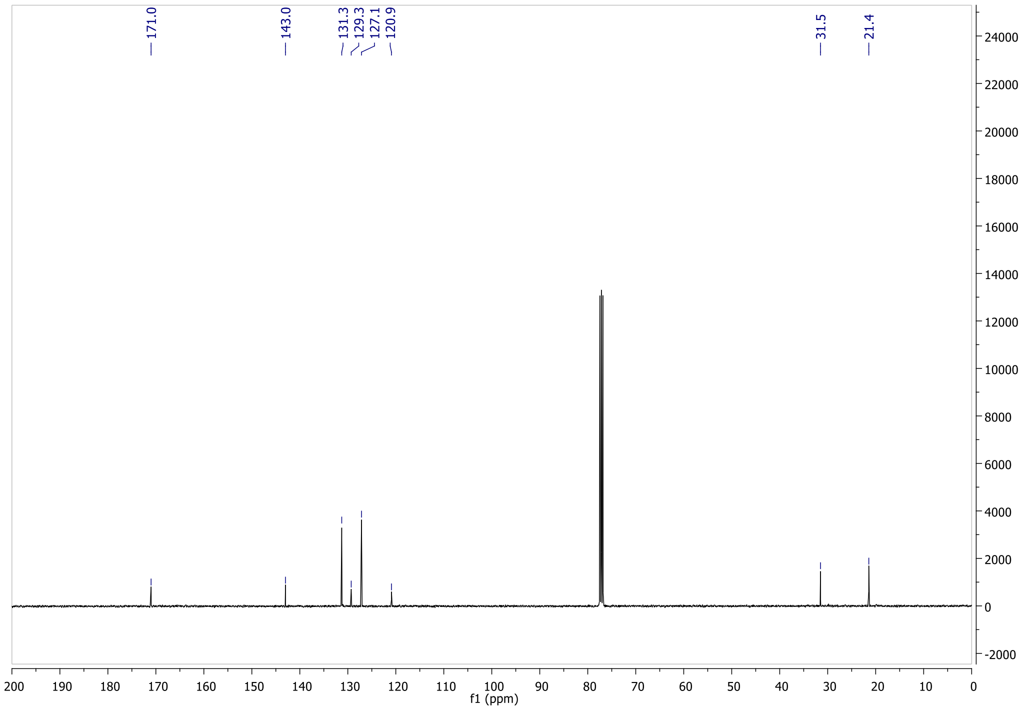
171.0
143.0
131.3
129.3
127.1
120.9
31.5
21.4
f1 (ppm)

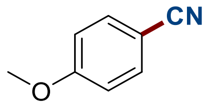
CN
O

8

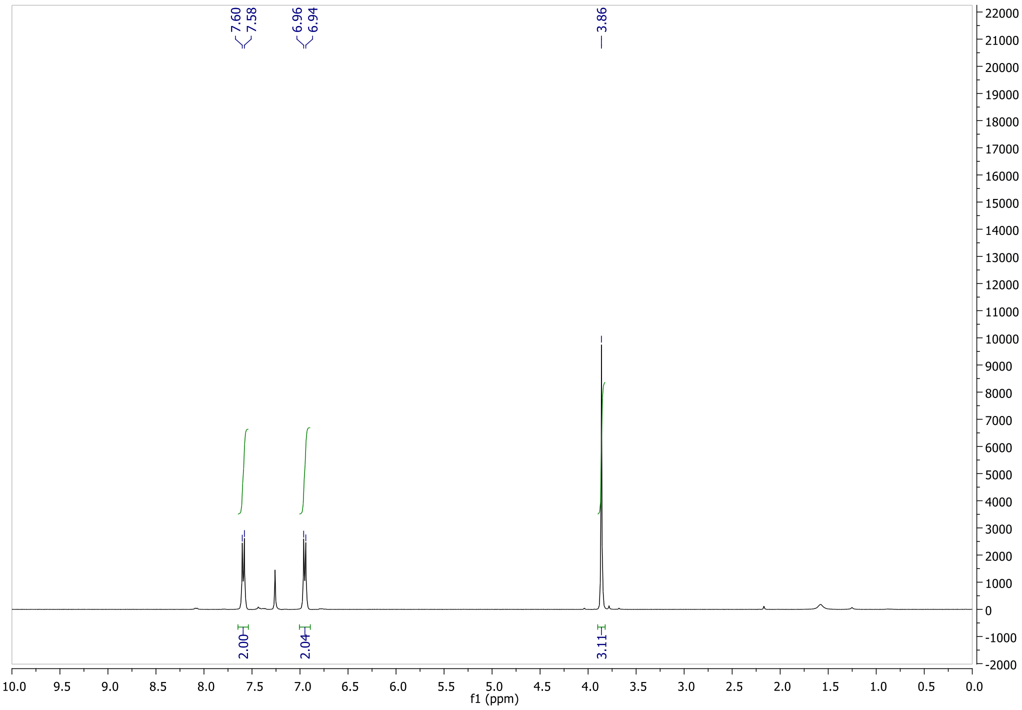
7.60
7.58
6.96
6.94
3.86
2.00
2.04
3.11
f1 (ppm)

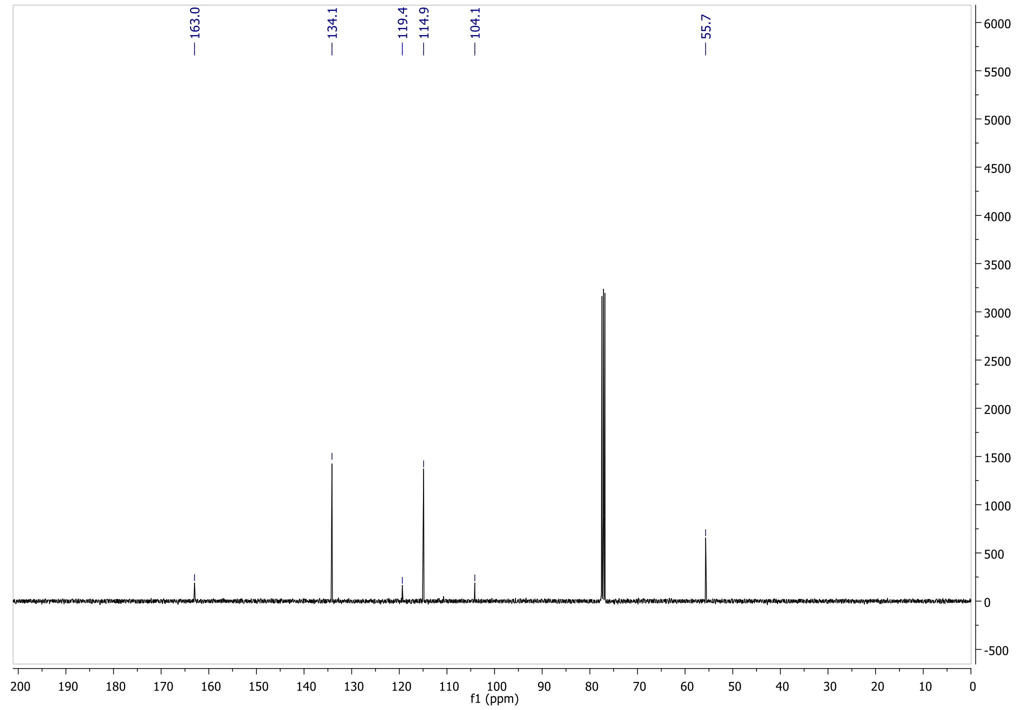
163.0
134.1
119.4
114.9
104.1
55.7
f1 (ppm)

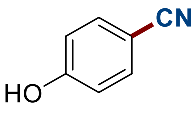
CN
HO

9

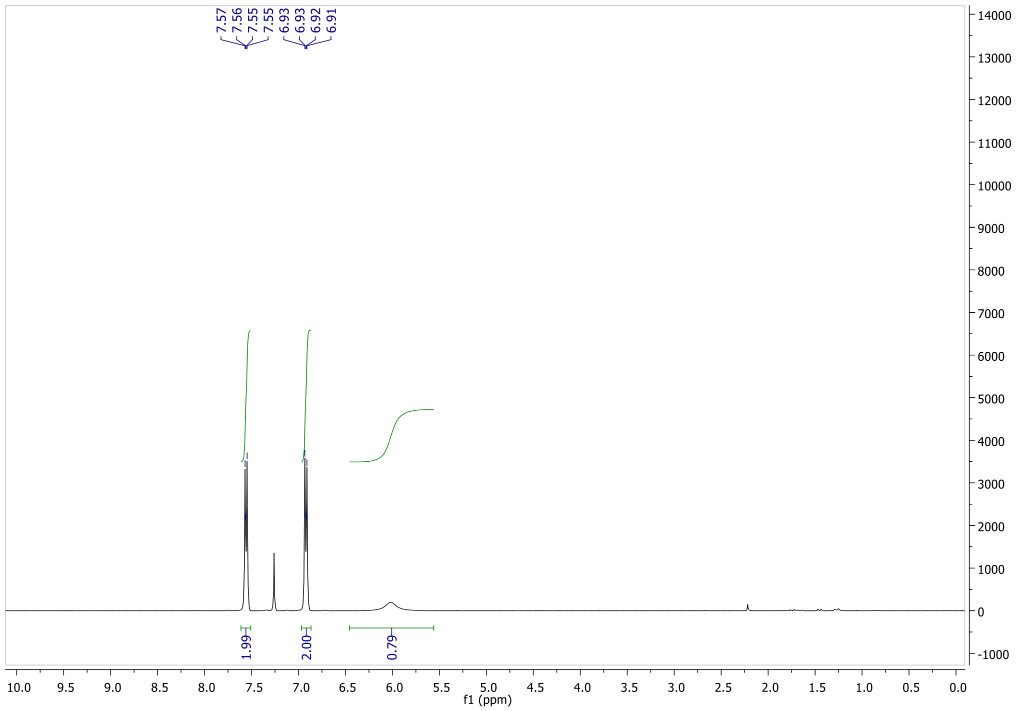
7.57
7.56
7.55
7.55
6.93
6.93
6.92
6.91
1.99
2.00
0.79
f1 (ppm)

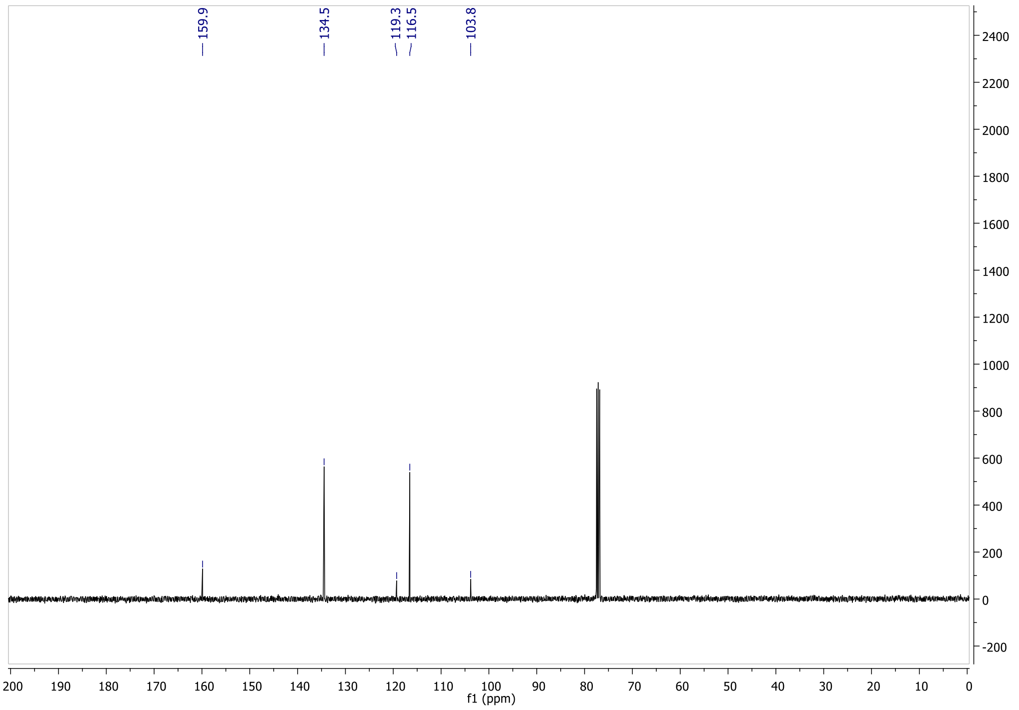
159.9
134.5
119.3
116.5
103.8
f1 (ppm)

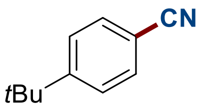

10

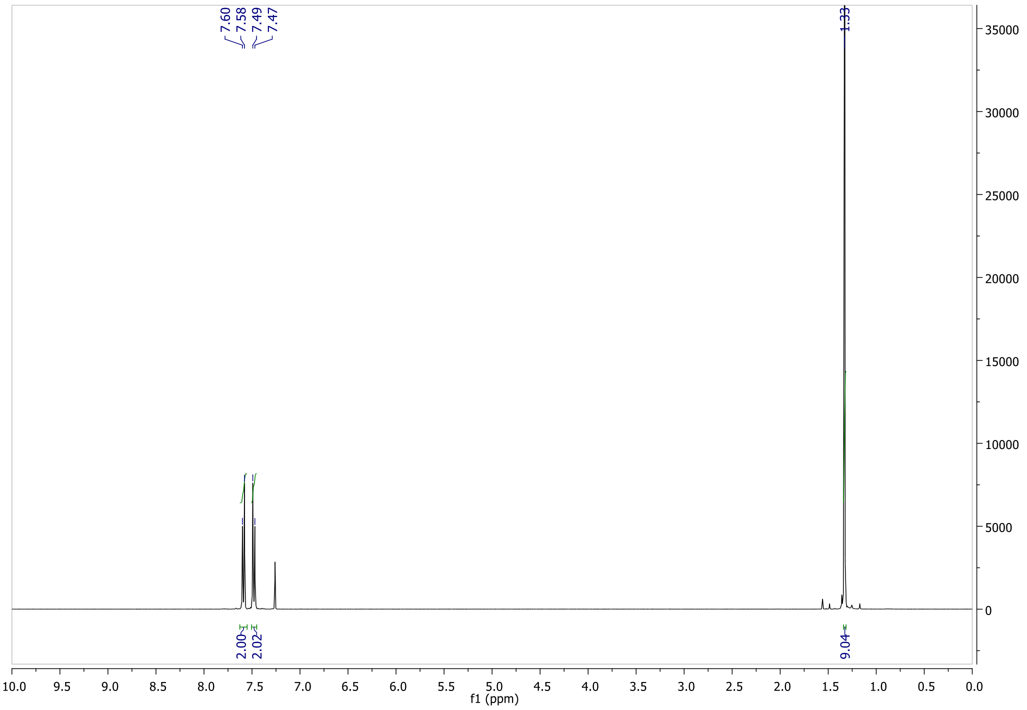

7.60
7.58
7.49
7.47
1.33
2.00
2.02
9.04
f1 (ppm)

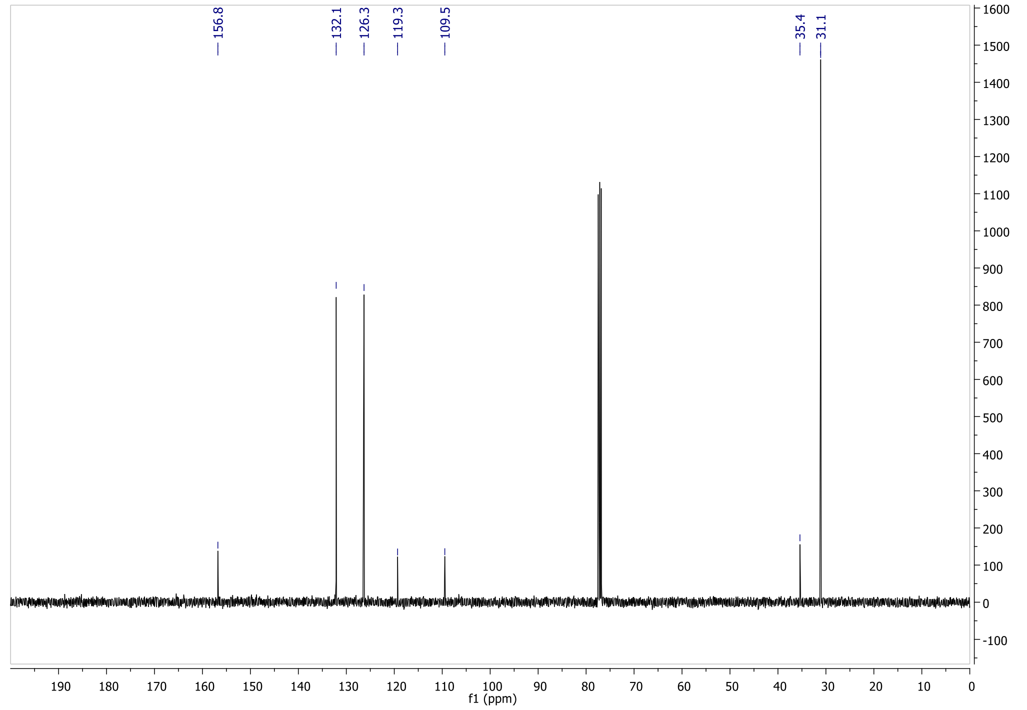

156.8
132.1
126.3
119.3
109.5
35.4
31.1
f1 (ppm)

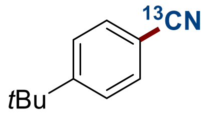

10-^{13}C

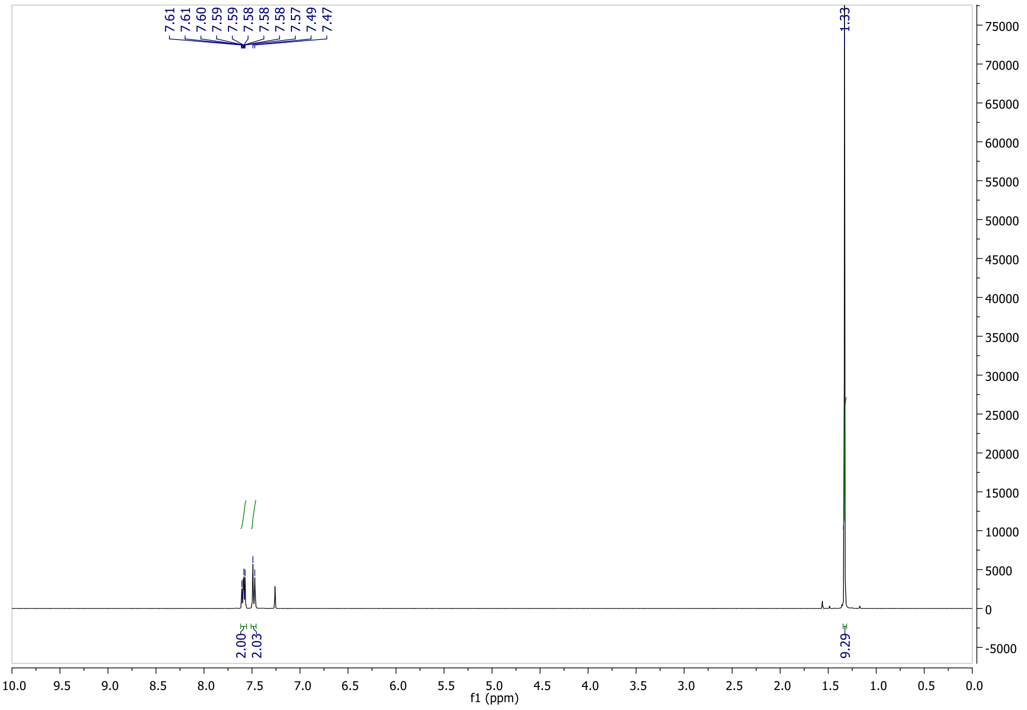

7.61
7.61
7.60
7.59
7.59
7.58
7.58
7.58
7.57
7.49
7.47
1.33
2.00
2.03
9.29
f1 (ppm)

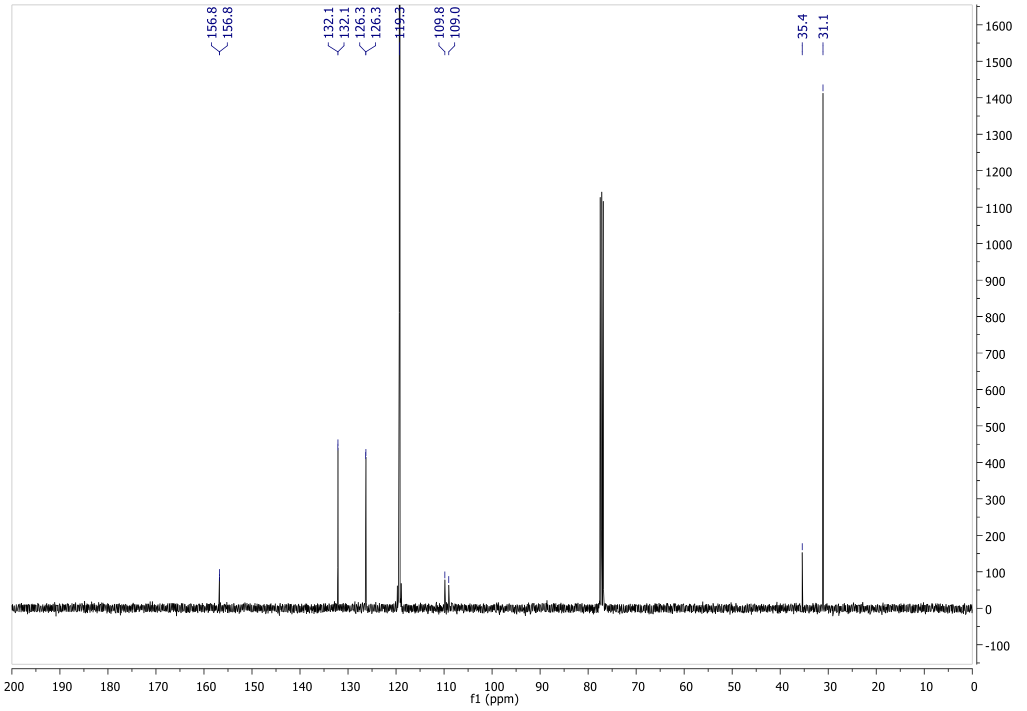

156.8
156.8
132.1
132.1
126.3
126.3
119.3
109.8
109.0
35.4
31.1
f1 (ppm)

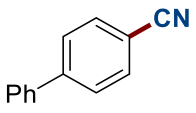
CN
Ph

11

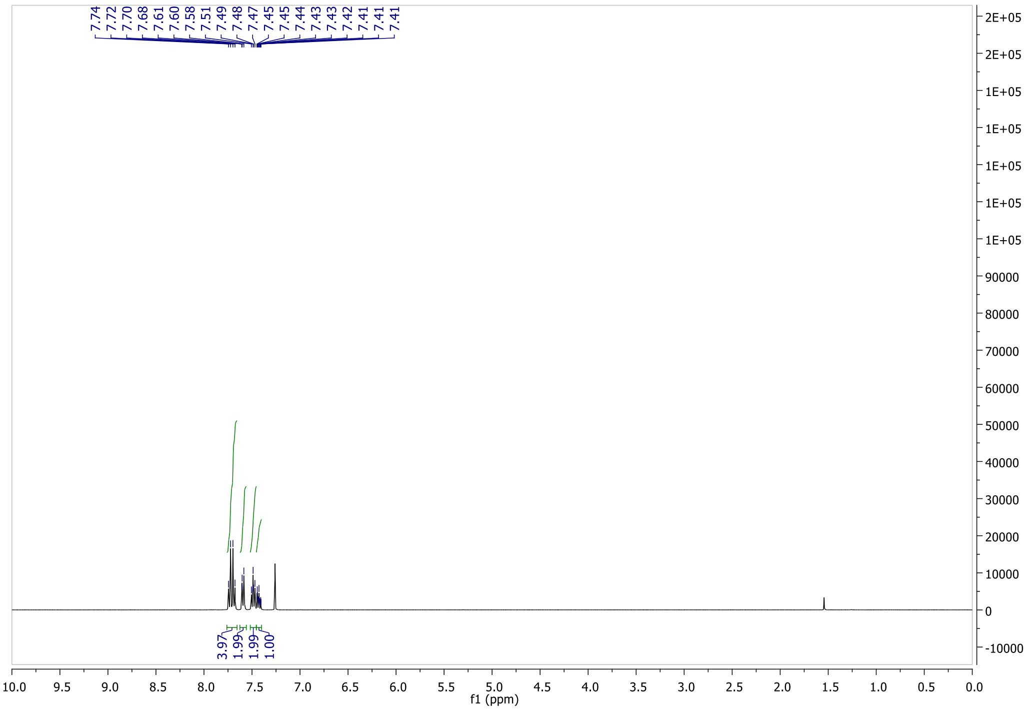
7.74
7.72
7.70
7.68
7.61
7.60
7.58
7.51
7.49
7.48
7.47
7.45
7.45
7.44
7.43
7.43
7.42
7.41
7.41
7.41
3.97
1.99
1.99
1.00
f1 (ppm)

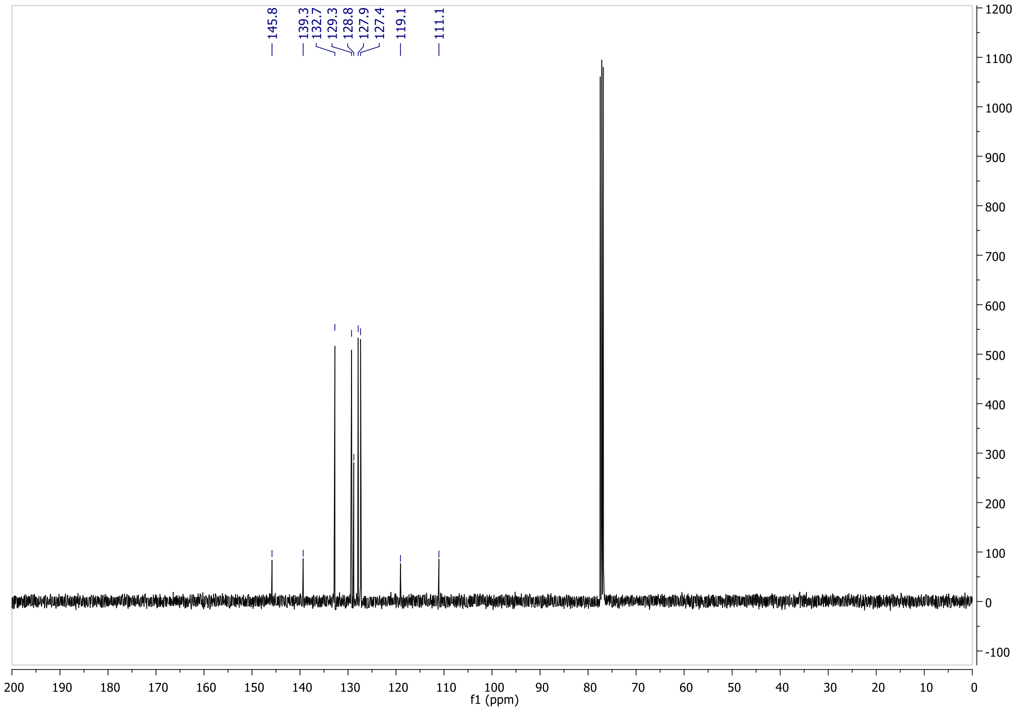
145.8
139.3
132.7
129.3
128.8
127.9
127.4
119.1
111.1
f1 (ppm)

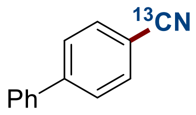
13CN
Ph

11-^{13}C

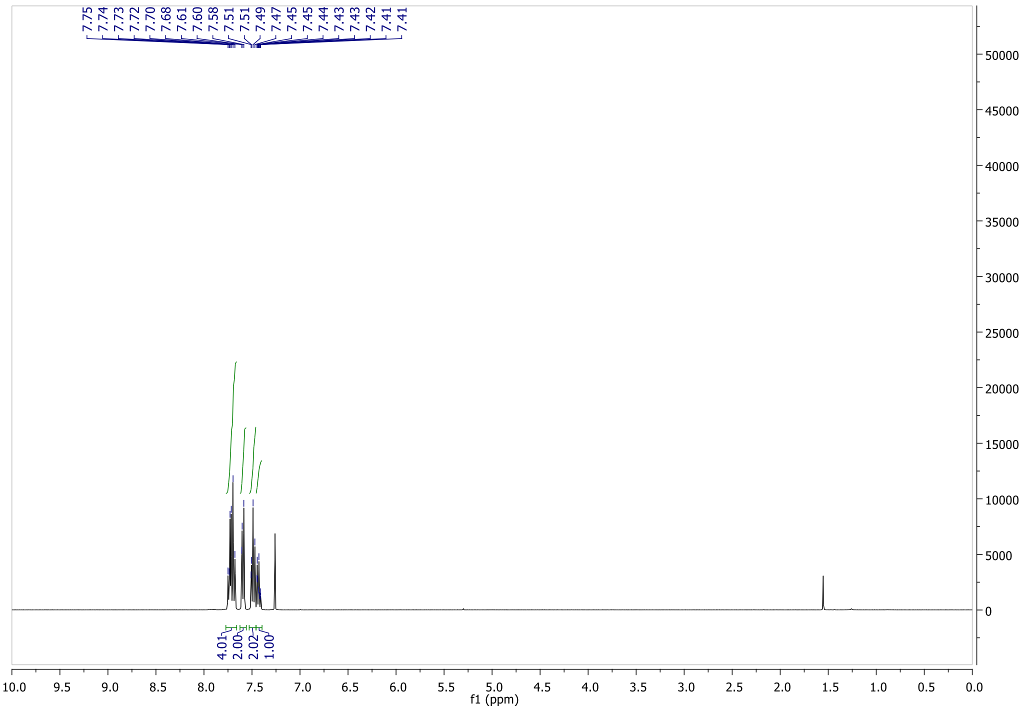
7.75
7.74
7.73
7.72
7.70
7.68
7.61
7.60
7.58
7.51
7.51
7.49
7.47
7.45
7.45
7.44
7.43
7.43
7.42
7.41
7.41
4.01
2.00
2.02
1.00
f1 (ppm)

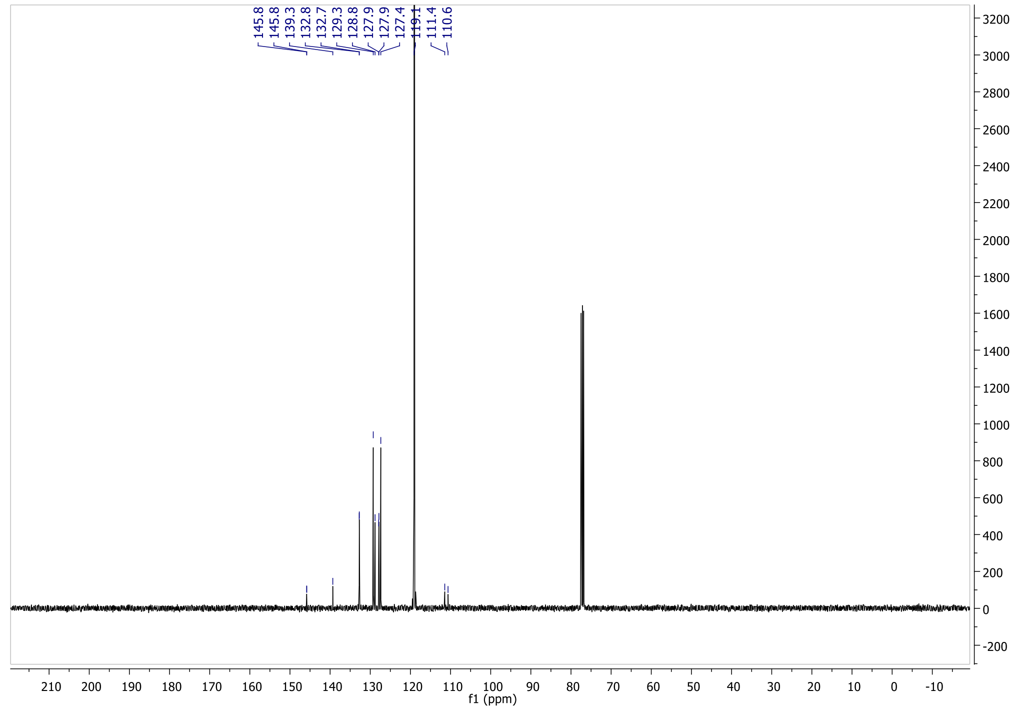
145.8
145.8
139.3
132.8
132.7
129.3
128.8
127.9
127.9
127.4
119.1
111.4
110.6
f1 (ppm)

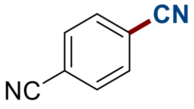

12

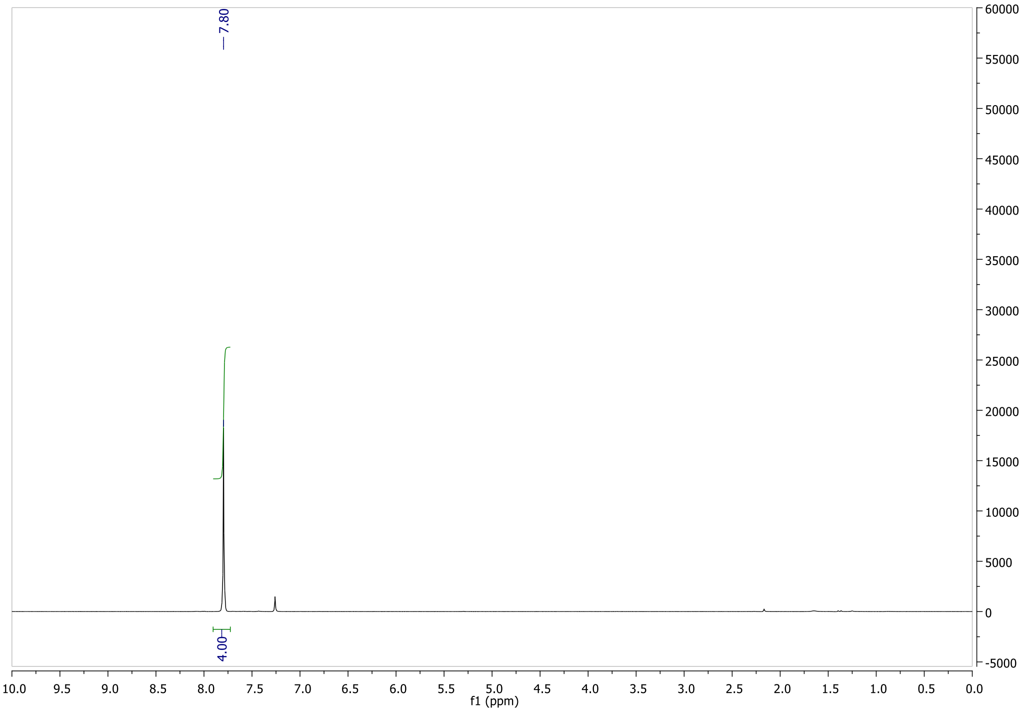

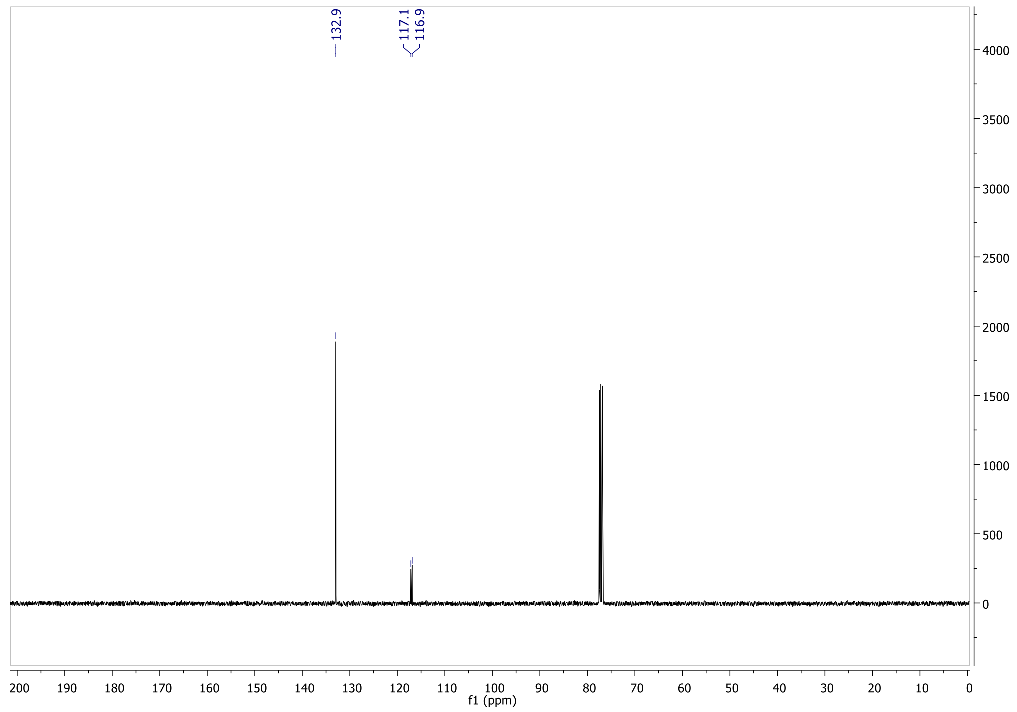

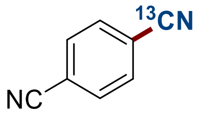

12-^{13}C

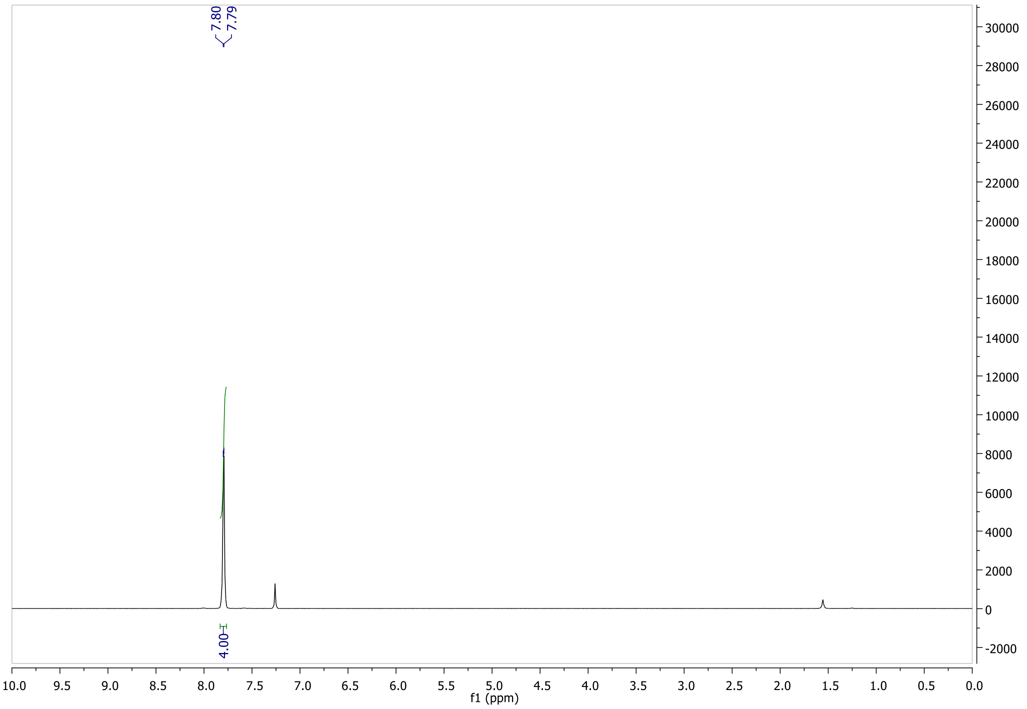

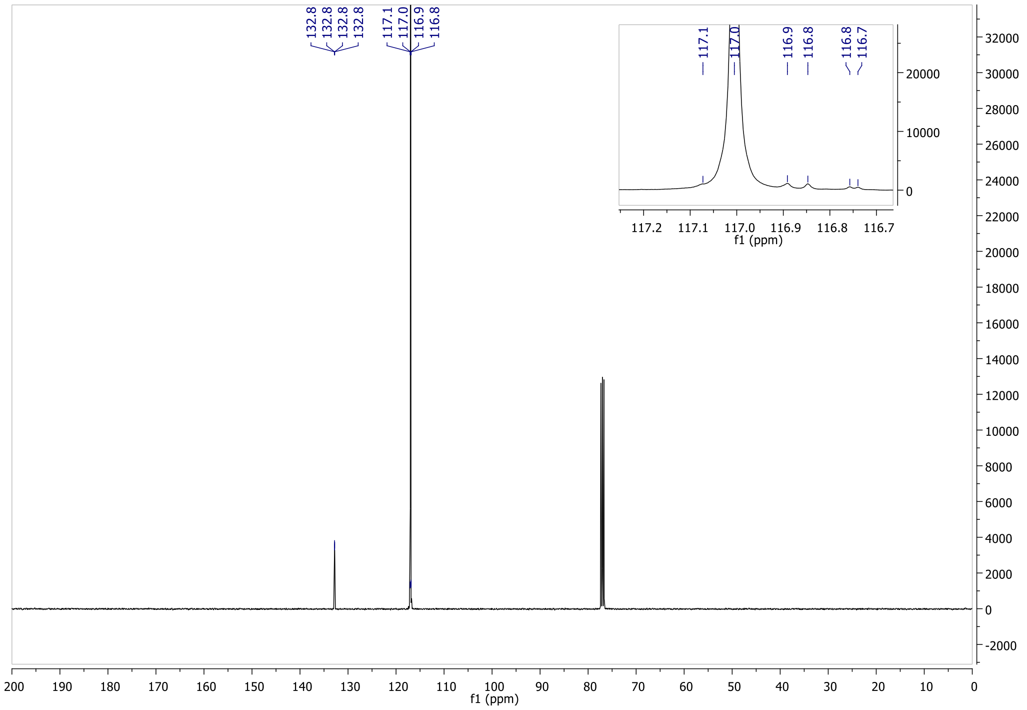

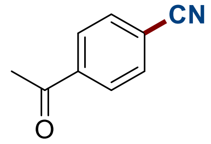
CN
O

13

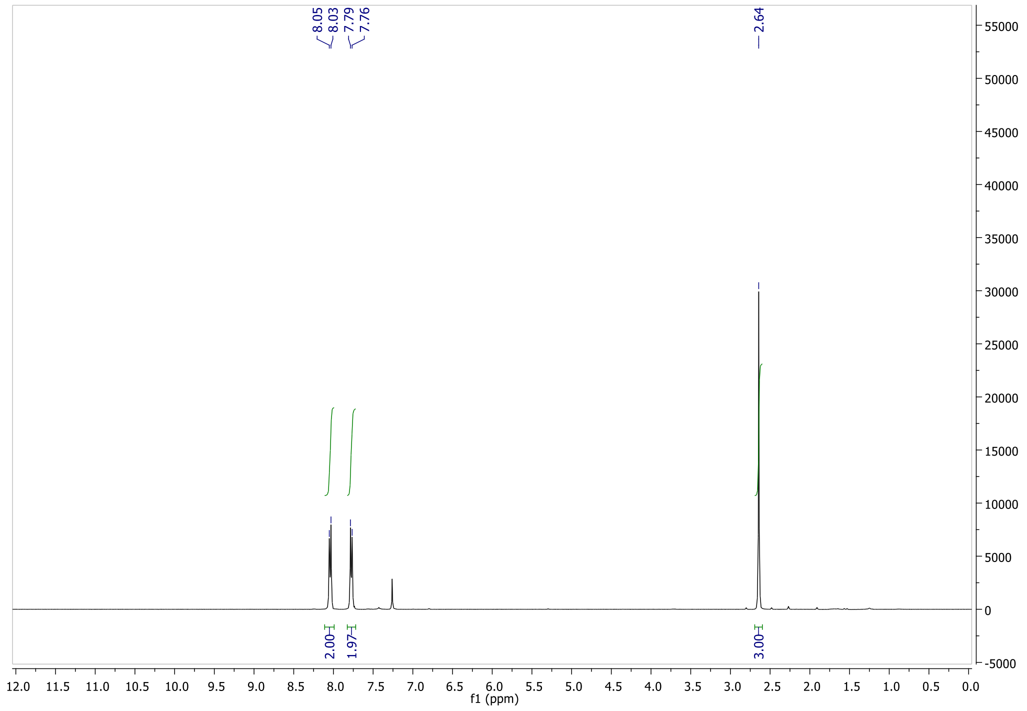
8.05
8.03
7.79
7.76
2.64
2.00
1.97
3.00
f1 (ppm)

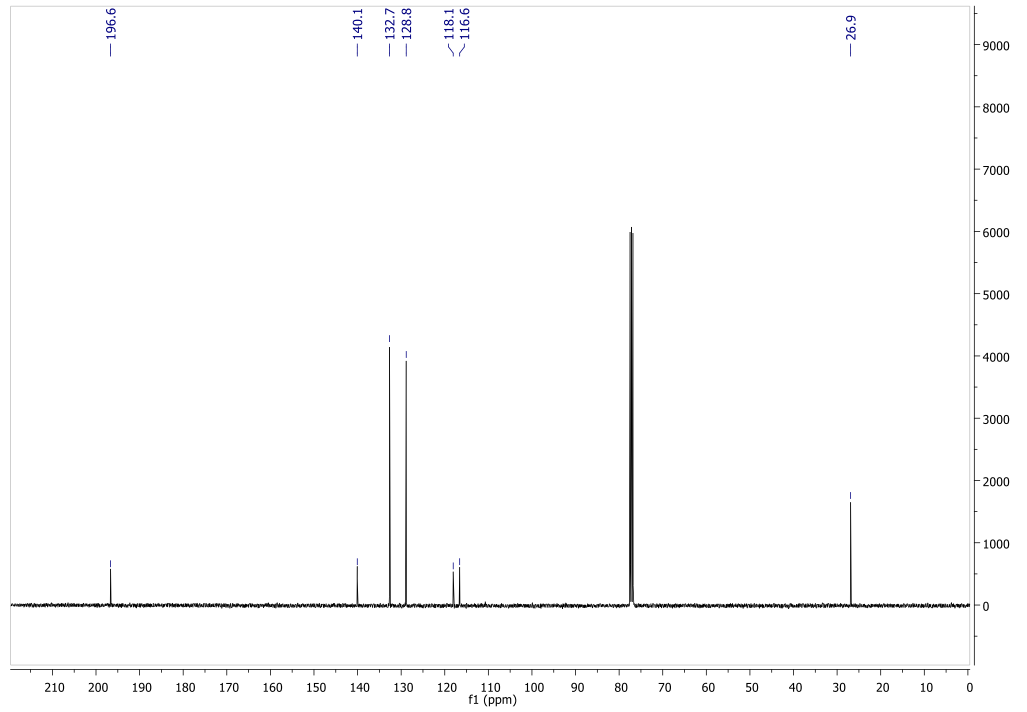
196.6
140.1
132.7
128.8
118.1
116.6
26.9
f1 (ppm)

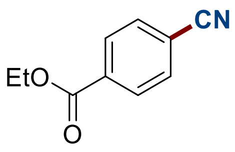
CN
EtO
O

14

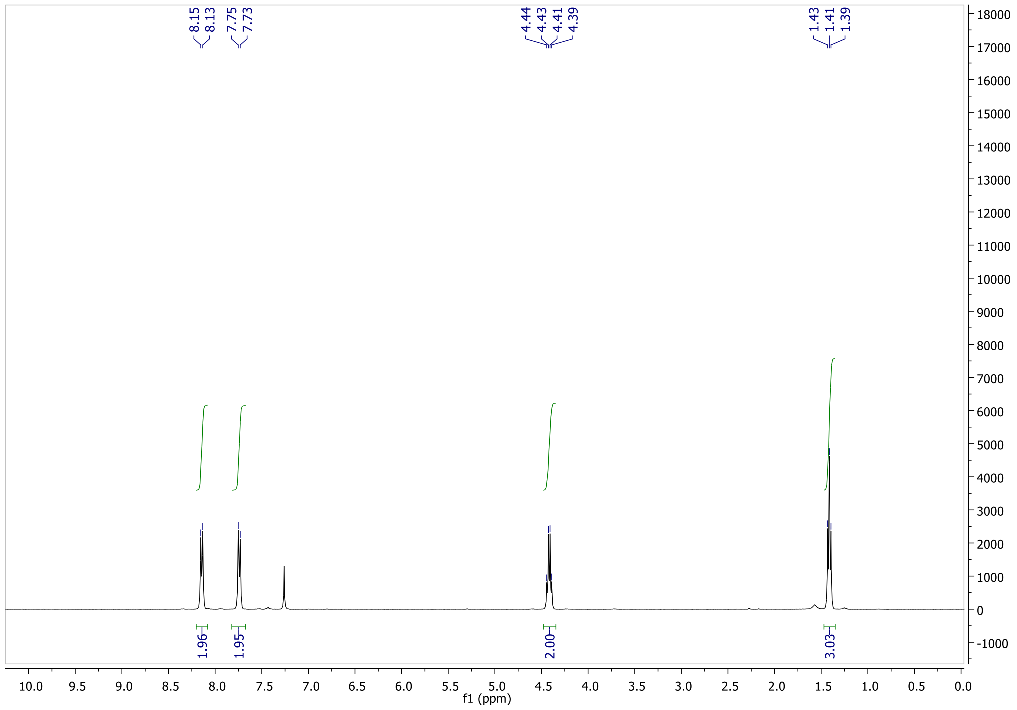
8.15
8.13
7.75
7.73
4.44
4.43
4.41
4.39
1.43
1.41
1.39
1.96
1.95
2.00
3.03
f1 (ppm)

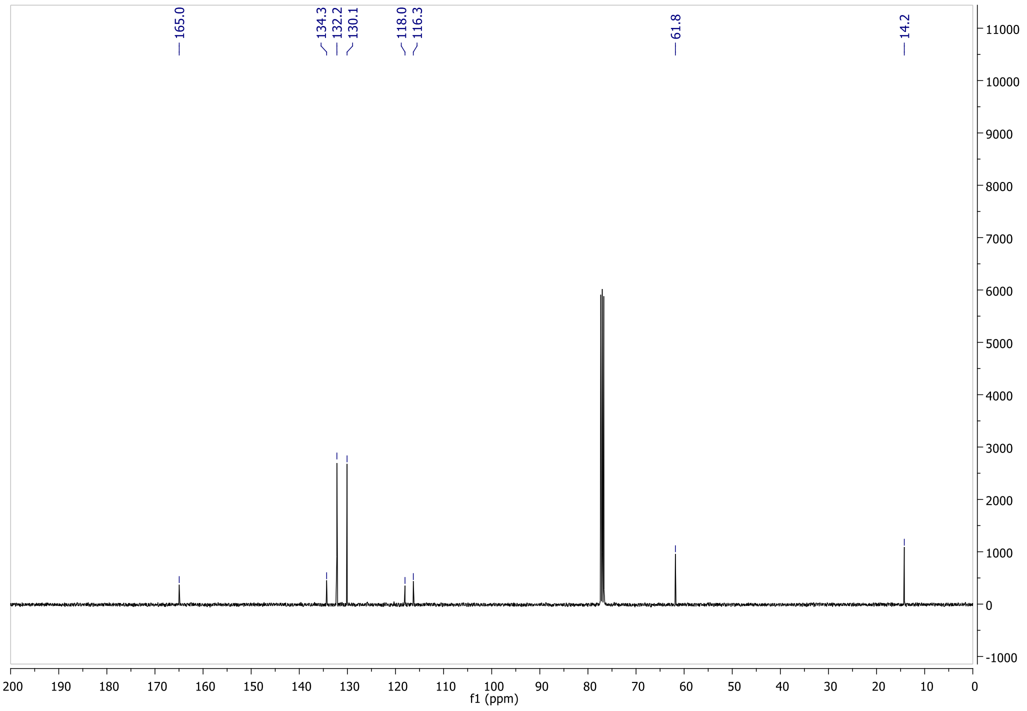
165.0
134.3
132.2
130.1
118.0
116.3
61.8
14.2
f1 (ppm)

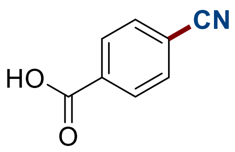
CN
HO
O

15

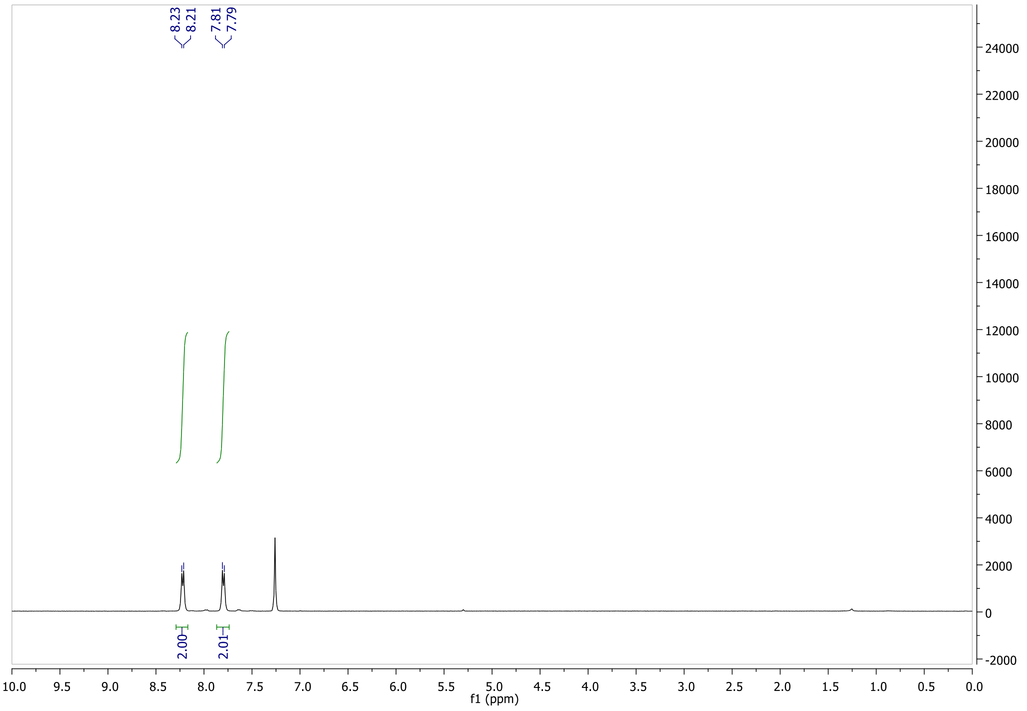
8.23
8.21
7.81
7.79
2.00
2.01
f1 (ppm)

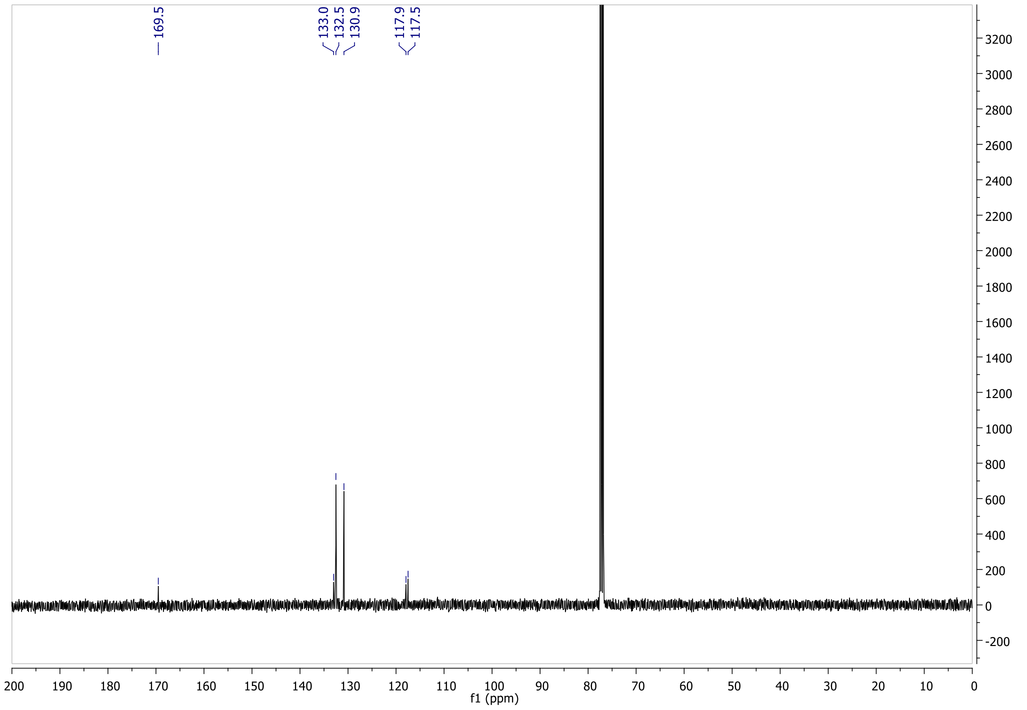
169.5
133.0
132.5
130.9
117.9
117.5
f1 (ppm)

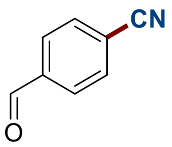

16

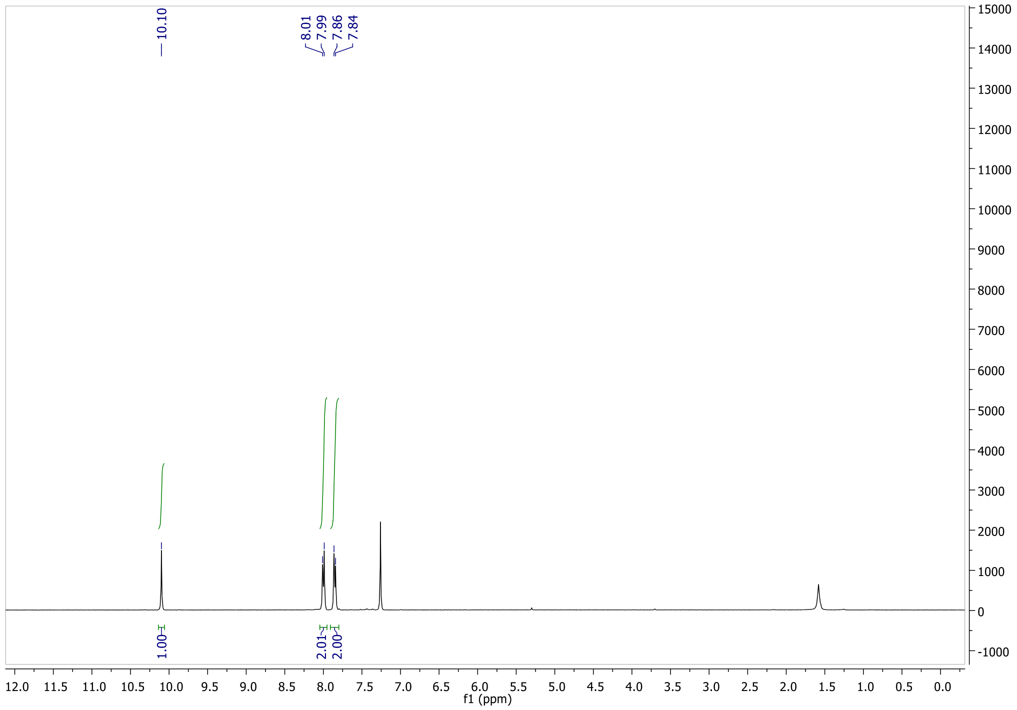

10.10
8.01
7.99
7.86
7.84
1.00
2.01
2.00
f1 (ppm)

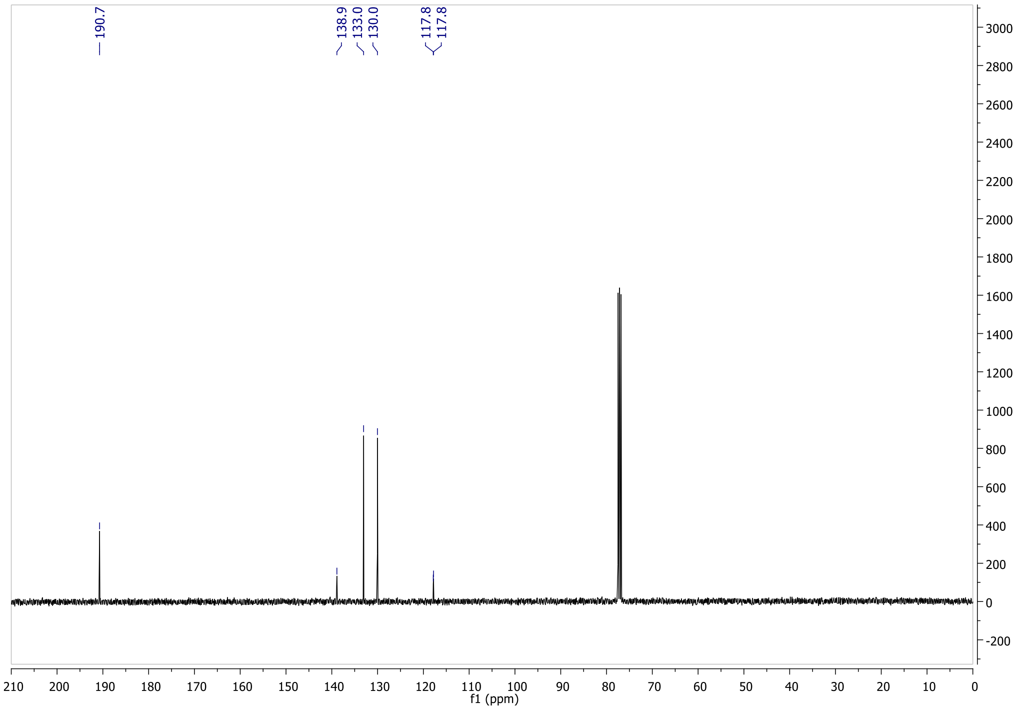

190.7
138.9
133.0
130.0
117.8
117.8
f1 (ppm)

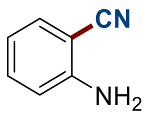

17

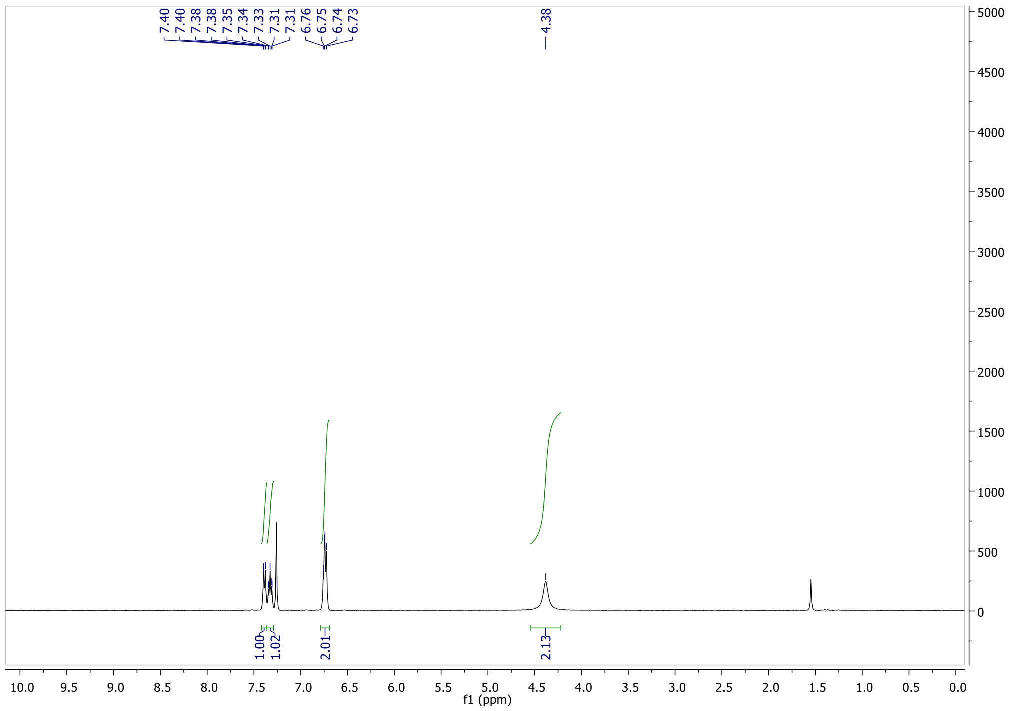

7.40
7.40
7.38
7.38
7.35
7.34
7.33
7.31
7.31
6.76
6.75
6.74
6.73
4.38
1.00
1.02
2.01
2.13
f1 (ppm)

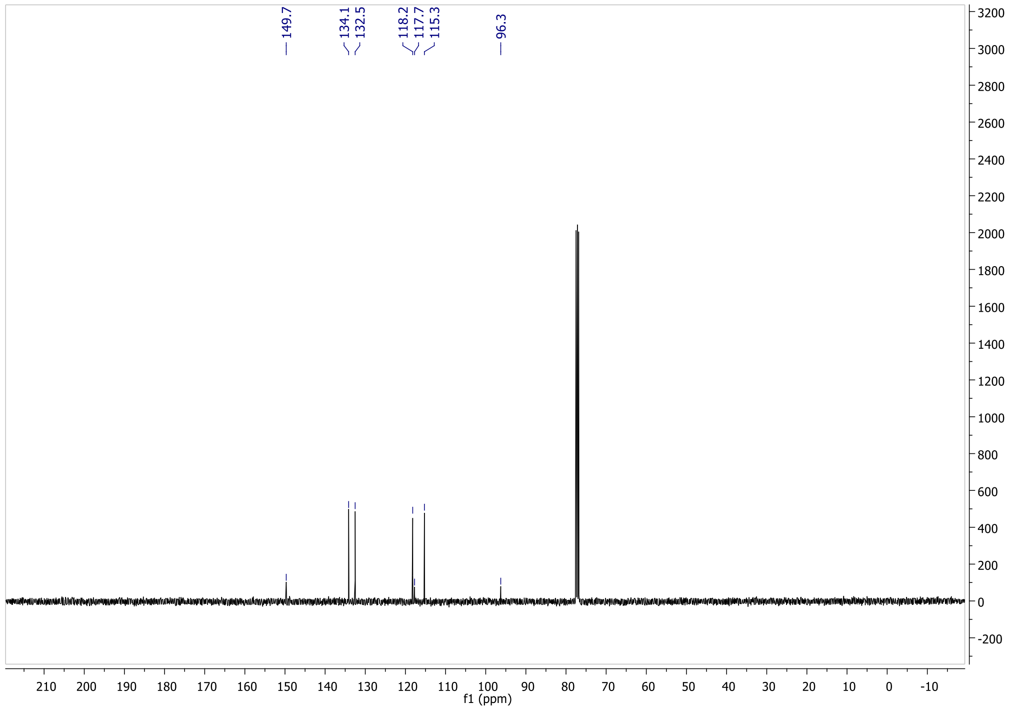

149.7
134.1
132.5
118.2
117.7
115.3
96.3
f1 (ppm)

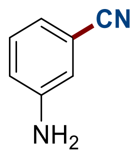
CN
NH2

18

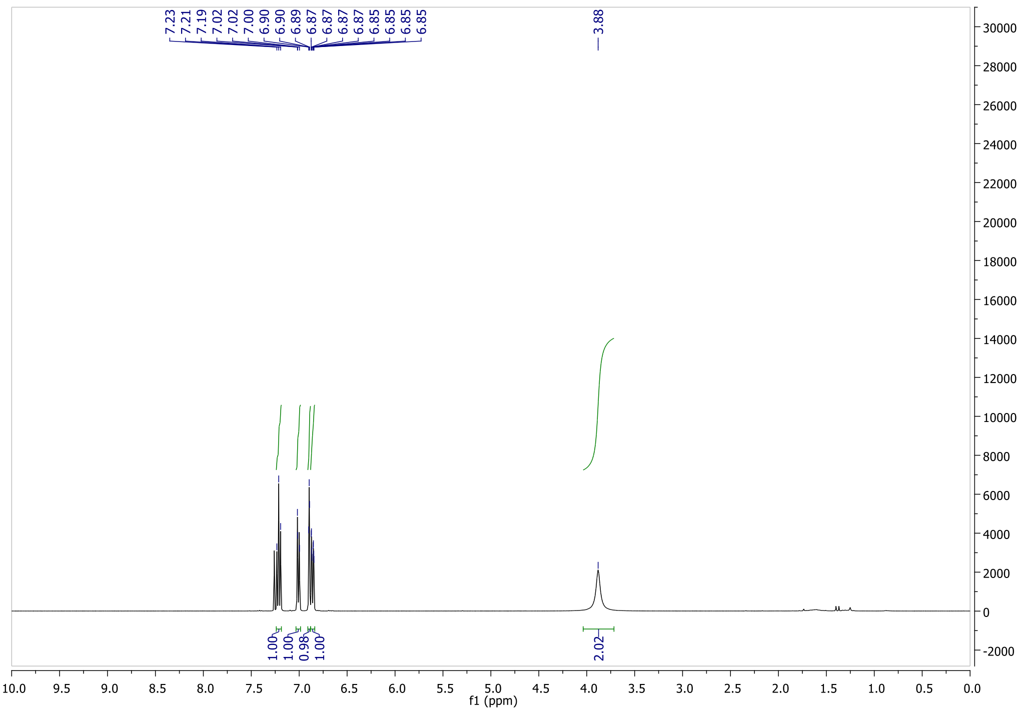
7.23
7.21
7.19
7.02
7.02
7.00
6.90
6.90
6.89
6.87
6.87
6.87
6.85
6.85
6.85
6.85
3.88
1.00
1.00
0.98
1.00
2.02
f1 (ppm)

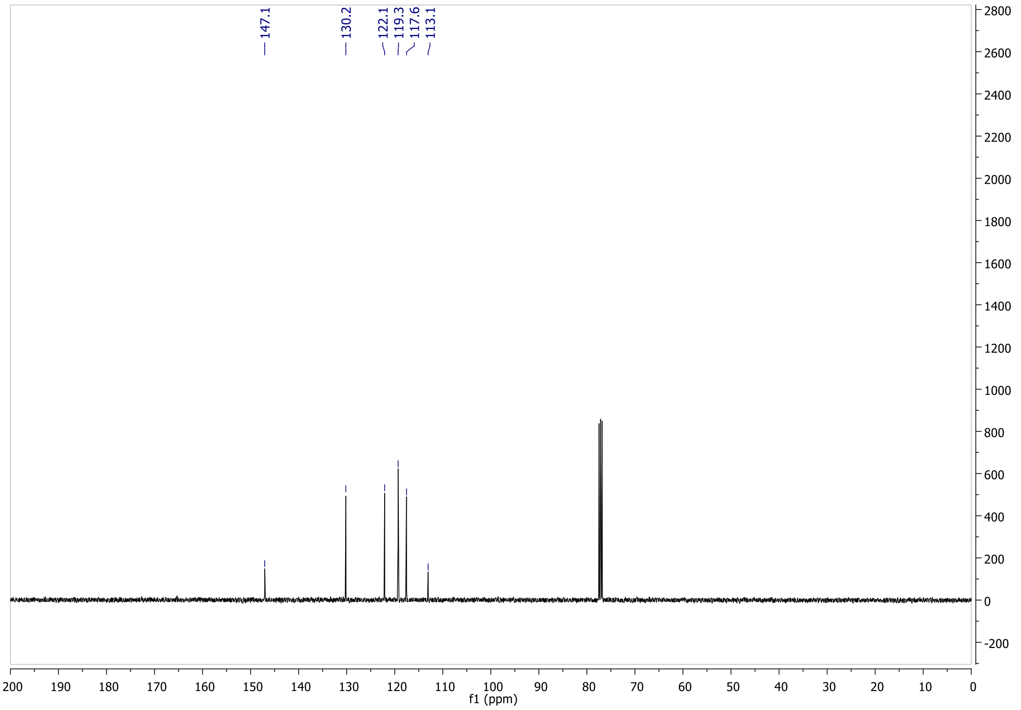
147.1
130.2
122.1
119.3
117.6
113.1
f1 (ppm)

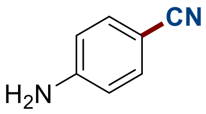
CN
H2N

19

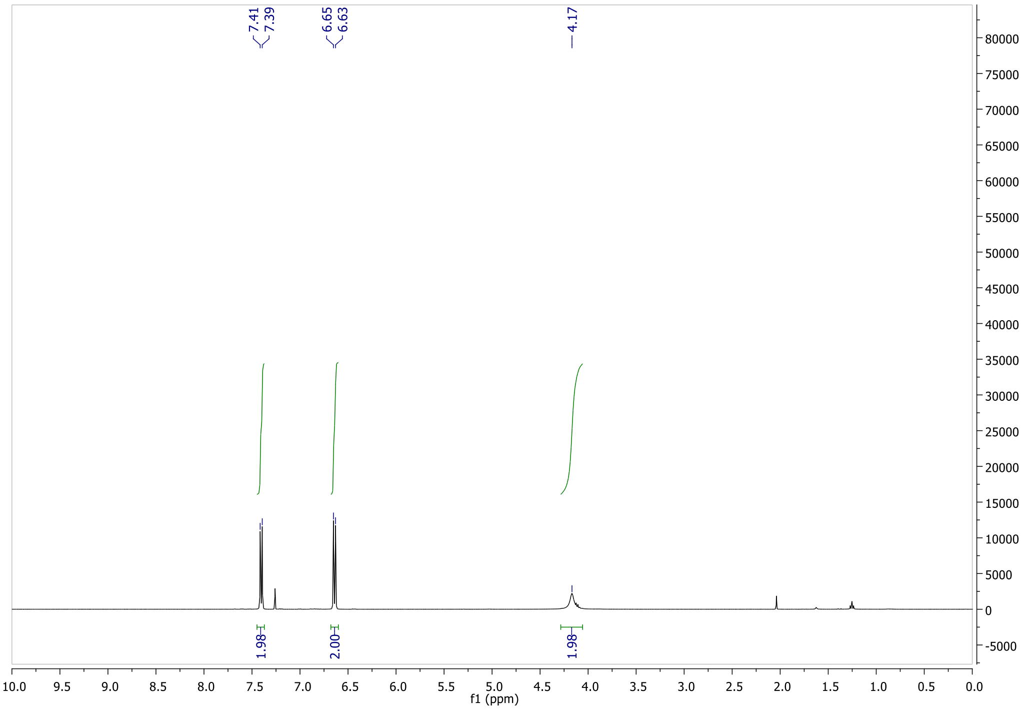
7.41
7.39
6.65
6.63
4.17
1.98
2.00
1.98
f1 (ppm)

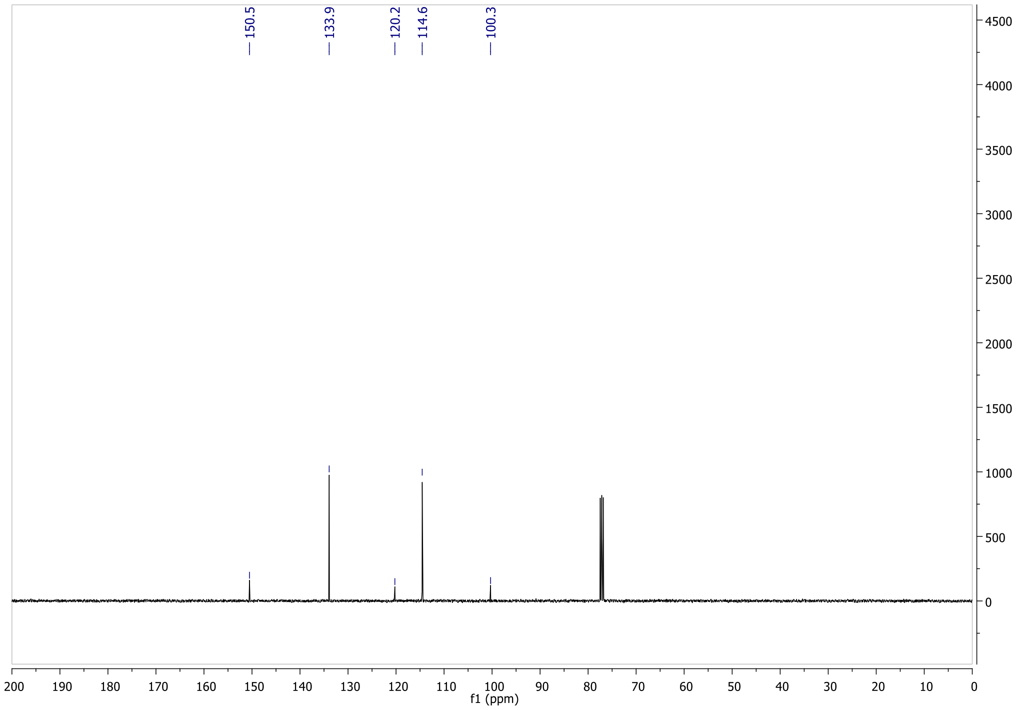
150.5
133.9
120.2
114.6
100.3
f1 (ppm)

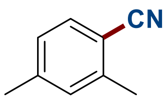
CN

20

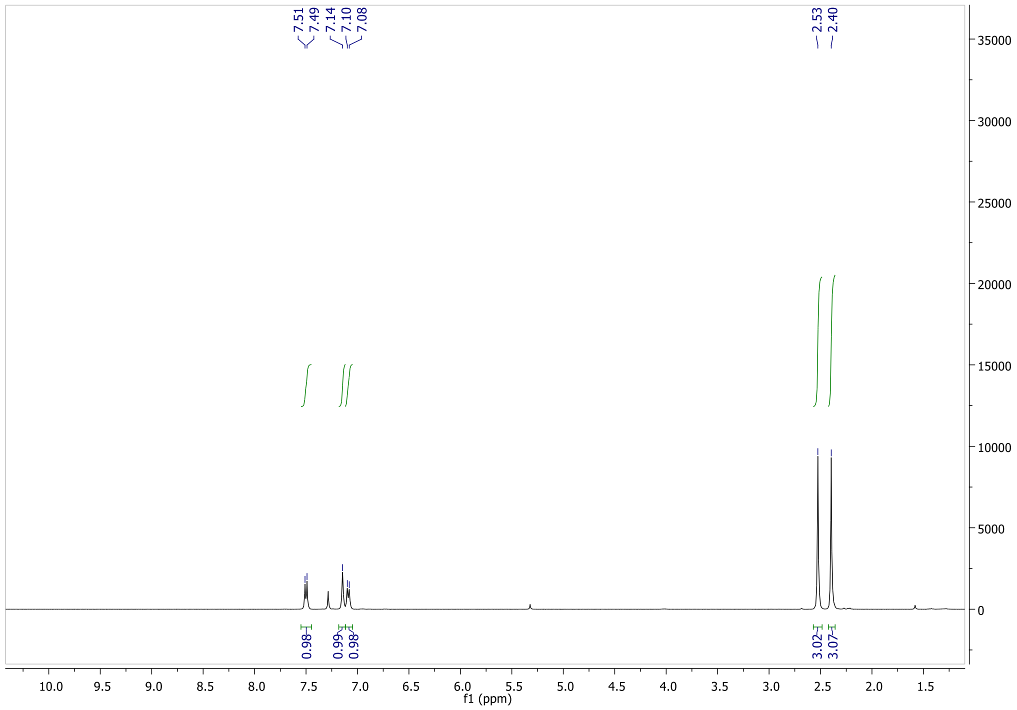
7.51
7.49
7.14
7.10
7.08
2.53
2.40
0.98
0.99
0.98
3.02
3.07
f1 (ppm)

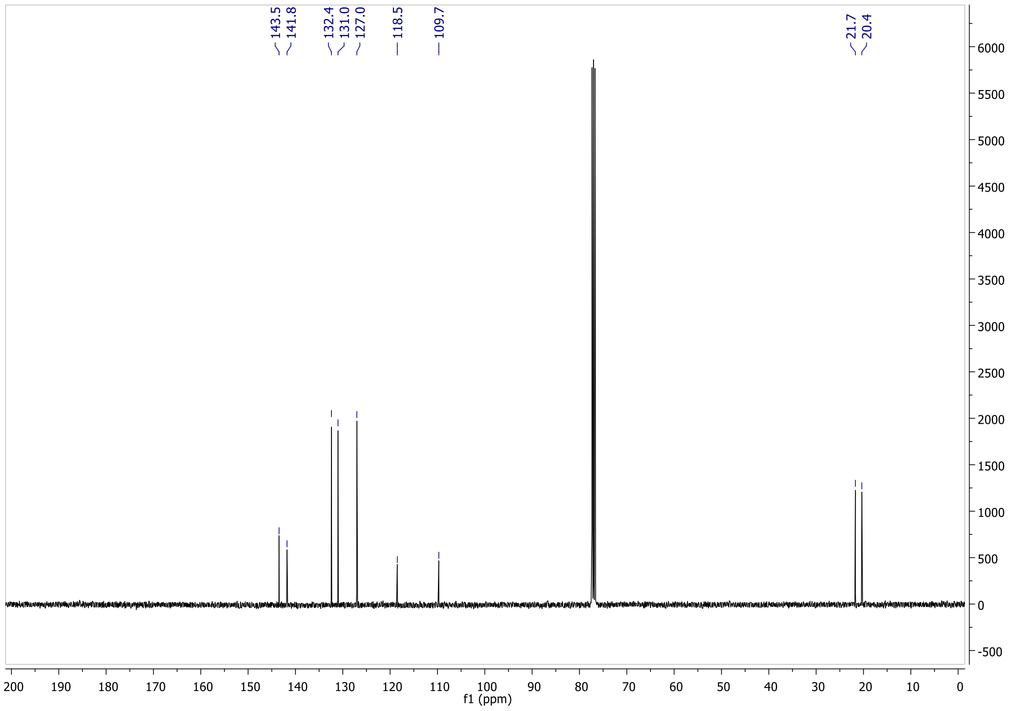
143.5
141.8
132.4
131.0
127.0
118.5
109.7
21.7
20.4
f1 (ppm)

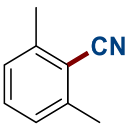

21

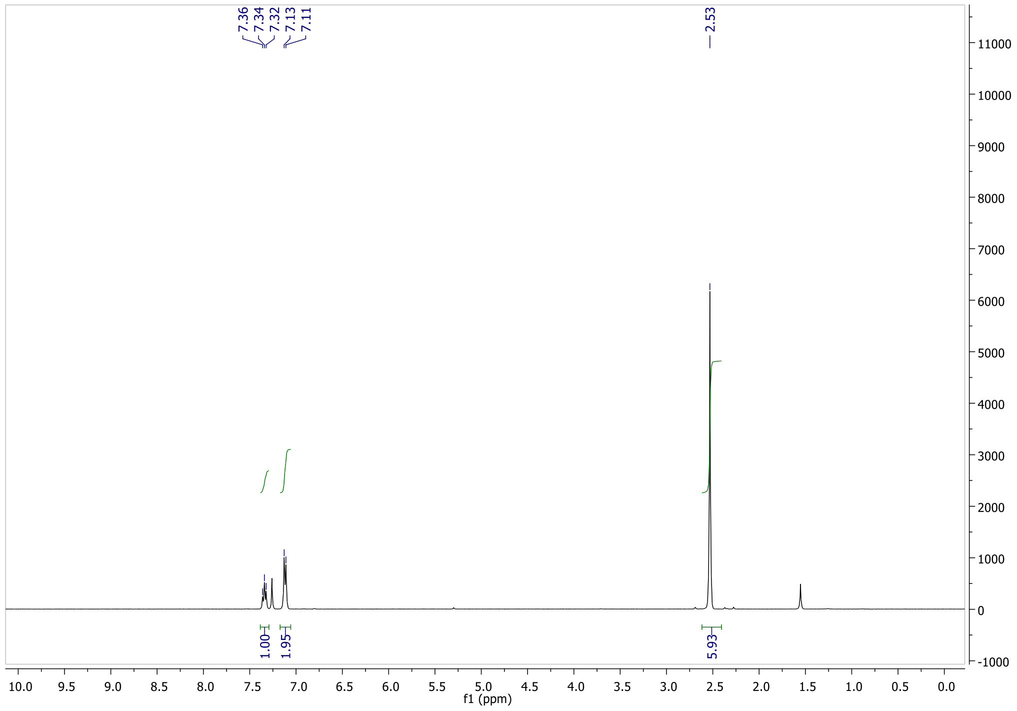
7.36
7.34
7.32
7.13
7.11
2.53
1.00
1.95
5.93
f1 (ppm)

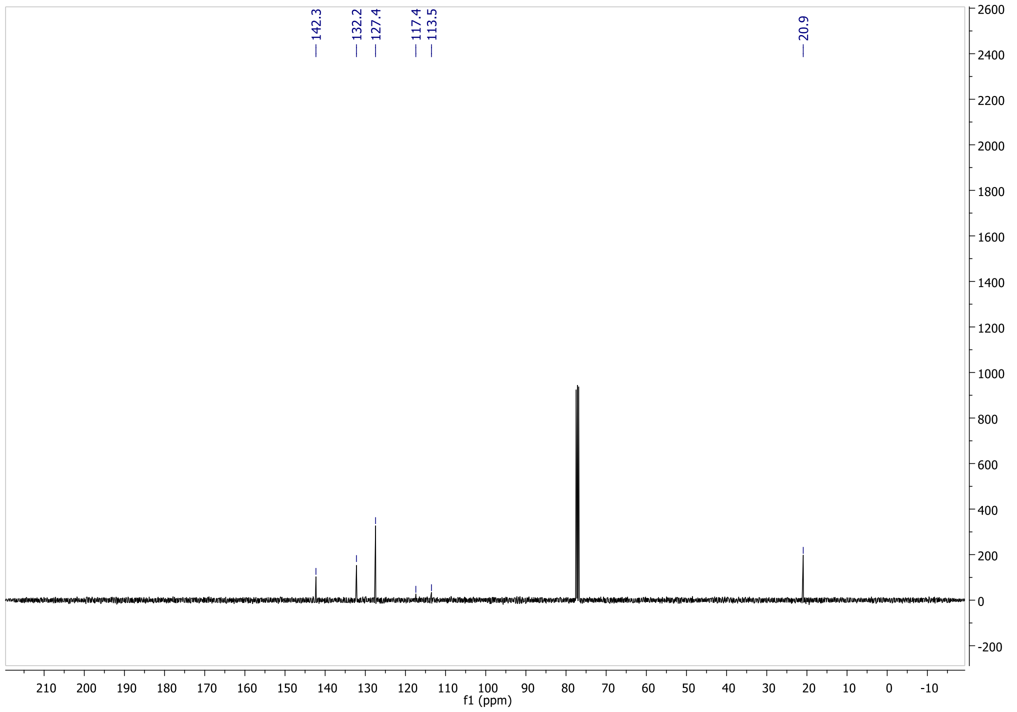
142.3
132.2
127.4
117.4
113.5
20.9
f1 (ppm)

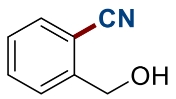
CN
OH

22

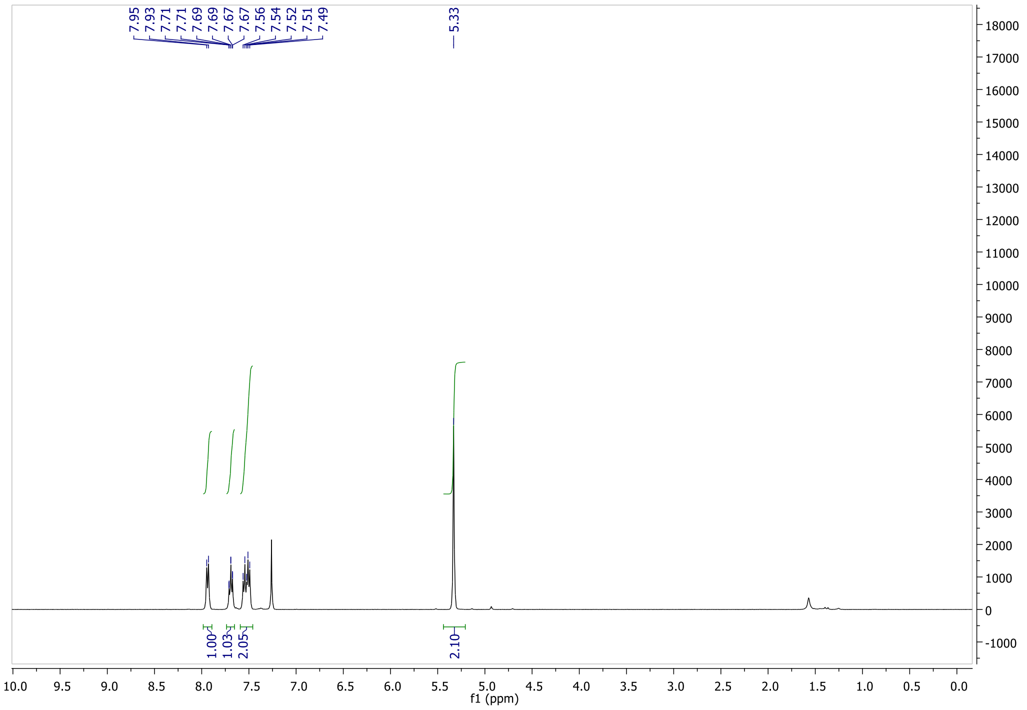
7.95
7.93
7.71
7.71
7.69
7.69
7.67
7.67
7.56
7.54
7.52
7.51
7.49
5.33
1.00
1.03
2.05
2.10
f1 (ppm)

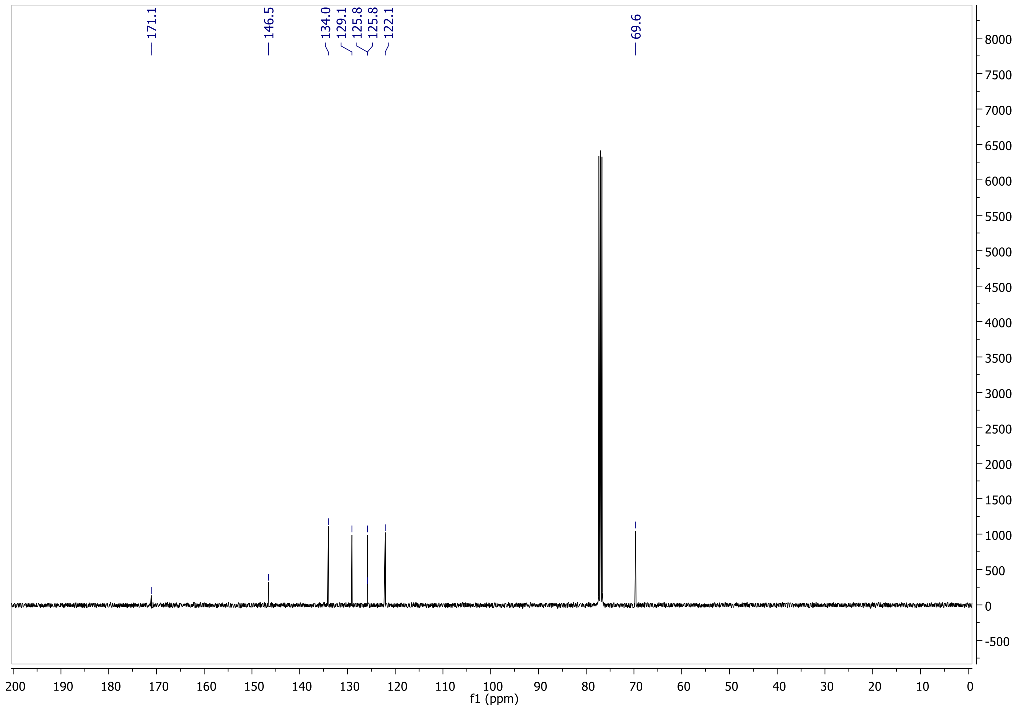
171.1
146.5
134.0
129.1
125.8
125.8
122.1
69.6
f1 (ppm)

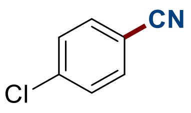

23

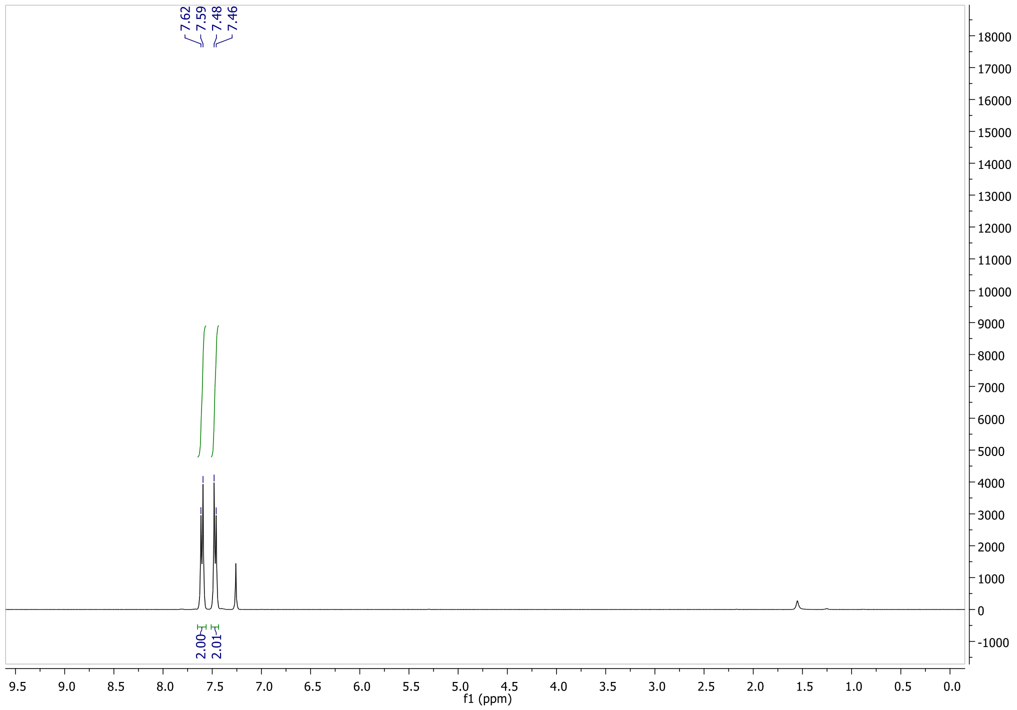

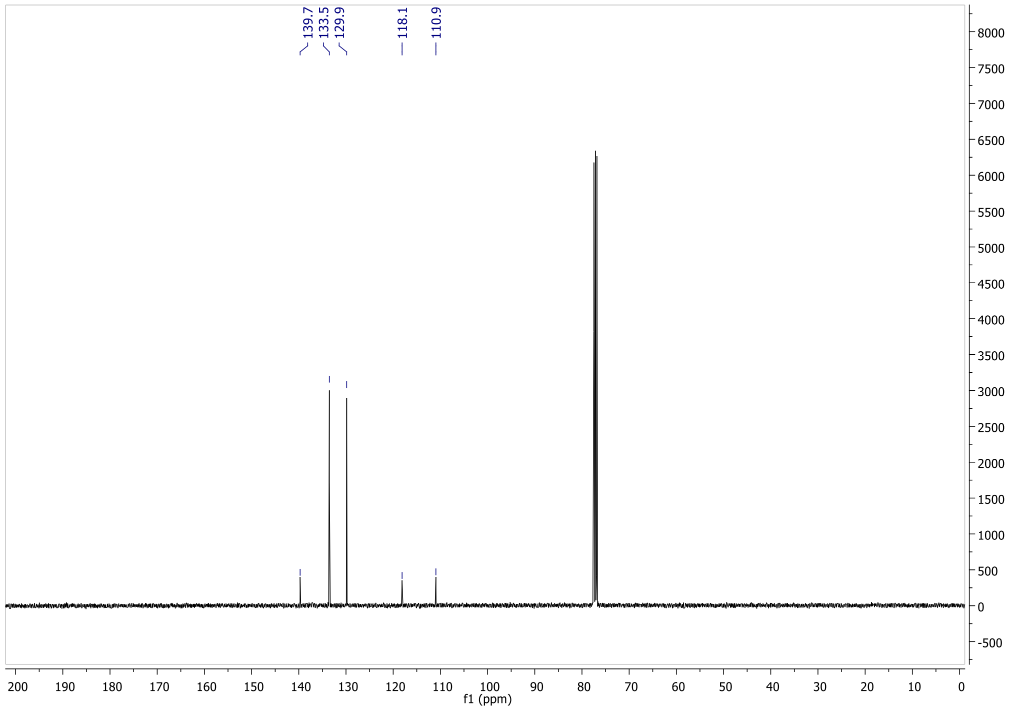

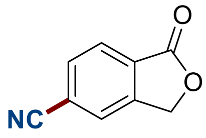

O
O
NC

24

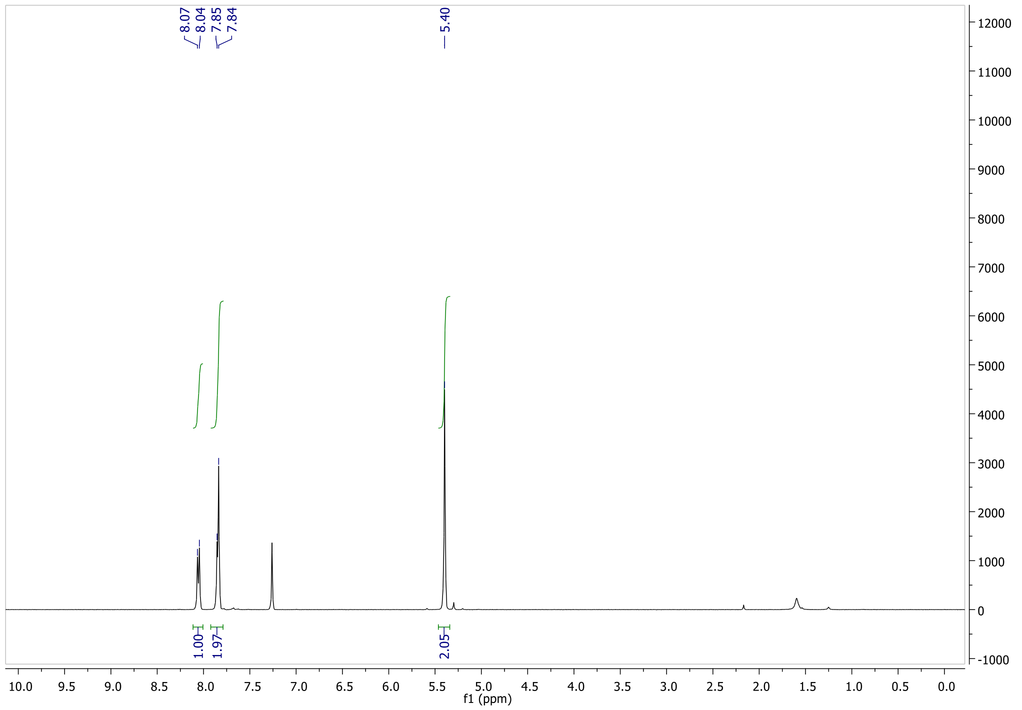

8.07
8.04
7.85
7.84
5.40
1.00
1.97
2.05
f1 (ppm)

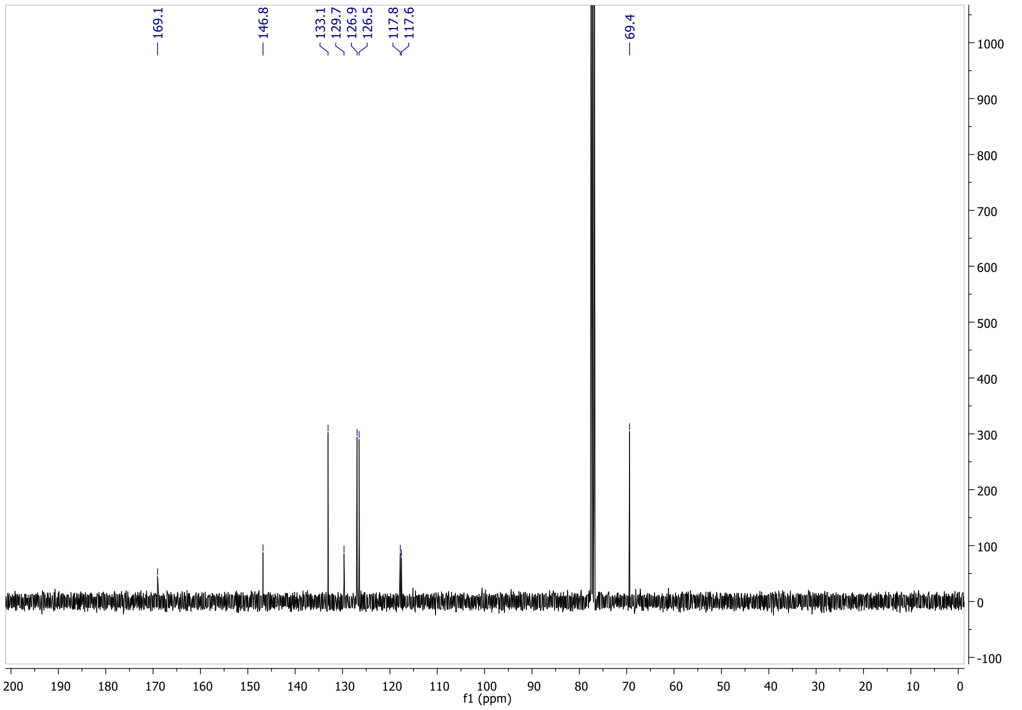

169.1
146.8
133.1
129.7
126.9
126.5
117.8
117.6
69.4
f1 (ppm)

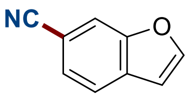

25

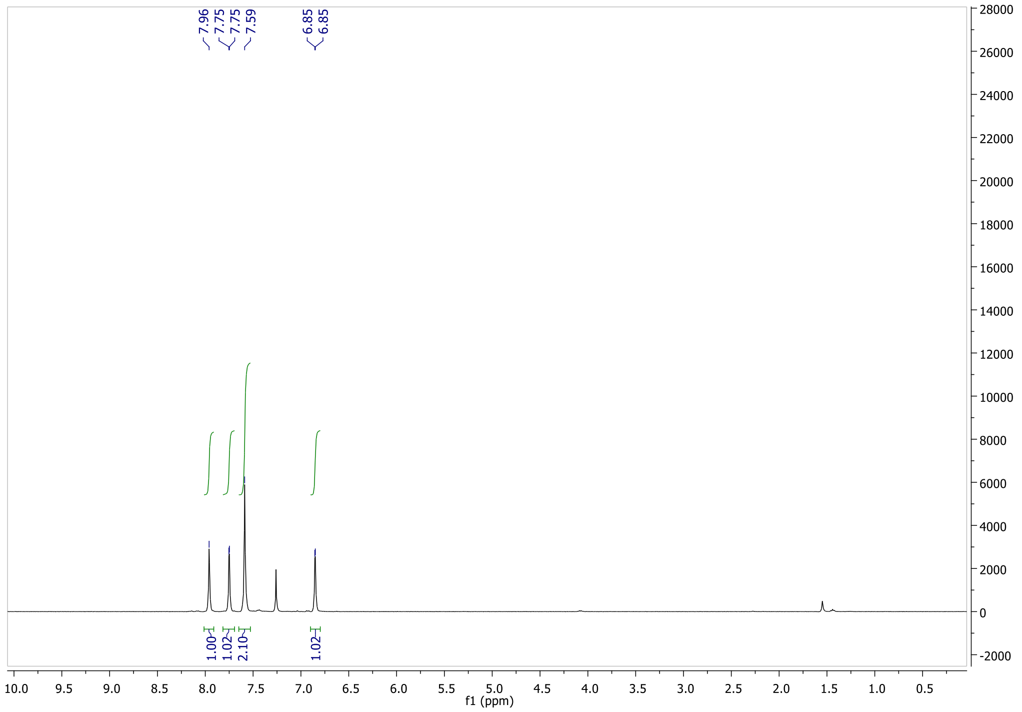

7.96
7.75
7.75
7.59
6.85
6.85
1.00
1.02
2.10
1.02
f1 (ppm)

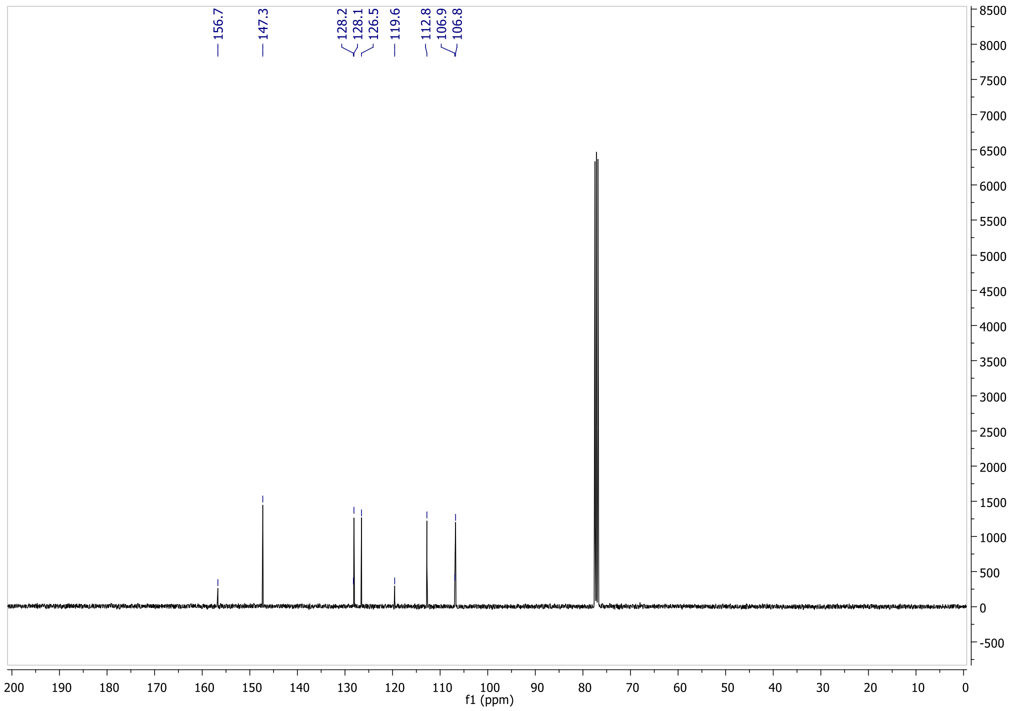

156.7
147.3
128.2
128.1
126.5
119.6
112.8
106.9
106.8
f1 (ppm)

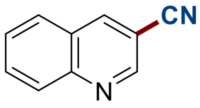

26

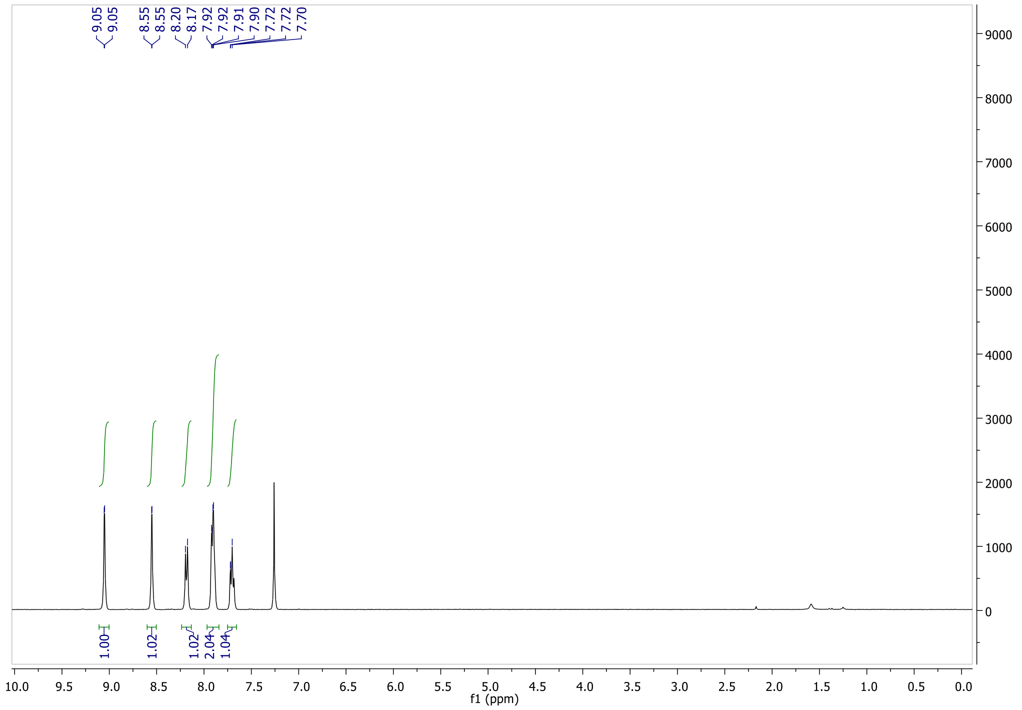

9.05
9.05
8.55
8.55
8.20
8.17
7.92
7.92
7.91
7.90
7.72
7.72
7.70
1.00
1.02
1.02
2.04
1.04
f1 (ppm)

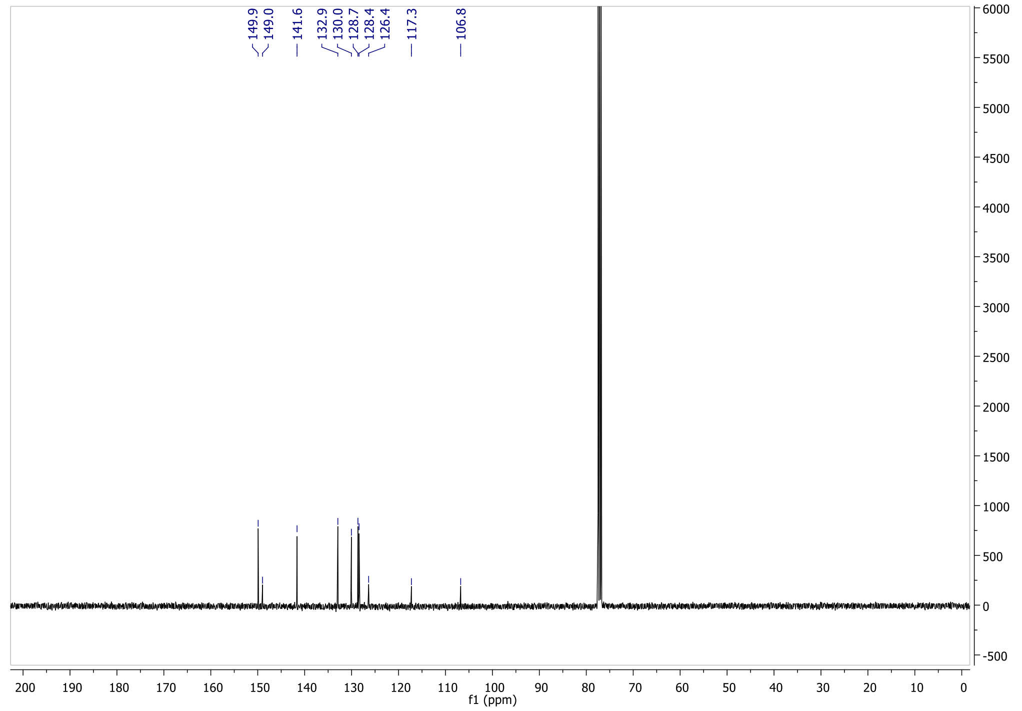

149.9
149.0
141.6
132.9
130.0
128.7
128.4
126.4
117.3
106.8
f1 (ppm)

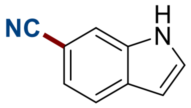
NC
H
N

27

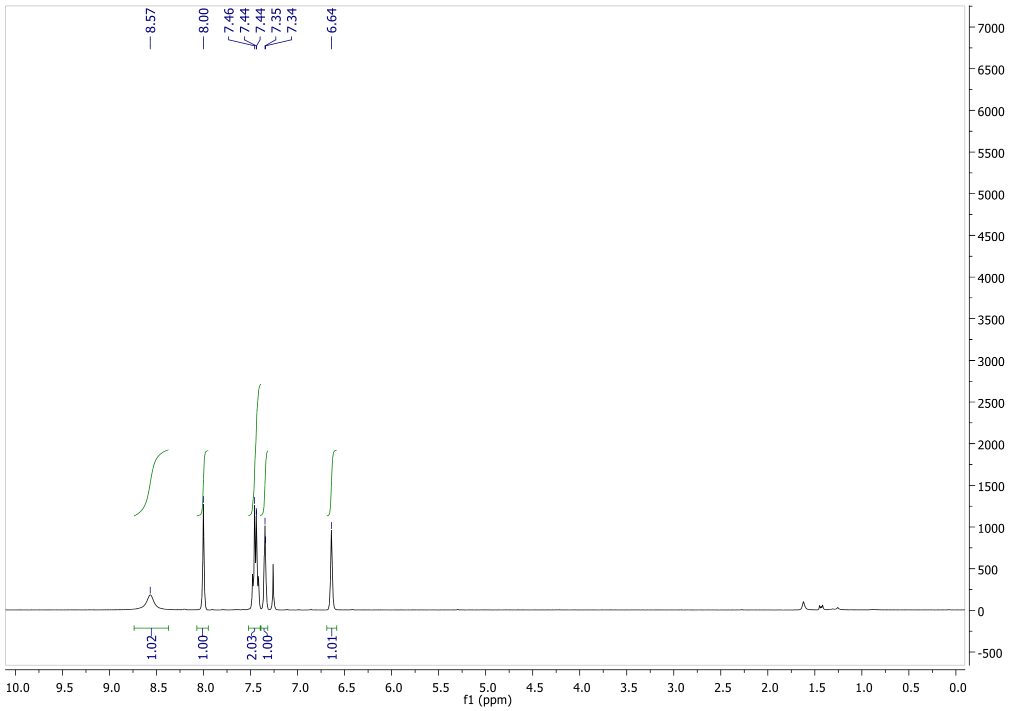
8.57
8.00
7.46
7.44
7.44
7.35
7.34
6.64
1.02
1.00
2.03
1.00
1.01
f1 (ppm)

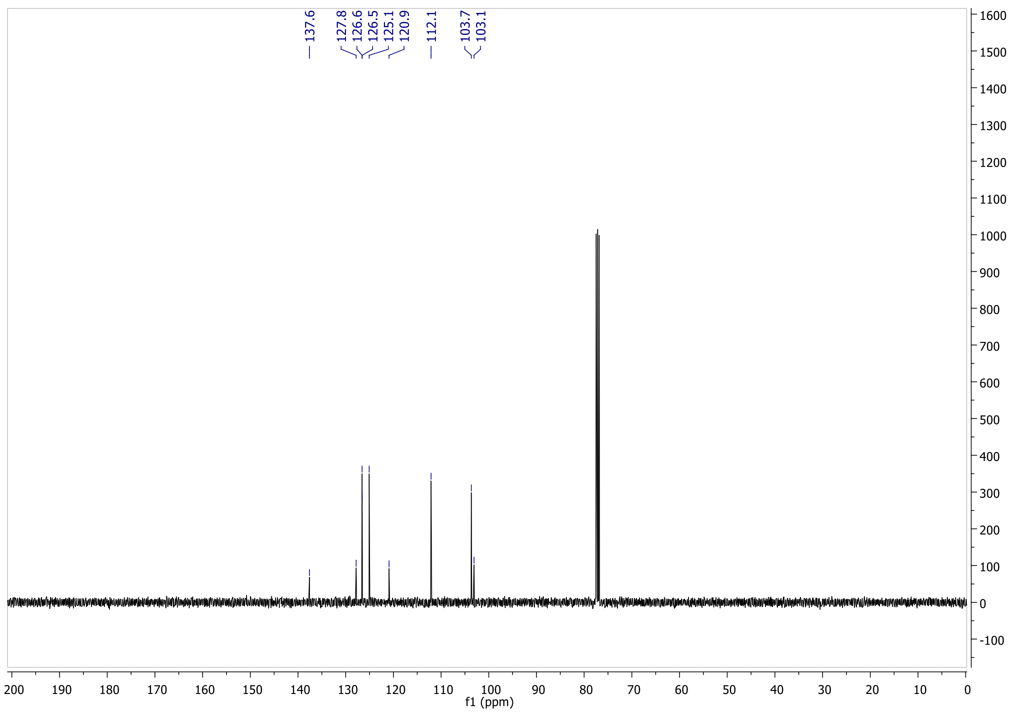
137.6
127.8
126.6
126.5
125.1
120.9
112.1
103.7
103.1
f1 (ppm)

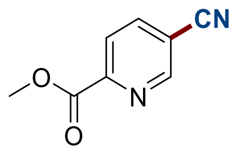
CN
O
N
O

28

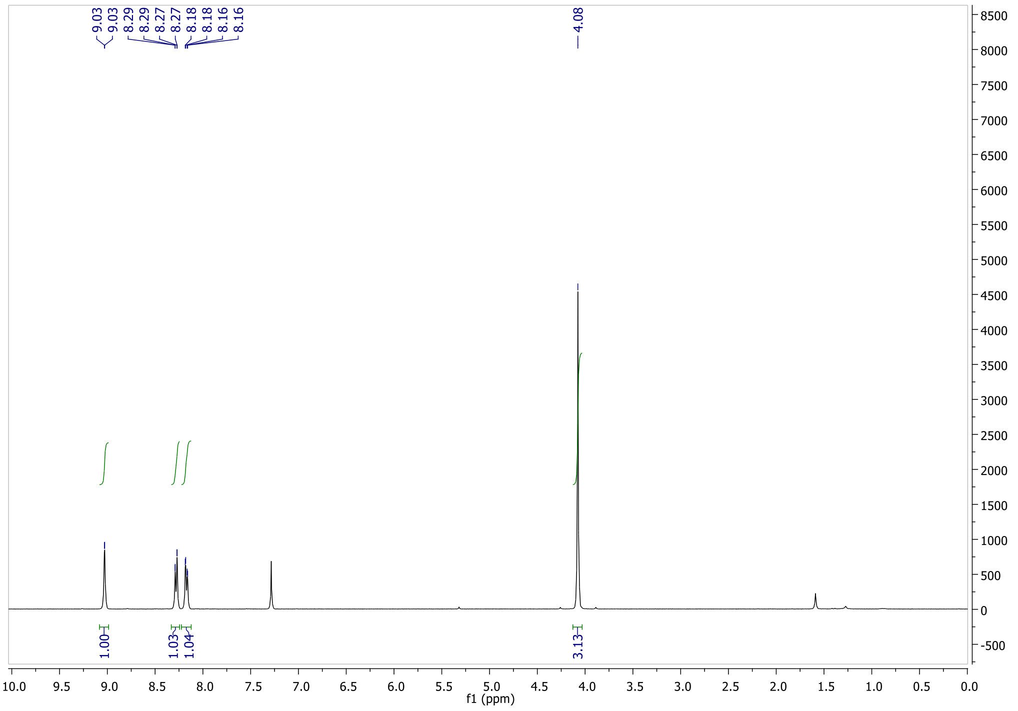
9.03
9.03
8.29
8.29
8.27
8.27
8.18
8.18
8.16
8.16
4.08
1.00
1.03
1.04
3.13
f1 (ppm)

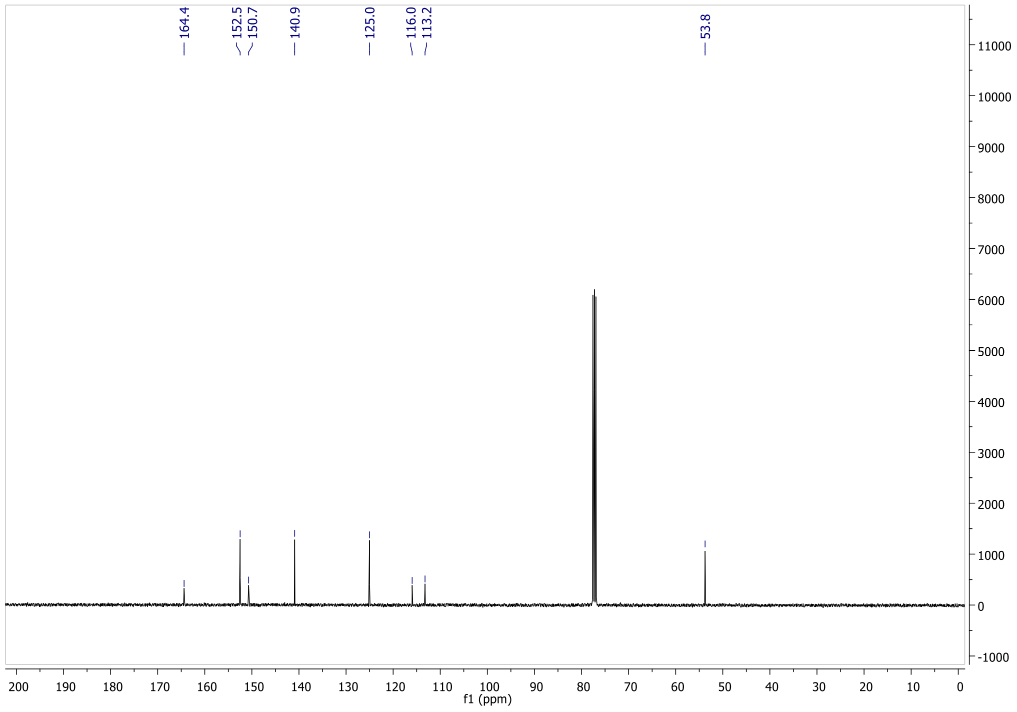
164.4
152.5
150.7
140.9
125.0
116.0
113.2
53.8
f1 (ppm)

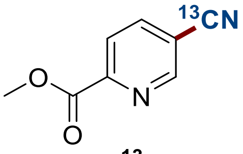
13CN
O
N
O

28-^{13}C

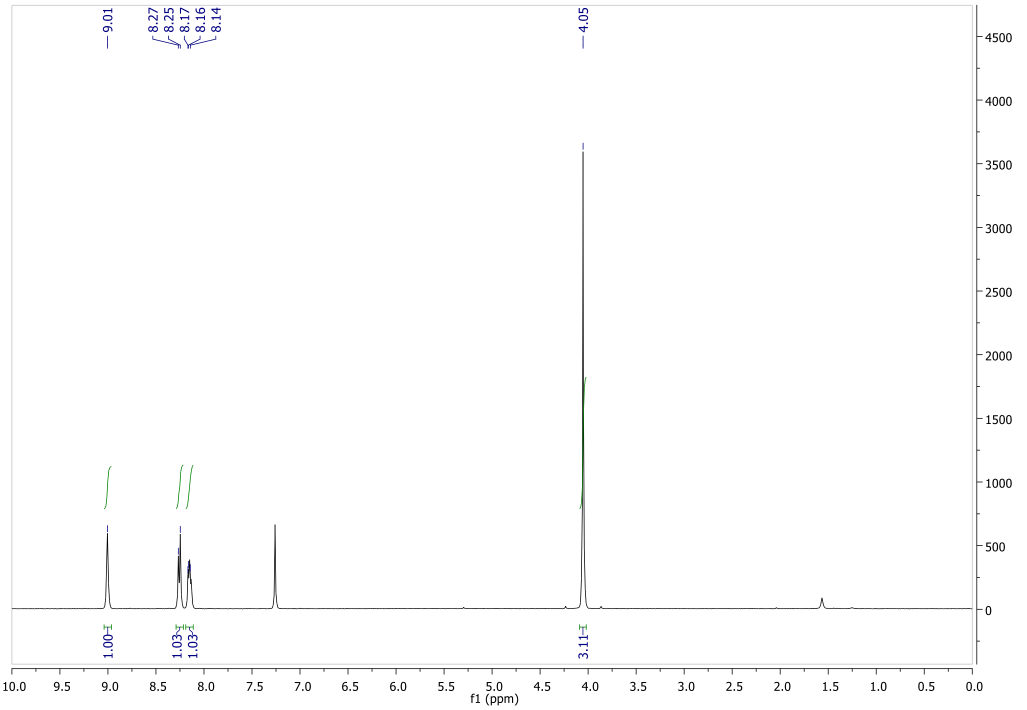
9.01
8.27
8.25
8.17
8.16
8.14
4.05
1.00
1.03
1.03
3.11
f1 (ppm)

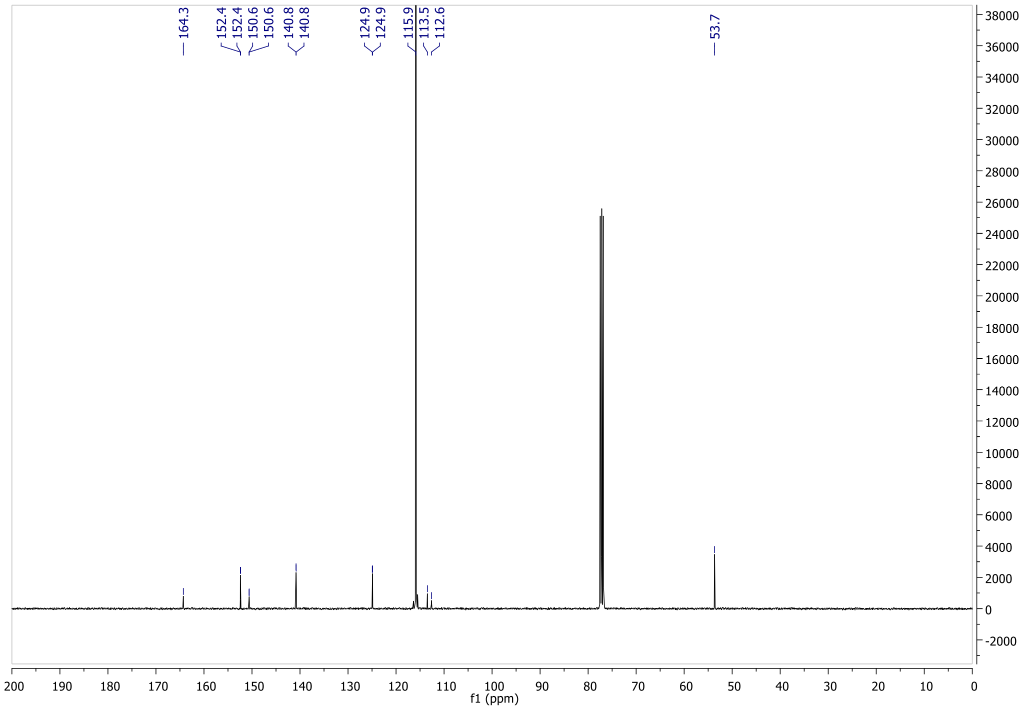
164.3
152.4
152.4
150.6
150.6
140.8
140.8
124.9
124.9
115.9
113.5
112.6
53.7
f1 (ppm)

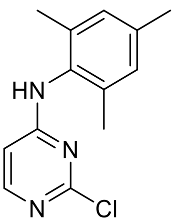
HN
N
N
Cl

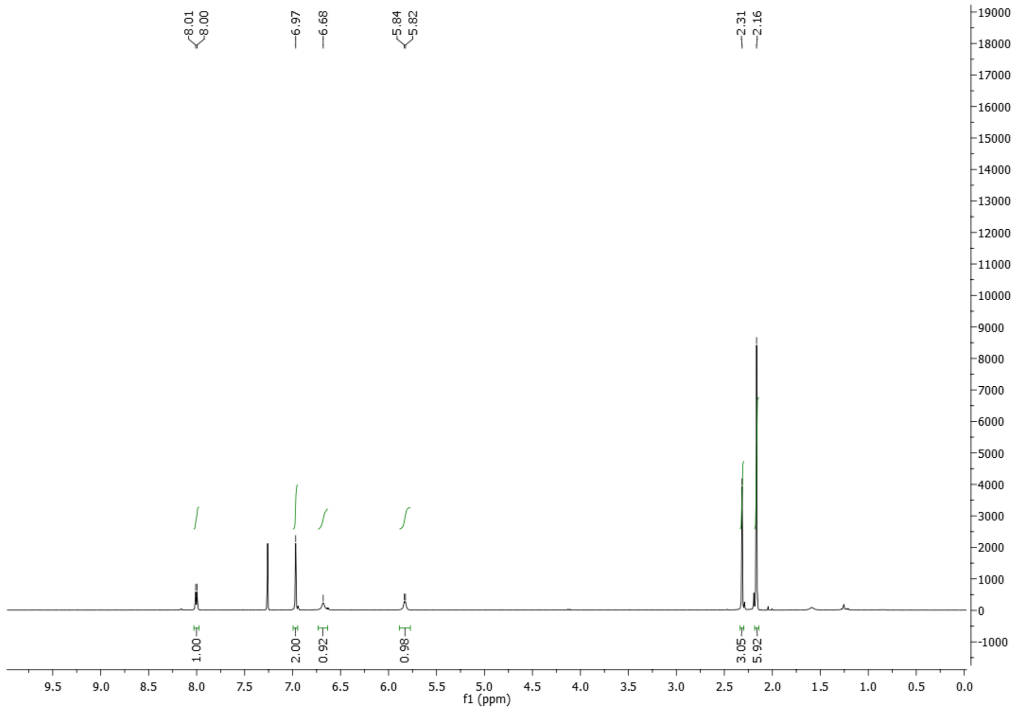
8.01
8.00
6.97
6.68
5.84
5.82
2.31
2.16
1.00
2.00
0.92
0.98
3.05
5.92
f1 (ppm)

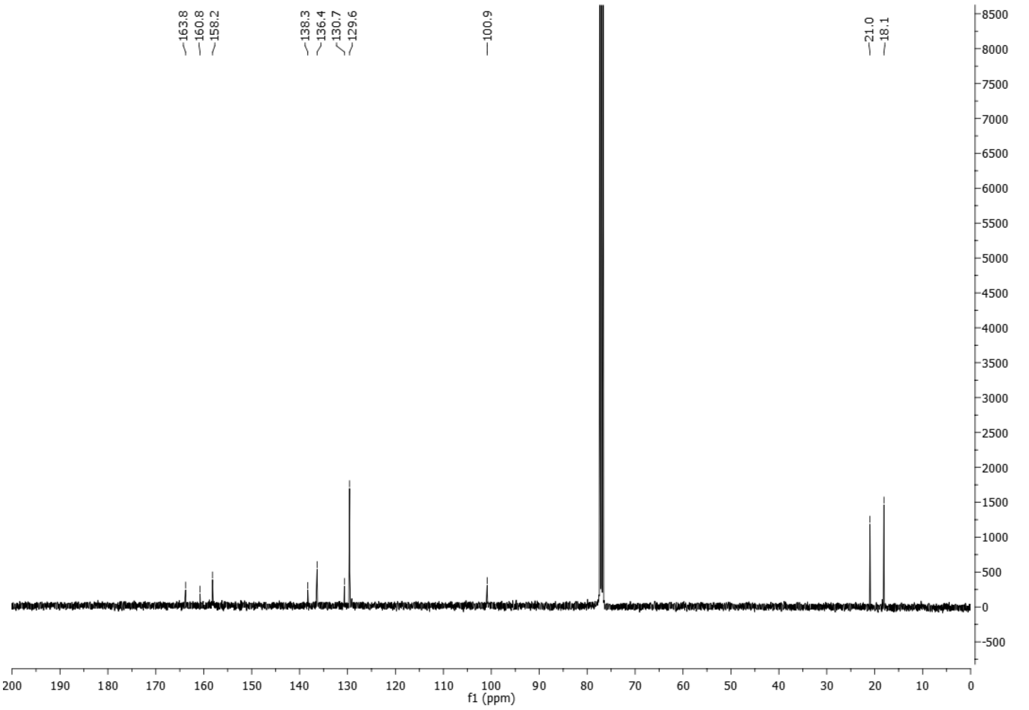
163.8
160.8
158.2
138.3
136.4
130.7
129.6
100.9
21.0
18.1
f1 (ppm)

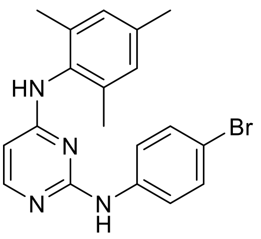
HN
N
N
N
H
Br

29a

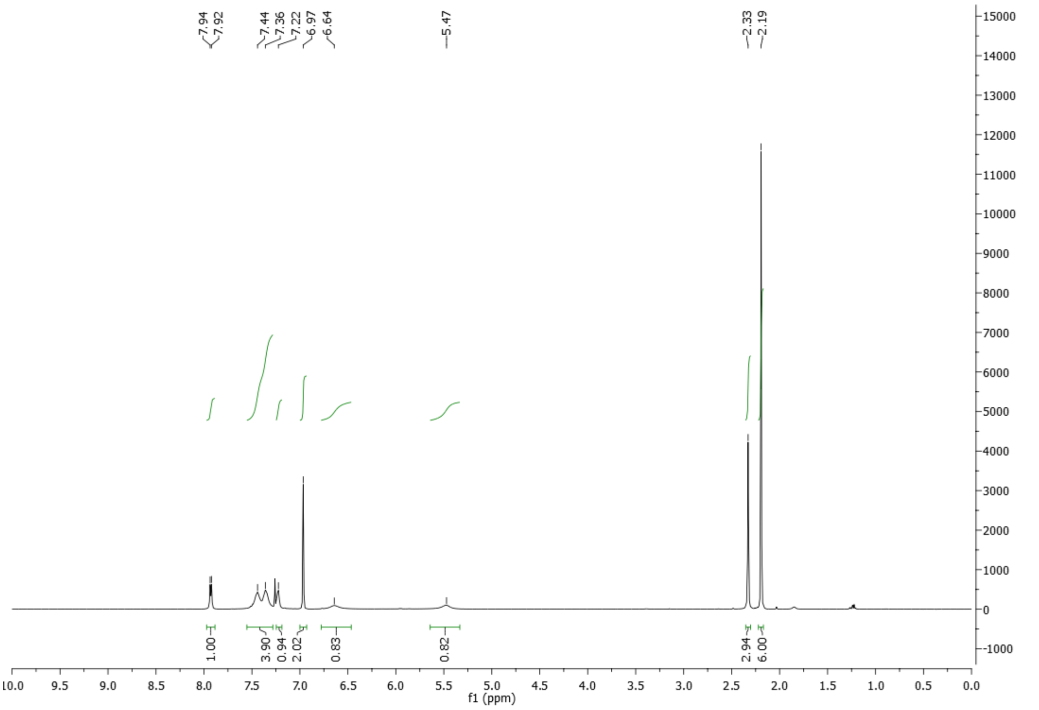
7.94
7.92
7.44
7.36
7.22
6.97
6.64
5.47
2.33
2.19
1.00
3.90
0.94
2.02
0.83
0.82
2.94
6.00
f1 (ppm)

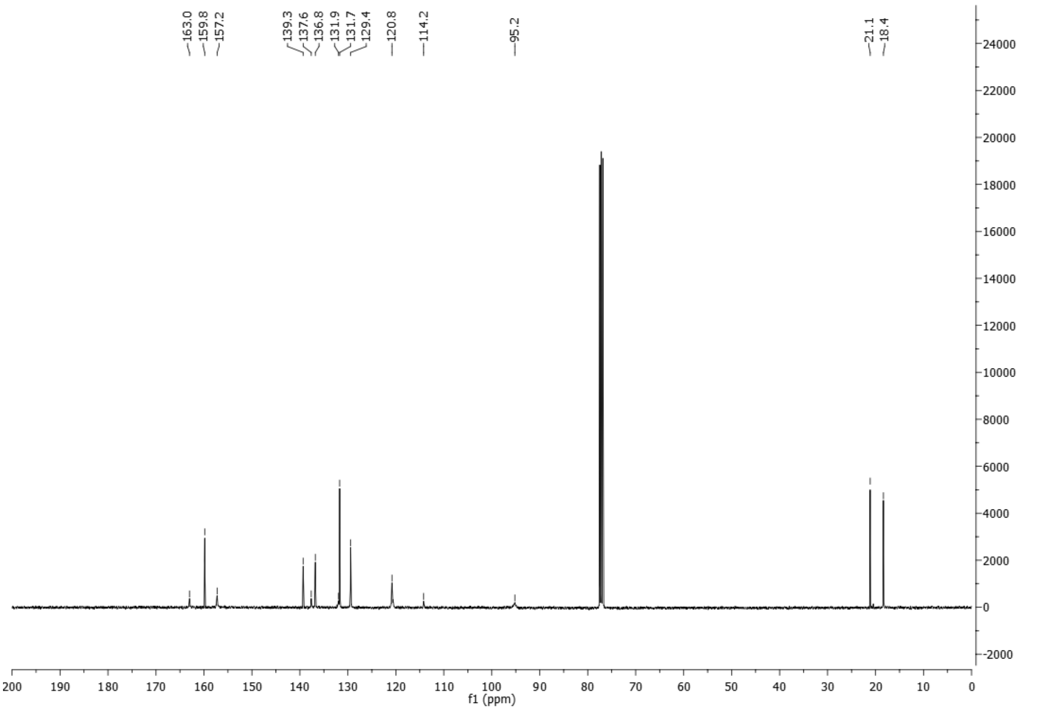
163.0
159.8
157.2
139.3
137.6
136.8
131.9
131.7
129.4
120.8
114.2
95.2
21.1
18.4
f1 (ppm)

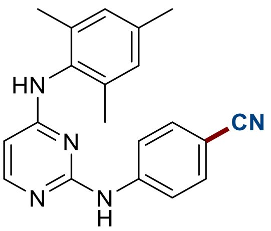
HN
N
N
N
H
CN

29

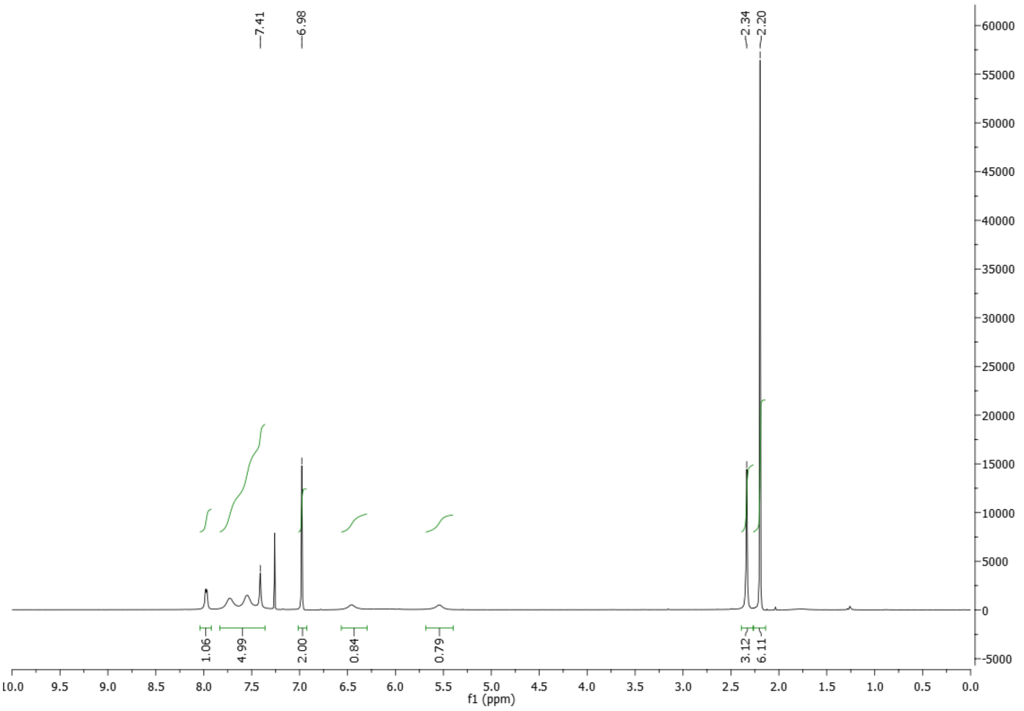
7.41
6.98
2.34
2.20
1.06
4.99
2.00
0.84
0.79
3.12
6.11
f1 (ppm)

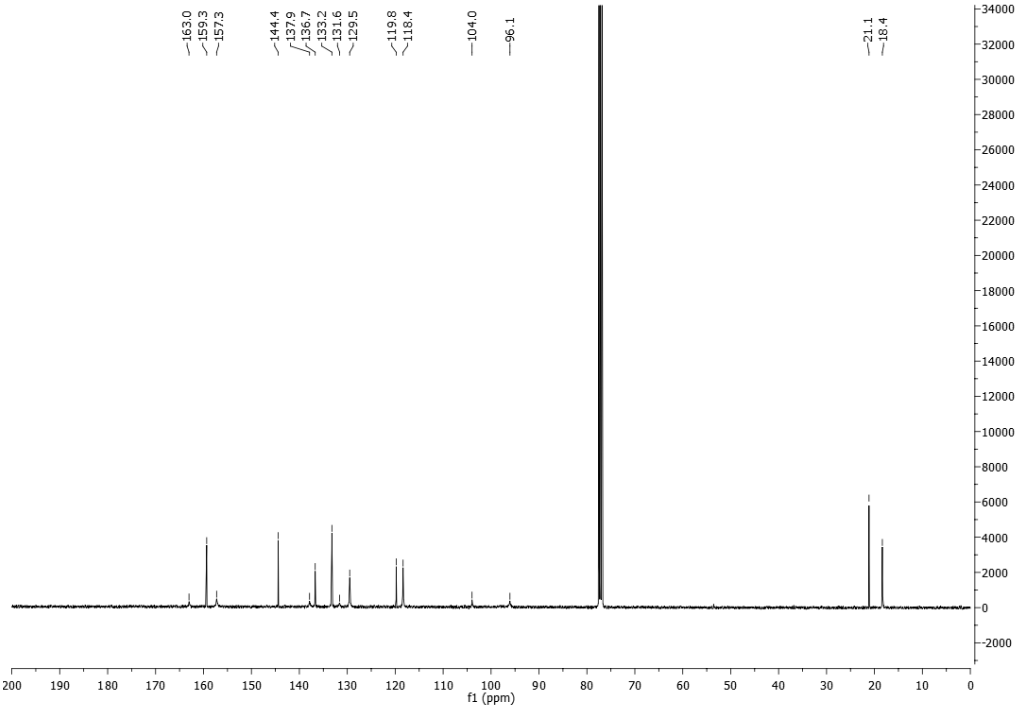
163.0
159.3
157.3
144.4
137.9
136.7
133.2
131.6
129.5
119.8
118.4
104.0
96.1
21.1
18.4
f1 (ppm)

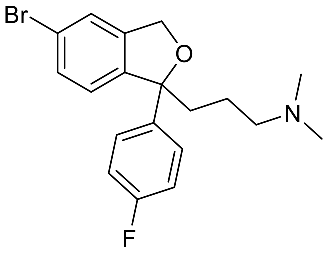
Br
O
N
F

30a

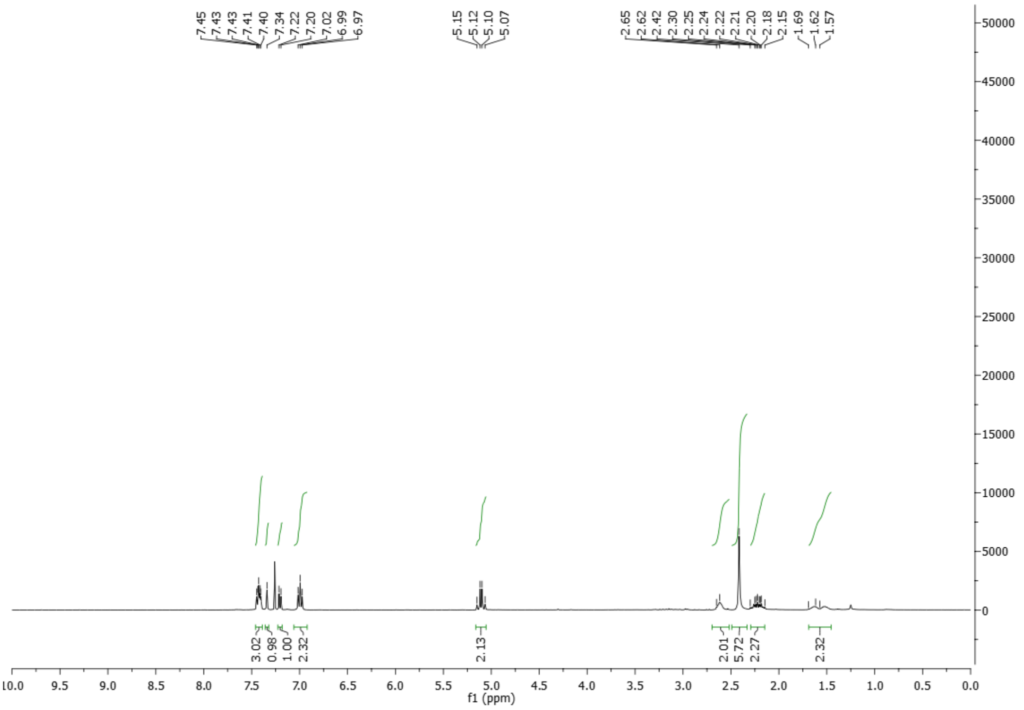
7.45
7.43
7.43
7.41
7.40
7.34
7.22
7.20
7.02
6.99
6.97
5.15
5.12
5.10
5.07
2.65
2.62
2.42
2.30
2.25
2.24
2.22
2.21
2.20
2.18
2.15
1.69
1.62
1.57
3.02
0.98
1.00
2.32
2.13
2.01
5.72
2.27
2.32
f1 (ppm)

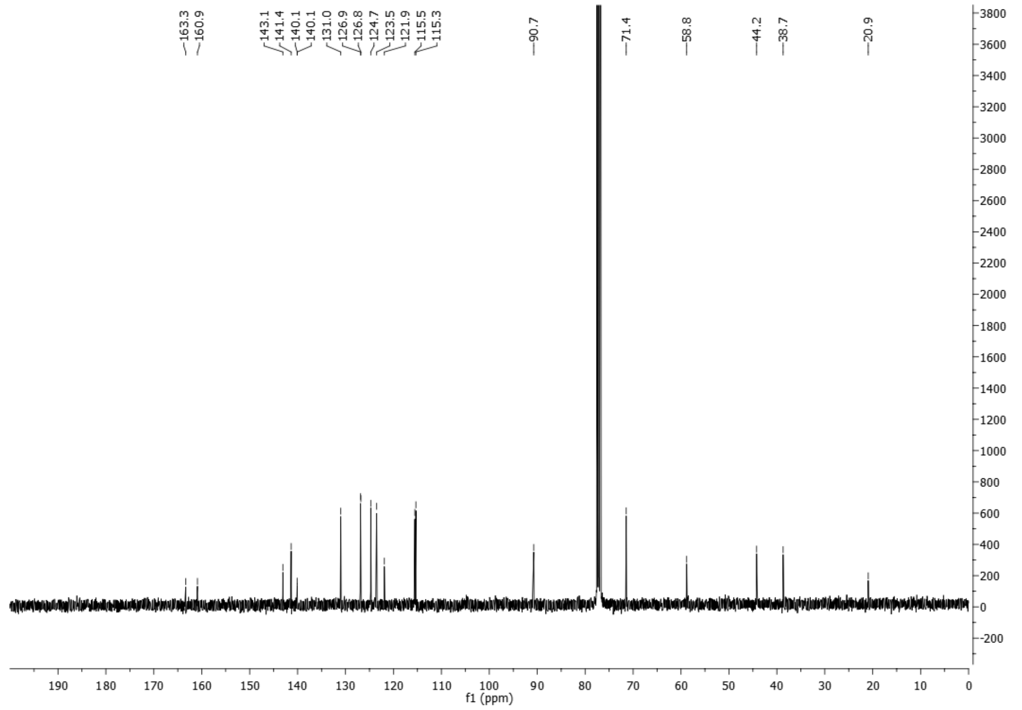
163.3
160.9
143.1
141.4
140.1
140.1
131.0
126.9
126.8
124.7
123.5
121.9
115.5
115.3
90.7
71.4
58.8
44.2
38.7
20.9
f1 (ppm)

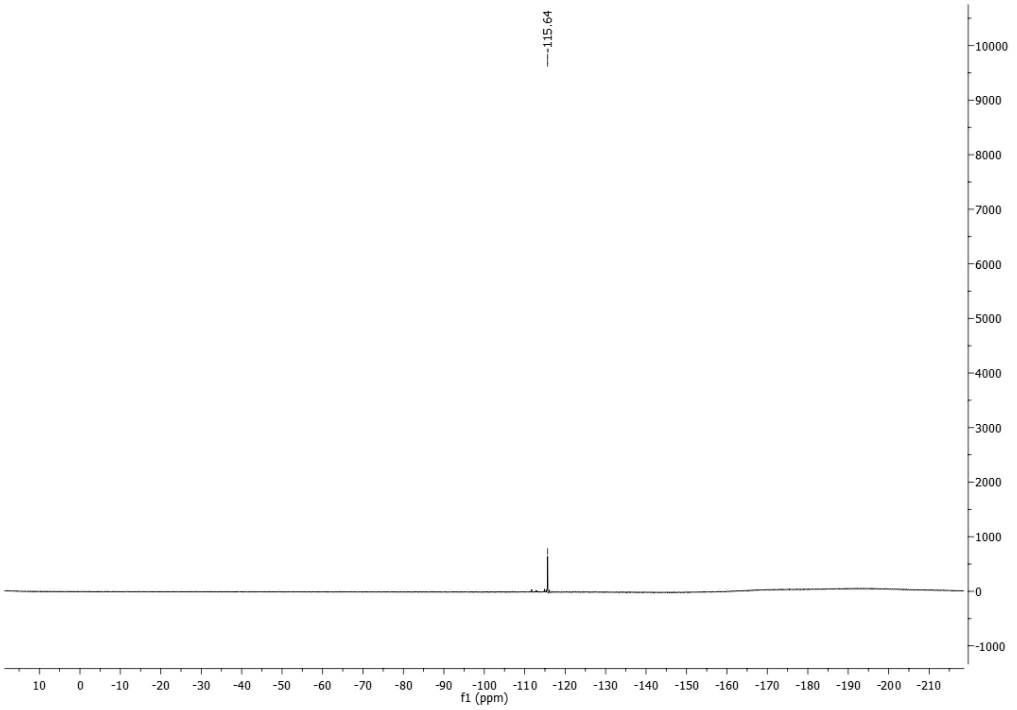

-115.64
10000
9000
8000
7000
6000
5000
4000
3000
2000
1000
0
-1000
10 0 -10 -20 -30 -40 -50 -60 -70 -80 -90 -100 -110 -120 -130 -140 -150 -160 -170 -180 -190 -200 -210
f1 (ppm)

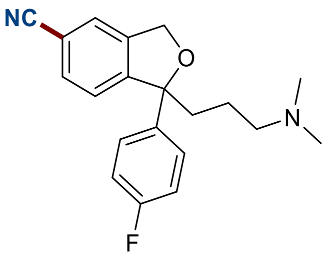
NC
O
N
F

30

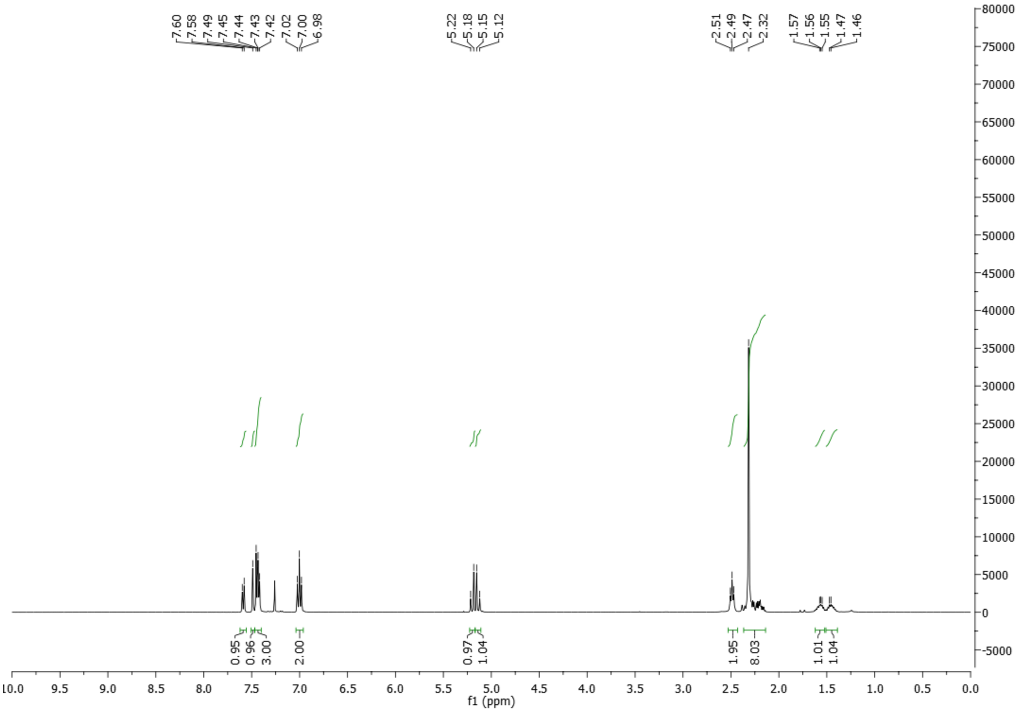
7.60
7.58
7.49
7.45
7.44
7.43
7.42
7.02
7.00
6.98
5.22
5.18
5.15
5.12
2.51
2.49
2.47
2.32
1.57
1.56
1.55
1.47
1.46
0.95
0.96
3.00
2.00
0.97
1.04
1.95
8.03
1.01
1.04
f1 (ppm)

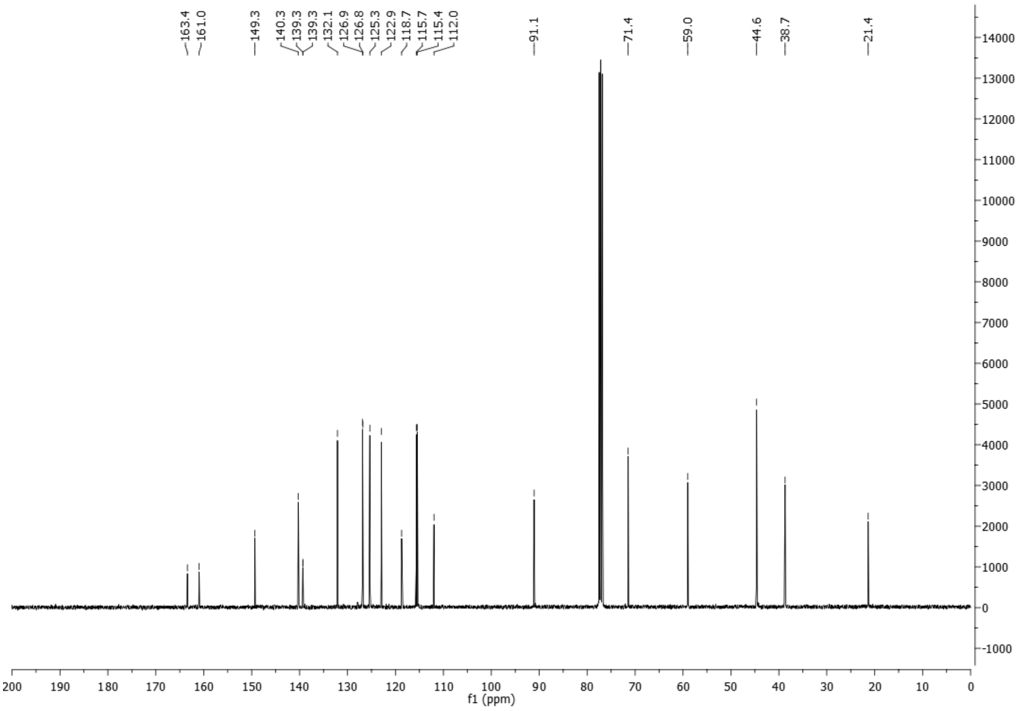
163.4
161.0
149.3
140.3
139.3
139.3
132.1
126.9
126.8
125.3
122.9
118.7
115.7
115.4
112.0
91.1
71.4
59.0
44.6
38.7
21.4
f1 (ppm)

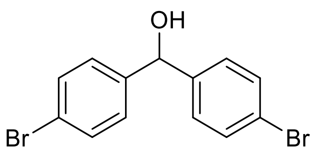
OH
Br
Br

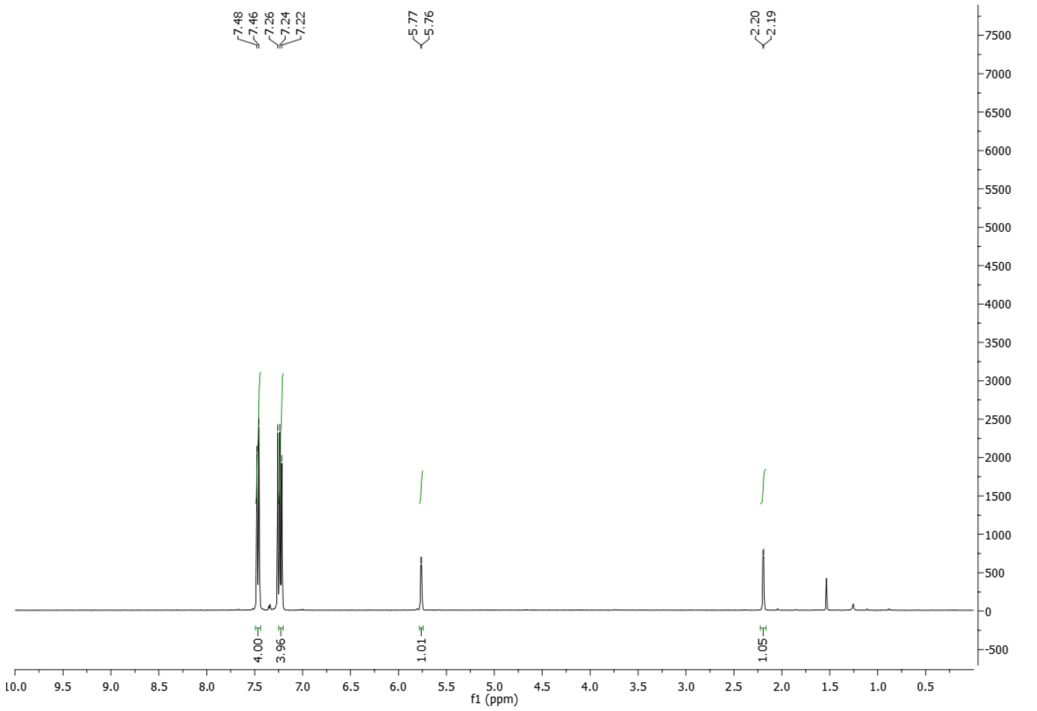
7.48
7.46
7.26
7.24
7.22
5.77
5.76
2.20
2.19
4.00
3.96
1.01
1.05
f1 (ppm)

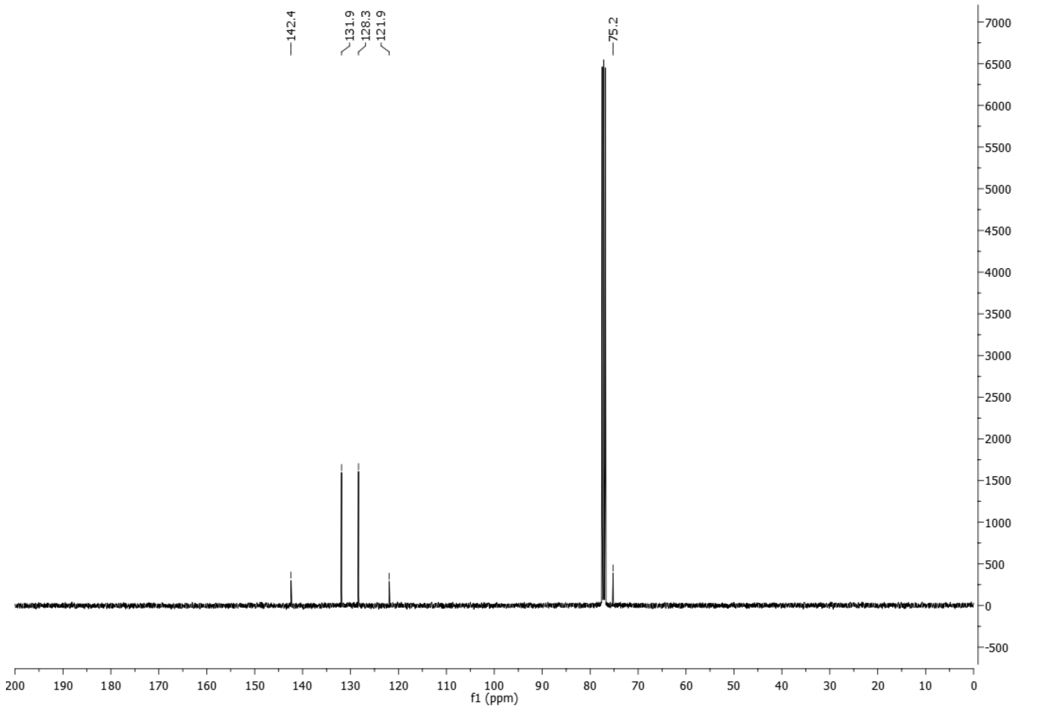
142.4
131.9
128.3
121.9
75.2
f1 (ppm)

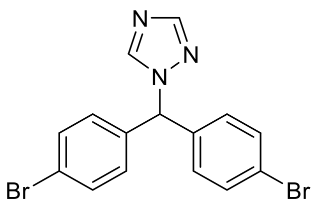
N
N
N
Br
Br

31a

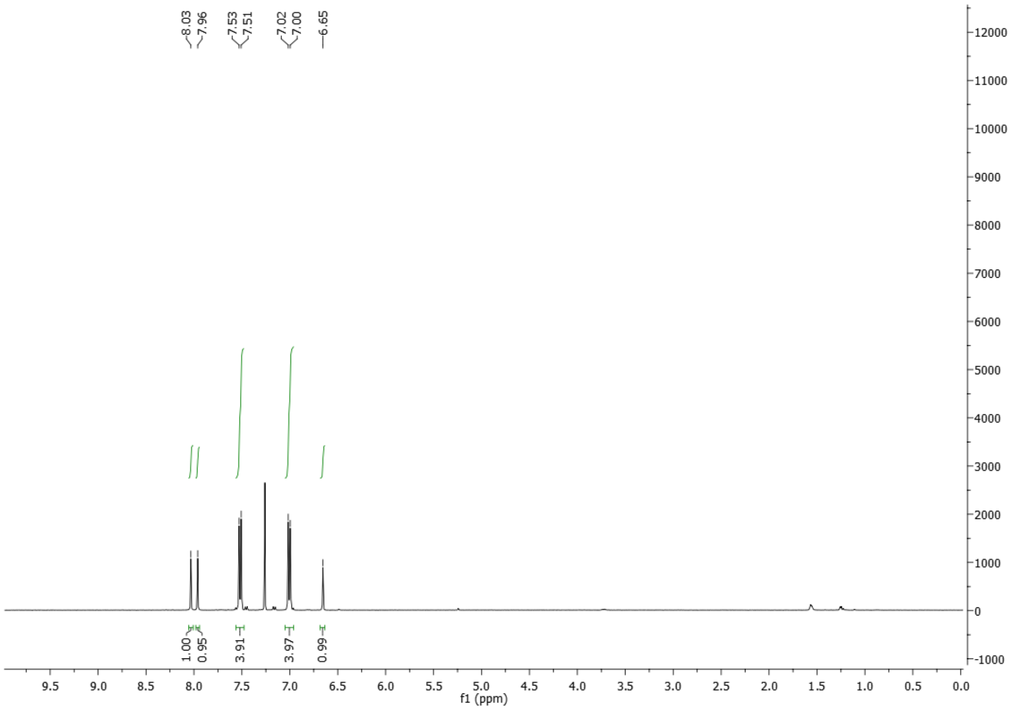
8.03
7.96
7.53
7.51
7.02
7.00
6.65
1.00
0.95
3.91
3.97
0.99
f1 (ppm)

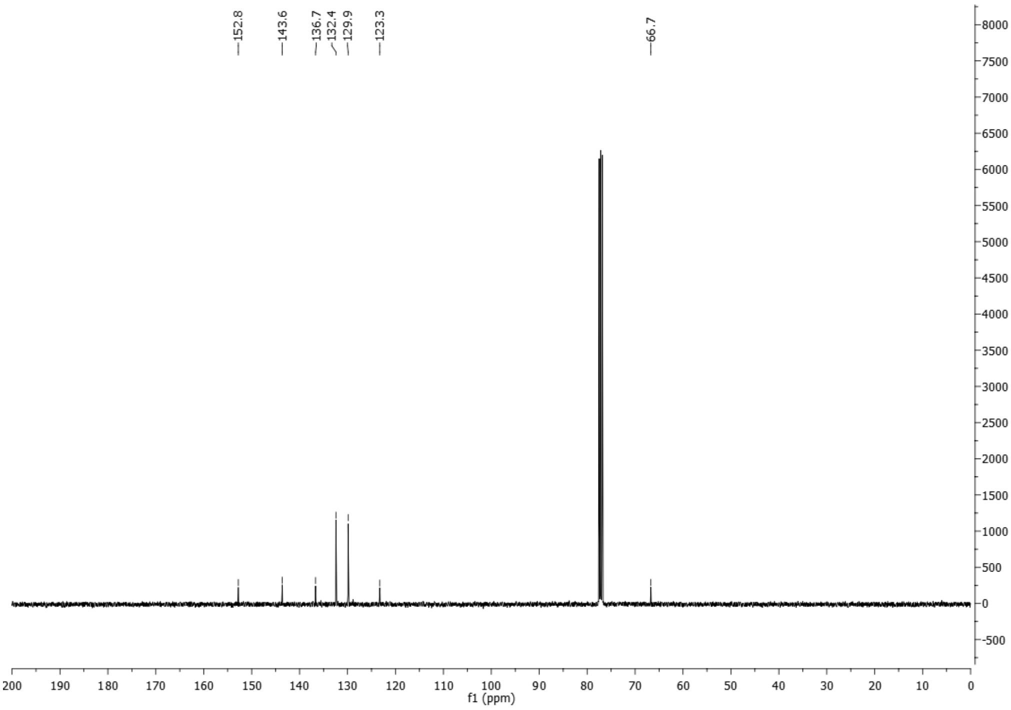
152.8
143.6
136.7
132.4
129.9
123.3
66.7
f1 (ppm)

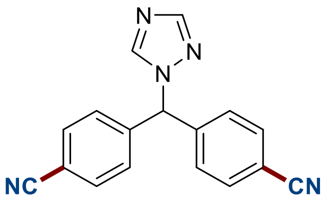
N
N
N
NC
CN

31

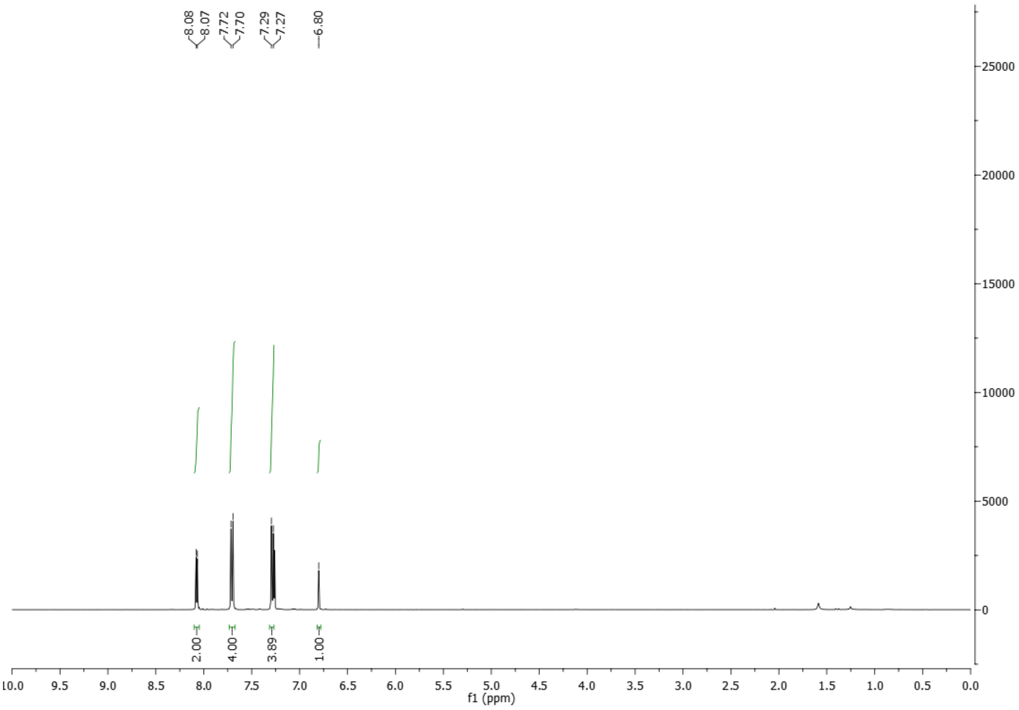
8.08
8.07
7.72
7.70
7.29
7.27
6.80
2.00
4.00
3.89
1.00
f1 (ppm)

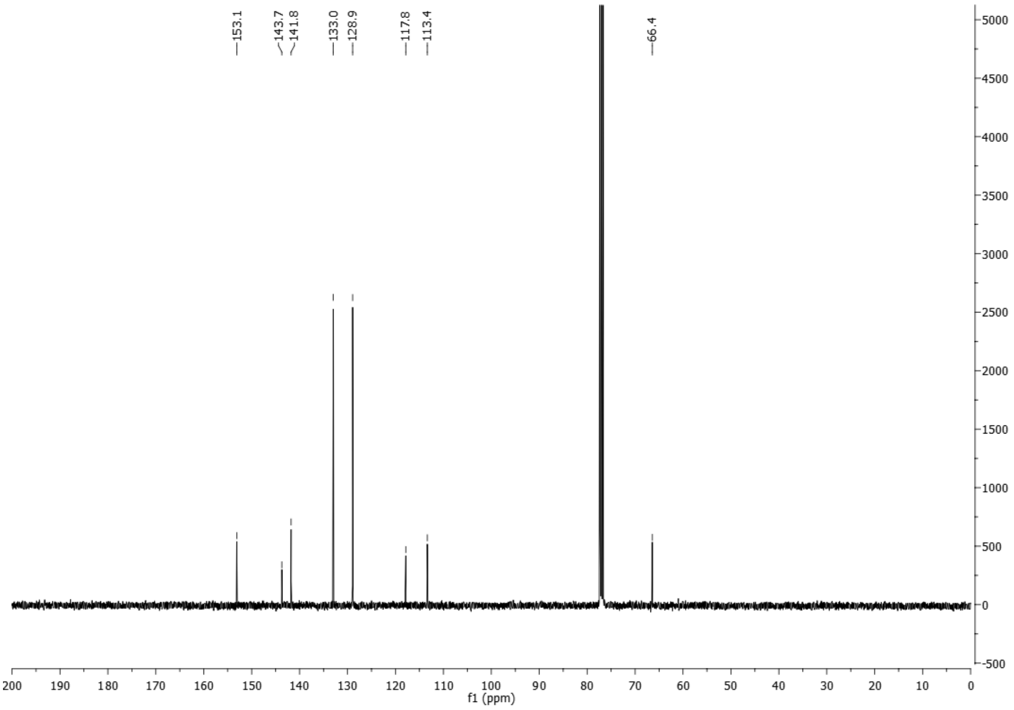
153.1
143.7
141.8
133.0
128.9
117.8
113.4
66.4
f1 (ppm)

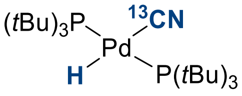
(tBu)3P
13CN
Pd
H
P(tBu)3

32

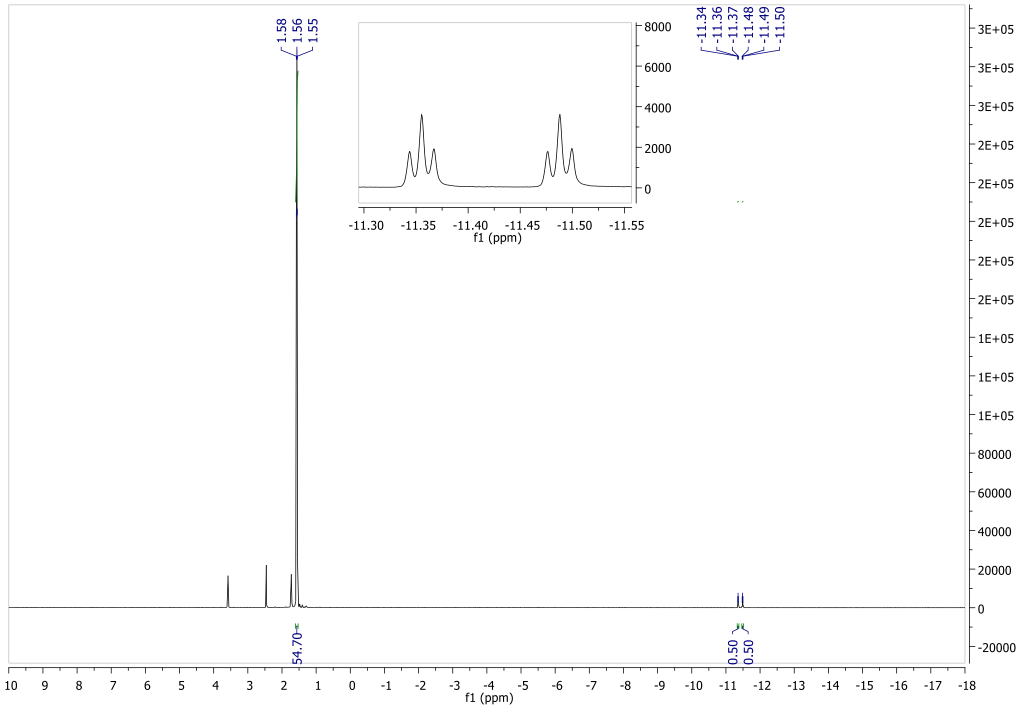
1.58
1.56
1.55
-11.34
-11.36
-11.37
-11.48
-11.49
-11.50
54.70
0.50
0.50
f1 (ppm)

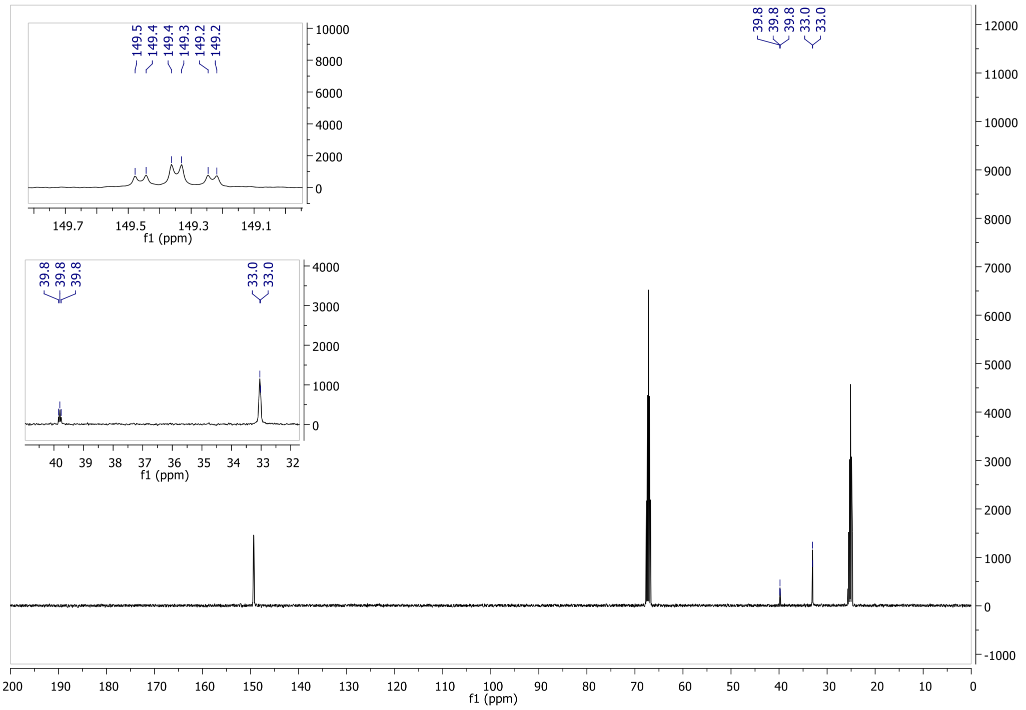
149.5
149.4
149.4
149.3
149.2
149.2
39.8
39.8
39.8
33.0
33.0
f1 (ppm)

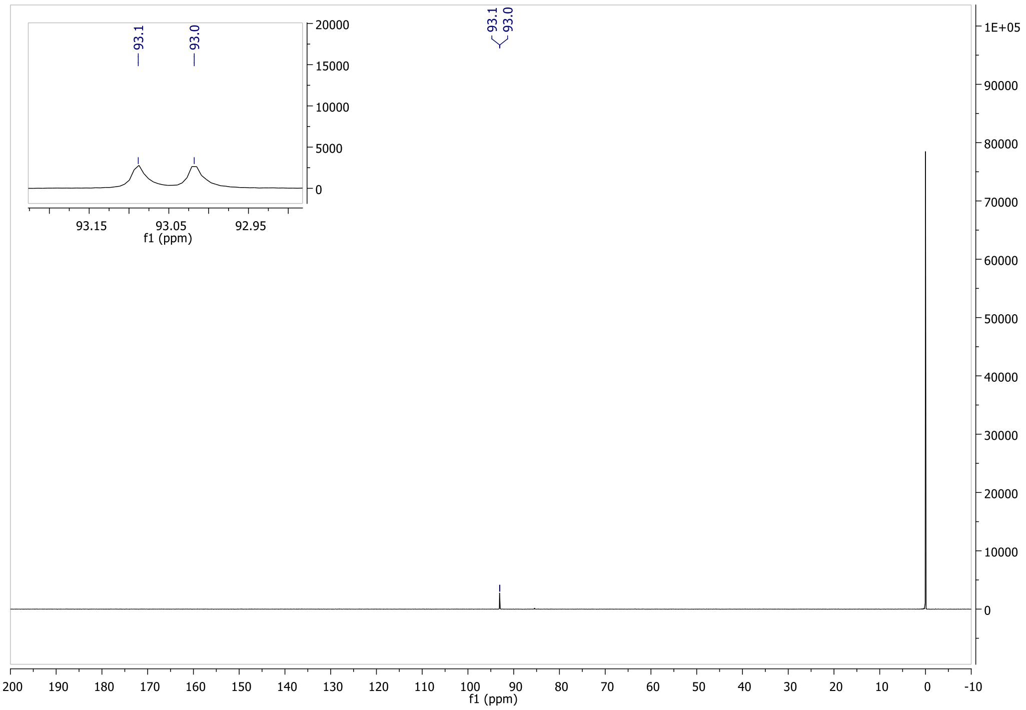

93.1
93.0
f1 (ppm)

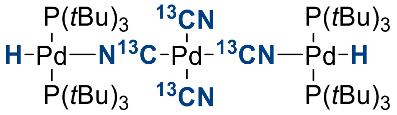

33

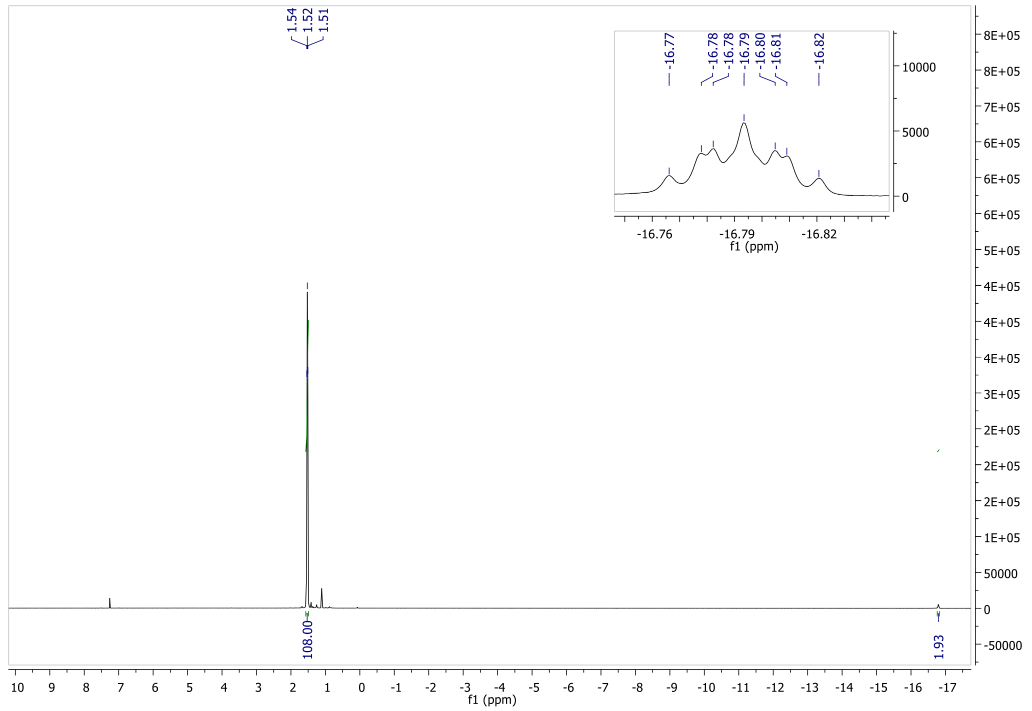

1.54
1.52
1.51
-16.77
-16.78
-16.78
-16.79
-16.80
-16.81
-16.82
108.00
1.93
f1 (ppm)

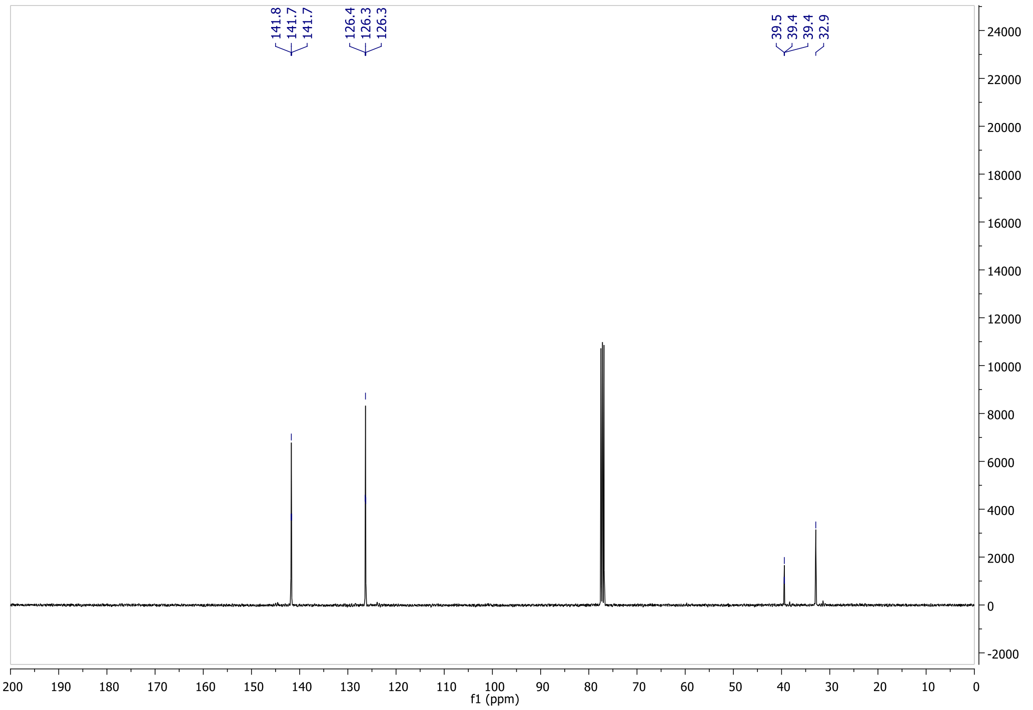

141.8
141.7
141.7
126.4
126.3
126.3
39.5
39.4
39.4
32.9
f1 (ppm)

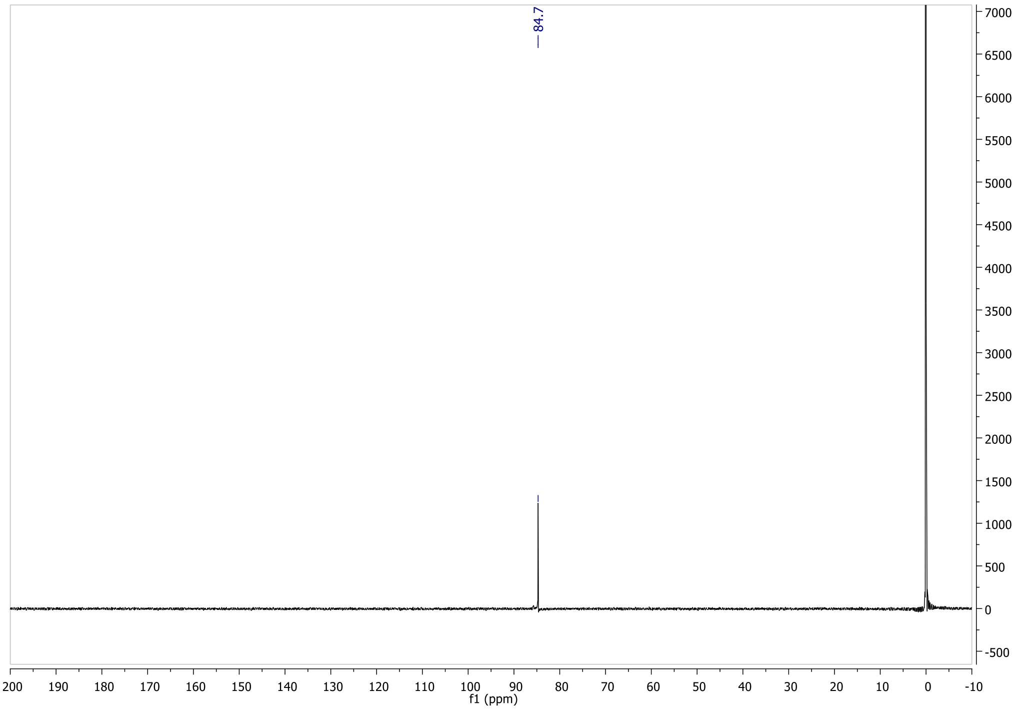
84.7
f1 (ppm)

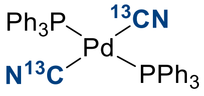
Ph3P
13CN
Pd
N13C
PPh3

34

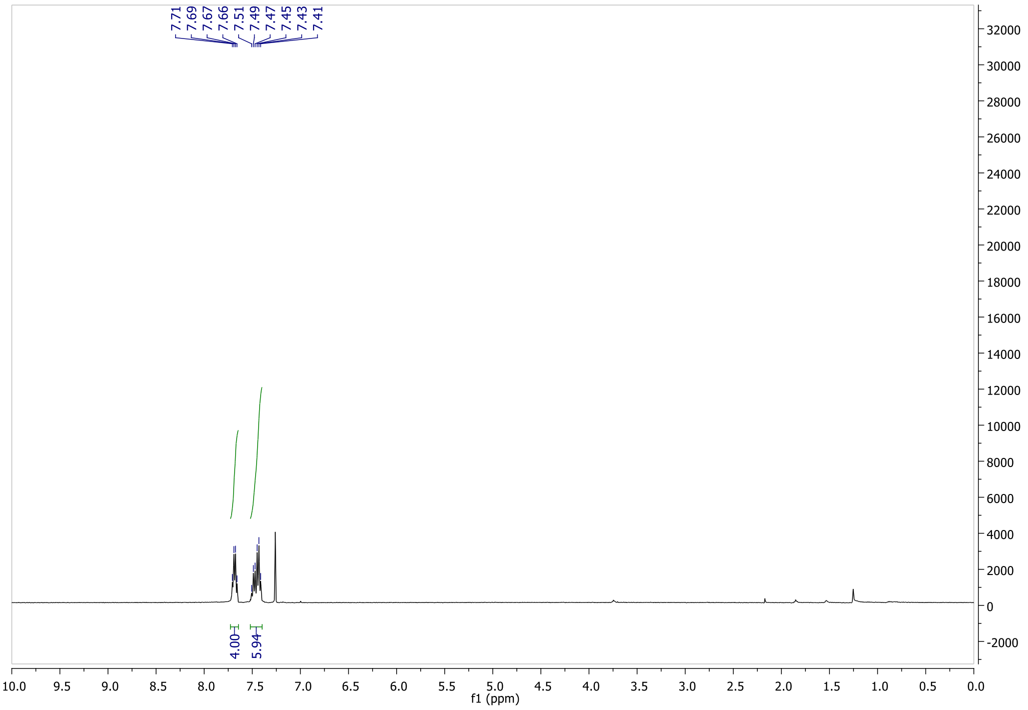
7.71
7.69
7.67
7.66
7.51
7.49
7.47
7.45
7.43
7.41
4.00
5.94
f1 (ppm)

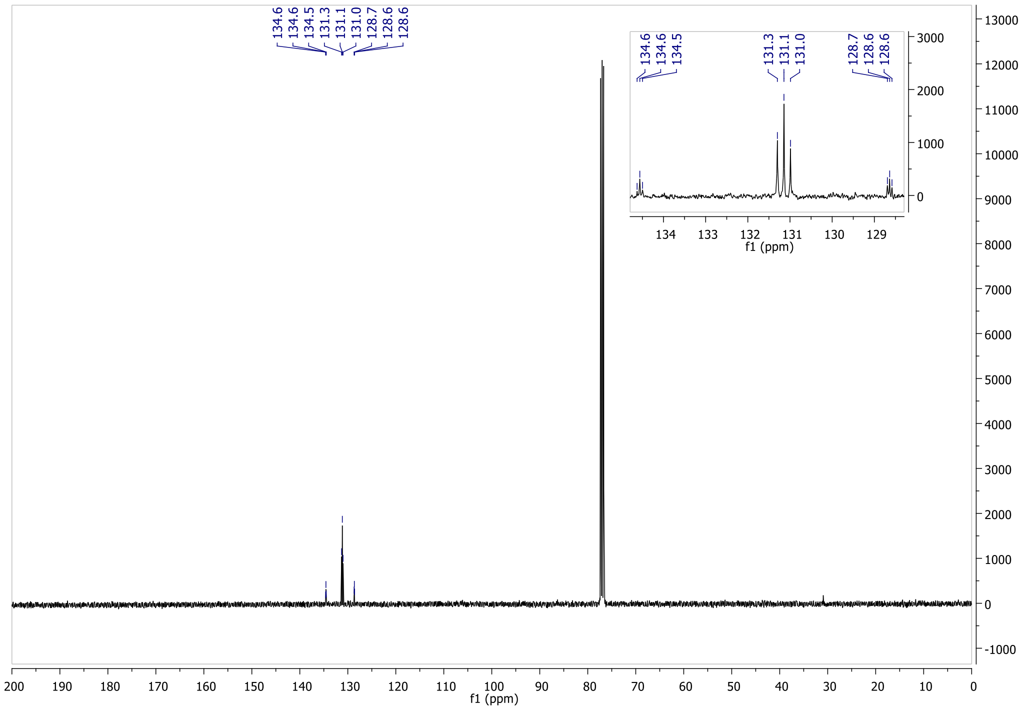
134.6
134.6
134.5
131.3
131.1
131.0
128.7
128.6
128.6
f1 (ppm)

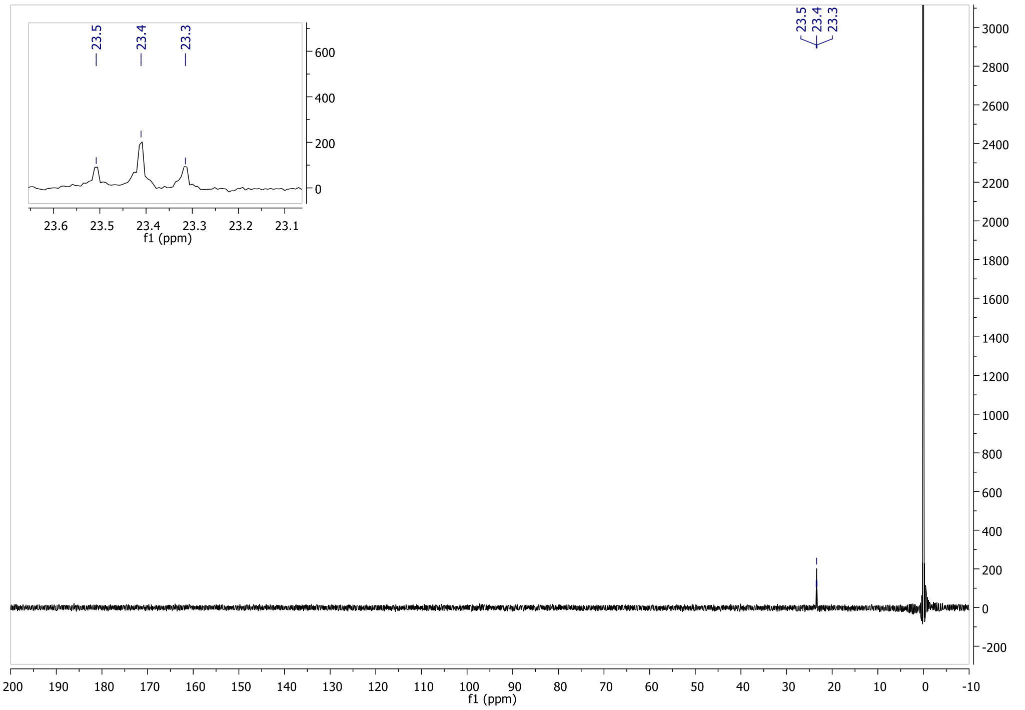

23.5
23.4
23.3
f1 (ppm)
f1 (ppm)

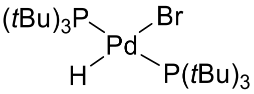
(tBu)3P
Br
Pd
H
P(tBu)3

35

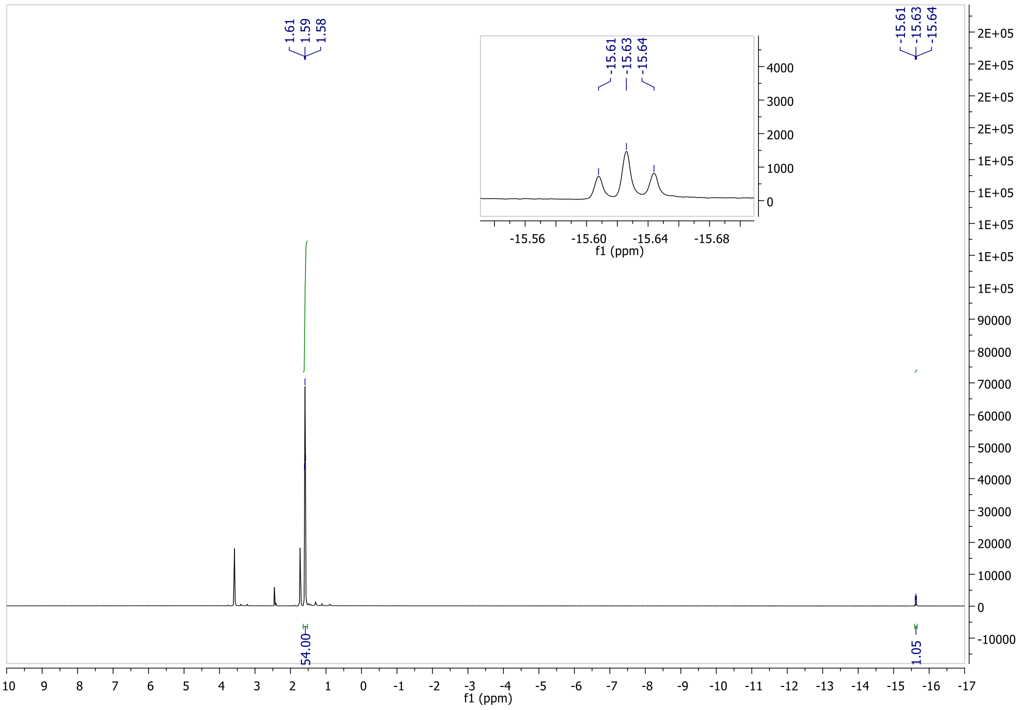
1.61
1.59
1.58
-15.61
-15.63
-15.64
54.00
1.05
f1 (ppm)

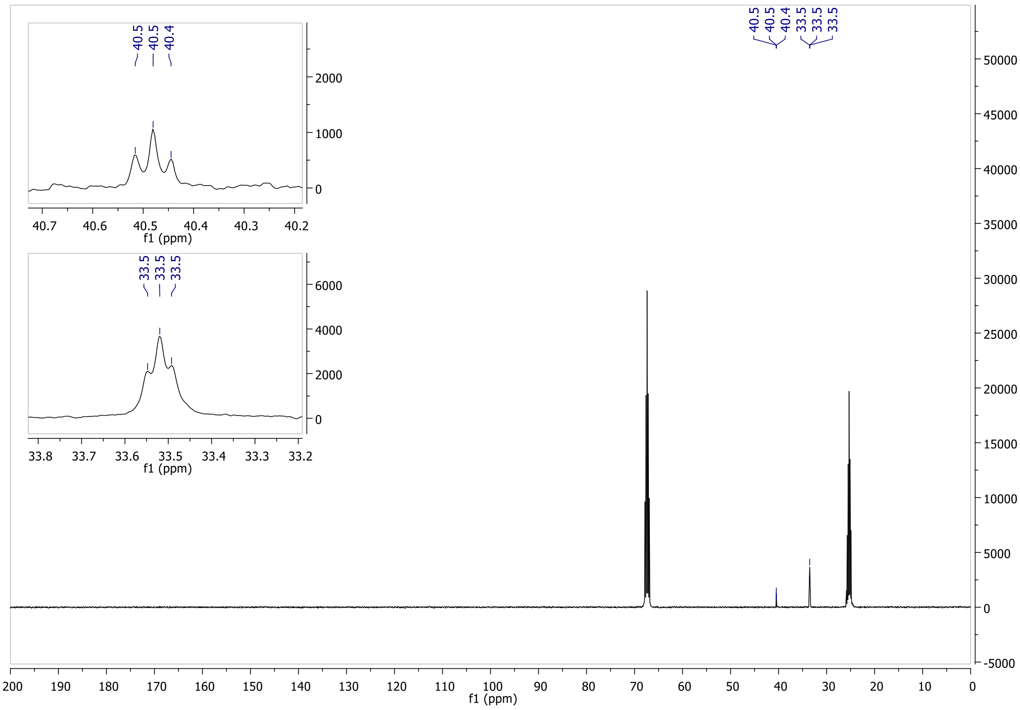
40.5
40.5
40.4
33.5
33.5
33.5
f1 (ppm)

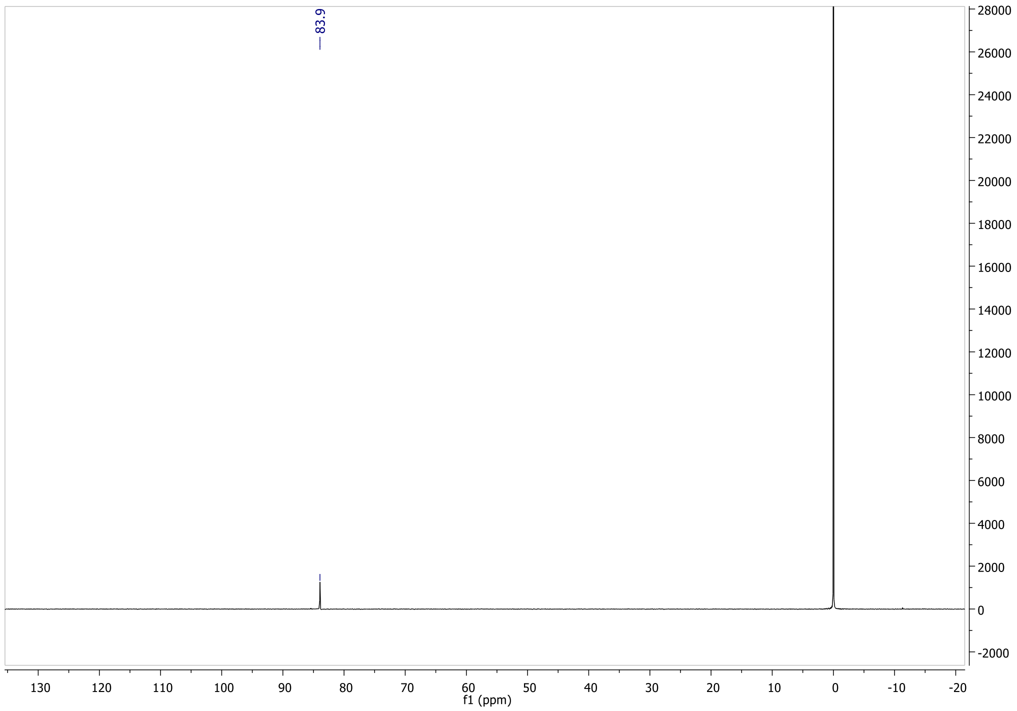
83.9
f1 (ppm)

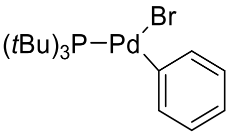

36

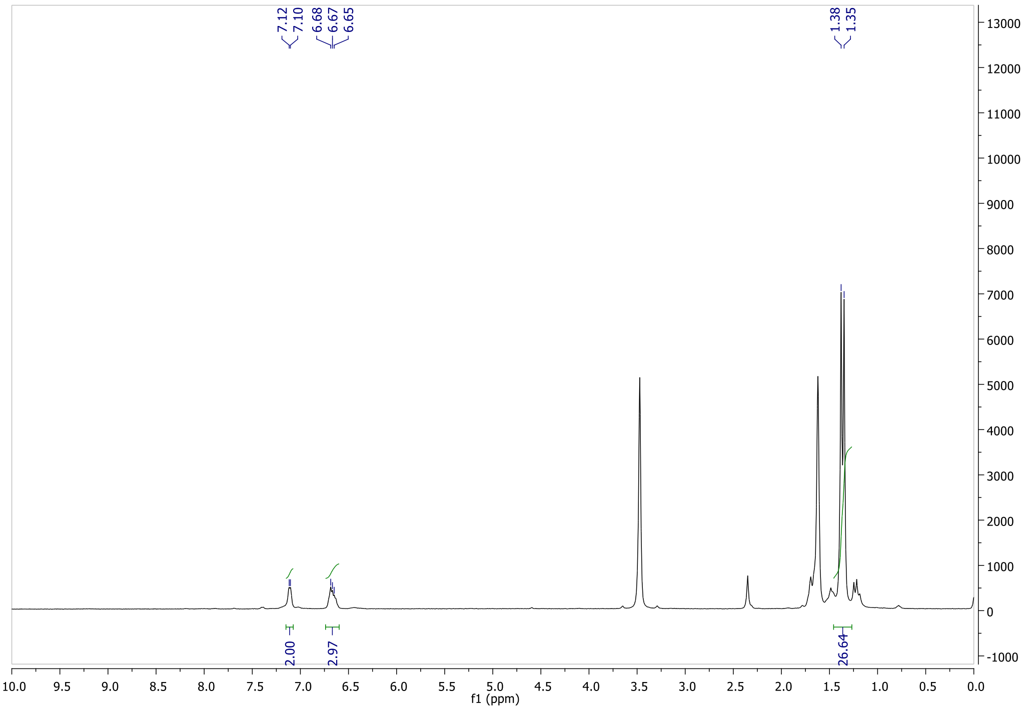

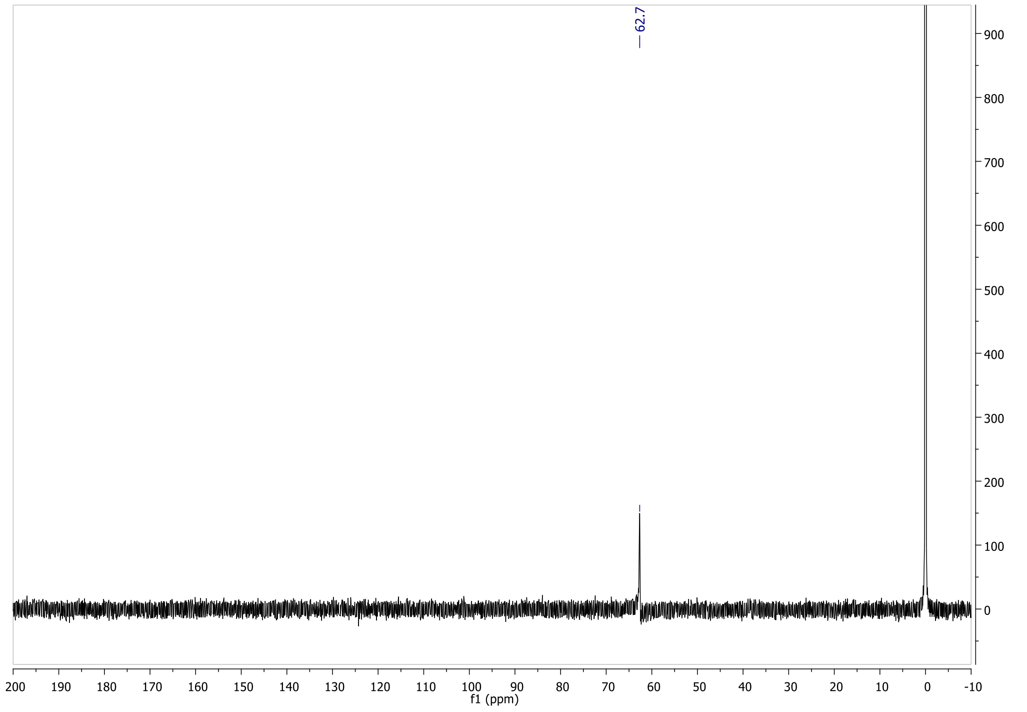

[1] C. Pro, *Agilent Technologies UK Ltd., UK, Xcalibur/CCD system, CrysAlis PRO Software system, Version* **2010**, *1*.

[2] Bruker (2007). SAINT. Bruker AXS Inc.: Wisconsin, USA.

[3] Bruker (2001). SADABS. Bruker AXS Inc.: Wisconsin, USA

[4] a) O. V. Dolomanov, L. J. Bourhis, R. J. Gildea, J. A. K. Howard and H. Puschmann, *J. Appl. Crystallogr.* **2009**, *42*, 339-341; b) G. M. Sheldrick, *Acta Crystallogr A* **2015**, *71*, 3-8.
